# Clinical and electrical outcomes of conduction system pacing versus right ventricular pacing in atrioventricular block: a systematic review and meta-analysis

**DOI:** 10.1186/s12872-026-05930-6

**Published:** 2026-05-06

**Authors:** Faizan Ahmed, Ayesha Zulfiqar, Ramsha Ali, Hafsa Arshad Azam Raja, Omama Ayatullah, Omar Kamel, Arsalan Ahmed, Haris Bin Tahir, Faseeh Haider, Muhammad Faiq Akram, Maryam Abbas Malik, Rubiya Ali, Sana Altaf, Haider Hussain Shah, Madeeha Shafqat, Daniel Aziz, Fawaz Alenezi, Eran S. Zacks

**Affiliations:** 1 Hackensack Meridian Health Jersey, Shore University Medical Center, Neptune, NJ USA; 2 Dow University of Health Sciences, Karachi, Pakistan; 3 Peoples University of Medical and Health Sciences, Nawabshah, Pakistan; 4 Rawalpindi Medical University, Rawalpindi, Pakistan; 5 Dow Medical College, Karachi, Pakistan; 6 Department of Physical Therapy, South Valley University, Qena, Egypt; 7 Liaquat University of Medical and Health Sciences, Jamshoro, Pakistan; 8 Lahore General Hospital, Lahore, Pakistan; 9 Allama Iqbal Medical College, Lahore, Pakistan; 10 Services Institute of Medical Sciences, Lahore, Pakistan; 11 Memorial Healthcare System, Hollywood, FL USA; 12 Deccan College of Medical Sciences, Hyderabad, India; 13 Bayhealth Hospital, Kent Campus, Dover, DE USA; 14 Geisinger Medical Center, Danville, PA USA; 15 Morristown Medical Center, Gagnon Cardiovascular Institute, Morristown, NJ USA; 16 Division of Cardiology, Duke University Hospital, Durham, NC USA; 17 Department of Electrophysiology, Hackensack Meridian Health, Jersey Shore University Medical Center, Neptune, NJ USA

**Keywords:** Conduction system pacing, Right ventricular pacing, Atrioventricular block, Left bundle branch area pacing, His bundle pacing

## Abstract

**Introduction:**

Right ventricular pacing (RVP) is the conventional treatment for atrioventricular (AV) block, but may cause ventricular dyssynchrony and adverse remodeling. Conduction system pacing (CSP) has emerged as a physiologic alternative. This meta-analysis compared the efficacy, safety, echocardiographic, and electrical outcomes of CSP versus RVP in patients with AV block.

**Methods:**

From 2,688 records identified in PubMed, Cochrane, Embase, and ScienceDirect, 19 studies were included. Pooled efficacy, safety, echocardiographic, and procedural outcomes were analyzed using a random-effects model in R (v4.5.1), with meta-regression assessing follow-up effects; *p* < 0.05 was considered significant.

**Results:**

Nineteen studies comprising 5,390 patients (2,182 CSP; 3,208 RVP) were analyzed. Compared with RVP, CSP was associated with a reduction in all-cause mortality (RR 0.50, p < 0.0001), heart failure hospitalization (RR 0.39, p < 0.0001), pacing-induced cardiomyopathy (RR 0.36, p = 0.039), and the primary composite outcome (RR 0.44, p < 0.0001). Cardiovascular death, cardiac resynchronization therapy upgrade, and biventricular pacing upgrade did not differ significantly between groups. CSP was associated with improved left ventricular ejection fraction (MD +2.60%, p < 0.0001) and reduced left ventricular end-diastolic diameter (MD −1.54 mm, p < 0.0001) compared with RVP. Shorter paced QRS duration at implantation and follow-up was observed with CSP compared with RVP, indicating superior ventricular synchrony, although procedural and fluoroscopy times were longer. Meta-regression indicated that LVEF improvement decreased with longer follow-up, while LVEDD reduction remained consistent.

**Conclusion:**

CSP was associated with lower all-cause mortality, HF hospitalization, and pacing-induced cardiomyopathy, while improving ventricular function without additional device-related complications compared with RVP; however, these findings should be interpreted in the context of predominantly observational data.

**Supplementary Information:**

The online version contains supplementary material available at 10.1186/s12872-026-05930-6.

## Introduction

Atrioventricular (AV) block is defined as an interruption or delay in the impulse transmission from the atria to the ventricles due to an anatomic or functional impairment in the conduction system of the heart [1]. Anatomically, AV blocks are either supra-nodal or infra-nodal (intra/infra-Hisian) blocks. Infra-nodal blocks significantly delay the impulse to the ventricles and can progress to complete heart block, and hence require a pacemaker. Complete AV blocks affect approximately 0.02–0.04% of the population [2].

Traditionally, right ventricular pacing (RVP) has been the standard approach to permanent cardiac pacing in patients with AV block. It involves placing a lead in the right ventricular endocardium, producing a single focal depolarization that spreads slowly via myocyte-to-myocyte conduction, leading to non-physiologic, dyssynchronous ventricular activation [3]. Despite its proven efficacy and long-term durability, RVP has long been associated with adverse clinical effects including pacing-induced cardiomyopathy, left ventricular dysfunction, congestive heart failure, atrial arrhythmias and a higher incidence of mortality [3–5].

Over the past decade, conduction system pacing (CSP) has emerged as a promising therapeutic approach to minimize the risk of developing systolic heart failure. Conduction system pacing (CSP) encompasses novel pacing approaches, including His bundle pacing (HBP) and left bundle branch area pacing (LBBAP), which utilize the native cardiac conduction system to allow rapid impulse conduction and more synchronous ventricular contraction [3]. HBP works by directly stimulating the His bundle. Although it closely replicates the native conduction system, it has certain limitations, including high and sometimes unstable capture thresholds and low ventricular sensing (small R-wave amplitudes). In contrast, LBBAP activates the native conduction system by stimulating the proximal left bundle of the His–Purkinje network, demonstrating low and stable pacing thresholds along with strong ventricular sensing [3, 6]. Several contemporary studies, including the CSPACE trial, have suggested that CSP is associated with reductions in adverse outcomes such as pacing-induced cardiomyopathy, heart failure hospitalization, and the need for upgrade to cardiac resynchronization therapy when compared with RVP [4]. Large multicenter observational cohorts have also reported that LBBAP is associated with improved left ventricular function, narrower QRS duration, and lower composite clinical event rates than RVP in patients with AV block [7]. Additionally, ongoing randomized trials such as LEAP-BLOCK continue to build evidence that CSP may offer superior long-term clinical and echocardiographic outcomes [8].

Previous meta-analyses have compared CSP and RVP, but most were limited by smaller sample sizes, fewer randomized data, and narrower outcome assessment [9, 10]. Despite these emerging data, evidence remains fragmented, and no comprehensive meta-analysis has systematically compared CSP with RVP in patients with AV block. Our study incorporates newer randomized trials, larger contemporary LBBAP cohorts, and evaluates both clinical and electrical endpoints, thereby providing a more comprehensive synthesis of current evidence. Therefore, the aim of this meta-analysis is to evaluate the efficacy, safety, echocardiographic parameters, and procedural outcomes of CSP versus RVP in patients with AV block.

## Methods

### Study design and protocol registration

This systematic review was in accordance with the guidelines set by the Cochrane Collaboration and the Preferred Reporting Items for Systematic Reviews and Meta-Analyses (PRISMA) framework [11]. The study protocol was registered in the International Prospective Register of Systematic Reviews (PROSPERO) under registration number (CRD420251268378).

### Search strategy and data sources

A comprehensive electronic search was conducted in PubMed, Cochrane, Embase, and ScienceDirect from inception to October 2025, with no language restrictions. The following MeSH terms and keywords were used: “Atrioventricular Block”[Mesh], “Heart Conduction System”[Mesh], and “Pacemaker, Artificial”[Mesh]. The keywords included “conduction system pacing” OR “His bundle pacing” OR “His-bundle pacing” OR HBP OR “selective His pacing” OR “nonselective His pacing” OR “His–Purkinje conduction pacing” OR “left bundle branch pacing” OR “left bundle branch area pacing” OR LBBP OR LBBAP OR “LBB area pacing” OR “deep septal pacing” OR “left ventricular septal pacing”. The search strategies employed for each database are detailed in Supplementary Table 1.

### Study selection and eligibility criteria

All records obtained from the electronic database search were uploaded into Rayyan AI software to facilitate systematic screening, and duplicate entries were removed prior to review. The selection process was carried out in two stages. Initially, studies were evaluated based on their titles and abstracts to exclude clearly irrelevant articles. Full-text papers were then retrieved for detailed assessment and two reviewers (AZ) and (AA) independently examined the full texts to determine final eligibility, applying prespecified inclusion and exclusion criteria. Any inconsistencies in judgment were resolved through discussion, and unresolved disagreements were settled by a third reviewer (FA) to ensure consistency and transparency in study selection.

#### Inclusion criteria

Studies were considered eligible for inclusion in this systematic review and meta-analysis if they met the following criteria: (1) randomized controlled trials or observational cohort studies; (2) adult patients diagnosed with atrioventricular (AV) block; (3) investigated conduction system pacing (CSP) modalities, including His bundle pacing (HBP) or left bundle branch pacing (LBBP)/left bundle branch area pacing (LBBAP); (4) included a control group receiving right ventricular pacing (RVP), including apical, septal, or right ventricular outflow tract (RVOT) pacing; and (5) reported outcomes of interest including efficacy, safety, echocardiographic, and procedural outcomes.

#### Exclusion criteria

Exclusion criteria included: (1) letters to the editor, animal studies, clinical guidelines, case reports, or case series; (2) studies involving populations other than atrioventricular block; (3) studies focusing on other interventional modalities; and (4) single-arm studies without a comparator group.

### Outcomes assessed

This systematic review and meta-analysis assessed the following outcomes of interest. Efficacy outcomes included primary composite outcome, all-cause mortality, cardiovascular death (CV death), cardiac resynchronization therapy (CRT) upgrade, hospitalization, pacing-induced cardiomyopathy (PICM), and biventricular pacing (BiVP) upgrade. Safety outcomes included lead-related complications, pneumothorax, pericardial effusion, and pocket-related complications. Echocardiographic outcomes included left ventricular ejection fraction (LVEF), left atrial dimension (LAD), and left ventricular end-diastolic dimension (LVEDD). Procedural and electrical outcomes included procedural time, fluoroscopy time, R-wave amplitude, impedance, paced QRS duration, and pacing threshold.

### Data extraction

Data were extracted using a standardized form, capturing details such as study design, participant characteristics, treatment protocols, and outcomes. Two authors independently collected this data to minimize bias. Discrepancies were resolved through discussion and agreement. All data were compiled and organized in Microsoft Excel for analysis.

### Quality assessment

Quality assessment of all included studies was independently conducted by two investigators using the Newcastle–Ottawa Scale (NOS) for observational studies and the Cochrane Risk of Bias (RoB) tool for randomized controlled trials [12], with results compared and verified. For observational cohorts, the appropriate version of the NOS was applied, awarding a maximum of nine stars across three domains: Selection (up to 4 stars), Comparability (up to 2 stars), and Outcome/exposure (up to 3 stars). For randomized controlled trials, the revised Cochrane Risk of Bias tool (RoB 2) was used to classify studies as low risk of bias, some concerns, or high risk of bias across five domains: randomization process, deviations from intended interventions, missing outcome data, outcome measurement, and selective reporting. Assessments were performed independently by two reviewers, and disagreements were resolved through discussion. A third reviewer was consulted when consensus could not be reached. This rigorous appraisal ensured the credibility and reliability of the included studies.

### Statistical analysis

The meta-analysis was conducted using R software with the *meta* package (version 4.5.1). Results were visualized using forest plots. Binary outcomes were analyzed using risk ratios (RRs), while continuous outcomes were assessed using mean differences (MDs). Pooled estimates were calculated using a random-effects model. Statistical heterogeneity was assessed using Cochran’s Q statistic and the I² index. I² values of < 25%, 25–50%, and > 50% indicated low, moderate, and high heterogeneity, respectively [13]. Between-study variance was also quantified using Tau-squared (τ²). The DerSimonian and Laird random-effects model was applied for all outcomes [14]. A p-value of < 0.05 was considered statistically significant. The robustness of pooled estimates was evaluated using leave-one-out sensitivity analysis, in which each study was sequentially excluded and the analysis repeated to assess the influence of individual studies. Subgroup analyses were performed based on CSP modality (LBBAP, HBP, and mixed CSP [LBBAP + HBP]) and study design (randomized controlled trials vs. observational cohort studies). Meta-regression analyses were conducted using a random-effects model with the meta package to assess the impact of follow-up duration on echocardiographic outcomes. Additional meta-regression analyses for procedural outcomes were performed using covariates including age, sex, comorbidities (atrial fibrillation, diabetes, hypertension), and left ventricular ejection fraction to explore potential sources of heterogeneity.

## Results section

### Search results

**Fig. 1:**
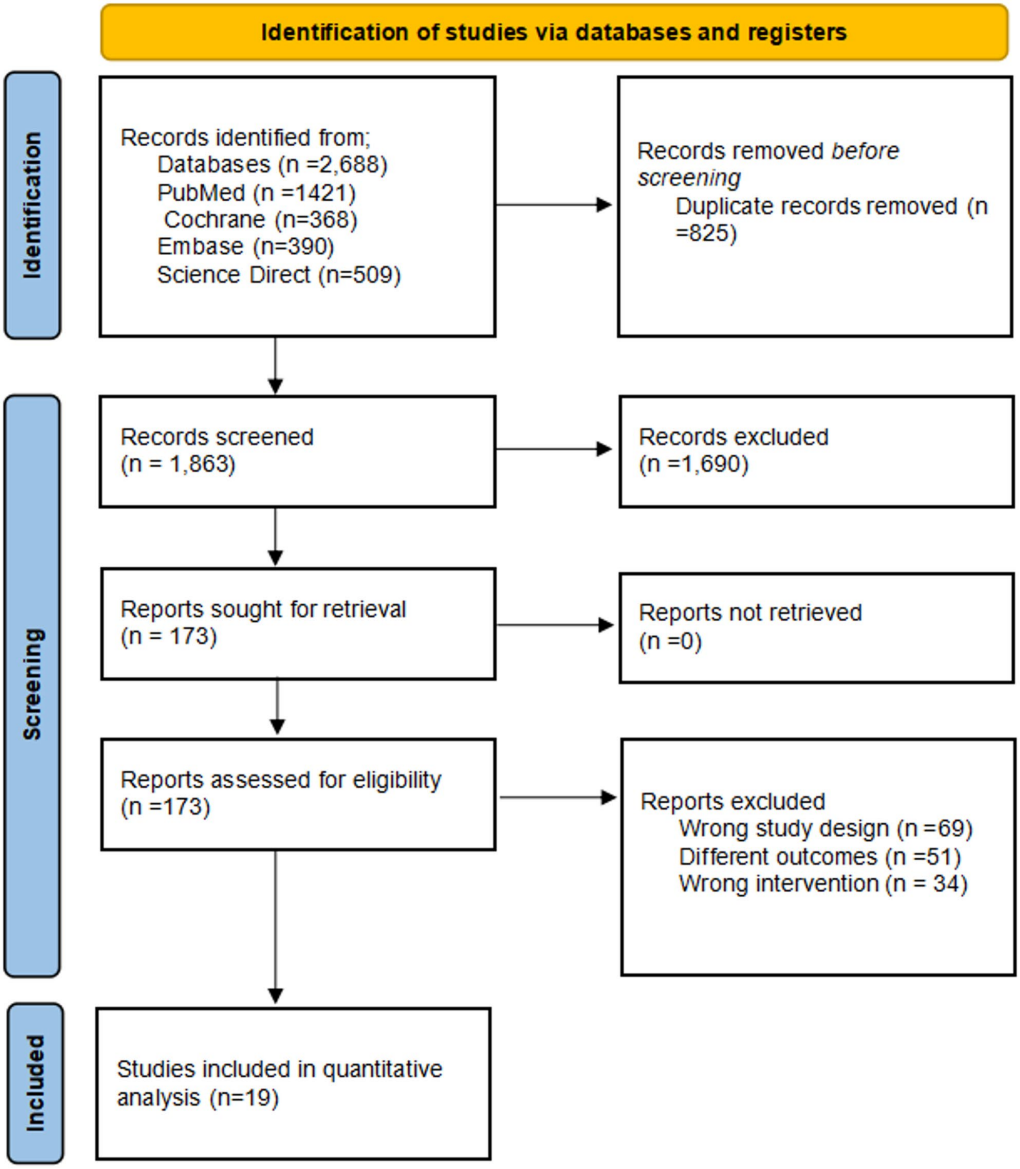
Preferred Reporting Items for Systematic Reviews and Meta-Analyses (PRISMA) flowchart illustrating the study selection process

From 2,688 records identified across four databases of PubMed, Cochrane, Embase and Science Direct, 825 duplicates were removed, leaving 1,863 unique records screened. Of these, 1,690 were excluded at title or abstract level. 173 reports were retrieved and assessed at full text; 154 were excluded predominantly due to wrong study design, resulting in 19 studies included in the quantitative synthesis [4, 15–30]. Figure 1 presents the PRISMA diagram outlining study identification and inclusion.

### Study characteristics

Nineteen studies were included (total combined participants across all studies = 5,390), with 2,182 participants in the CSP arms and 3,208 participants in the RVP arms. The 19 studies were conducted across nine countries/locations: China, Japan, Korea, Italy, Greece, Spain, Australia, Singapore, and Poland (one study reported as multicentre without a single country listed). Publication years span from 2020 to 2025. Detailed study characteristics and baseline patient demographics are presented in Tables 1 and 2.

**Table 1:** Study characteristics

Study author & year	Study Design	Follow up (year)	I/C	Country	Total no. Of Participants		
					Total	(I) CSP	(C) RVP
Bertini et al. 2025 [15]	Prospective trial	2 years	LBBAP/RVP	Italy	344	172	172
Curila et al. 2025 [16]	Randomized Controlled Trial.	1 year	CSP/RVP	multicentre	249	124	125
Chen et al. 2023 [8]	Cohort observational	1 year	LBBAP/RVP	China	903	393	510
Li et al. 2021 [7]	Prospective observational	6 months	LBBAP/RVP	China	366	235	120
Leventopoulos et al. 2024	Prospective cohort	3, 6 & 12 months	LBBP/RVP	Greece	38	20	18
Chen et al. 2024 [18]	observational	1, 3 & 6 months	CSP/RVP	China	323	77	246
Kono et al. 2024 [19]	Retrospective cohort	1,3, 6 & 12 months	LBBAP/HBP/ RVAP	Japan	424	75	296
Matos et al. 2024 [20]	Randomized Controlled Trial	3 & 6 months	CSP/RVP	Spain	75	40	35
Yang et al. 2024 [21]	Retrospective cohort	1, 6 & 12 months	LBBAP/RVA/ RVS	China	40	16	RVA-11 RVS-13
Zhang et al. 2024 [22]	Prospective observational	3, 6 & 12 months	LBBAP/RVP	China	86	43	43
Yeon et al. 2025	Retrospective cohort	1 year	LBBAP/RVP	Korea	738	243	495
Tan et al. 2022	Multi centerobservational	1, 3 & 6 months	CSP/RVP	Singapore	860	282	578
Michalik et al. 2021 [26]	Prospective observational	6 months	RVP/HBP	Poland	50	24	26
Loong et al. 2025	Randomized Controlled Trial.	3, 6 & 12 months	RVsP/CSP	Australia	202	101	101
Okubo et al. 2024 [27]	Multi centerobservational	1 year	LBBAP/RSVP	Japan	161	82	79
Wang et al. 2024 [28]	Retrospective observational	3, 6 & 12 months	LBBAP/RVP	China	267	109	158
Shimeno et al. 2025 [29]	Retrospective observational	1,3, 6 & 12 months	RVP/LBBAP LBBP/LSVP	Japan	147	110	137
Inoue et al. 2025 [30]	Retrospective observational	1 year	LBBAP/RVP	Japan	15	7	8
Zhang et al. 2020	Retrospective observational	12–24 months	LBBP/RVAP	China	66	29	37

*I/C* Intervention/Control, *LBBAP* Left Bundle Branch Area Pacing, *RVP* Right Ventricular Pacing, *CSP* Conduction System Pacing, *HBP* His Bundle Pacing, *LBBP* Left Bundle Branch Pacing, *LSVP* Left Septal Ventricular Pacing, *RVS* Right Ventricular Septum, *RVA* Right Ventricular Apex

**Table 2:** Baseline characteristics

Study author et al., year	Age (years)		Sex (M/F)		Comorbidities, *n*				Indication for pacing, *n* (%)		Electrocardiography		Echocardiography			
	CSP	RVP	CSP	RVP	AF (I/C)	HTN (I/C)	DM (I/C)	CAD (I/C)	SR with AVB (I/C)	Perm-AF with high degree AVB (I/C)	QRS duration (ms)		LVEDD (mm)		LVEF %	
											CSP	RVP	CSP	RVP	CSP	RVP
Bertini et al. 2025 [15]	58.2 ± 4.1	58.9 ± 3.8	109/63	106/66	33/31	79/75	NR	77/71	NR	NR	NR	NR	NR	NR	NR	NR
Curilaet.al 2025	76 ± 7	75 ± 9	89/36	93/31	22/17	96/96	47/44	NR	NR	NR	NR	NR	NR	NR	6166	6165
Chen et al. 2023 [8]	71.7 (Mean)	73 (Mean)	240/153	311/199	65/60	226/228	91/128	75/92	NR	NR	NR	NR	97:62 ± 52:29	105:73 ± 47:48	61.90(56.40-65.65)	60.00(50.10-65.52
Li et al. 2021	63.3 ± 15.0	62.1 ± 17.2	150/85	81/39	72/19	132/65	50/25	31/20	71/30		115.9 ± 26.7	117.9 ± 27.9	49.4 ± 6.6	49.6 ± 5.9	61.7 ± 7.4	61.5 ± 6.4
Leventopoulos et al. 2024	80.5 median	81.5 median	12/8	13/5	NR	18/16	6/6	NR	NR	NR	121.8(4)	151.5(4.3)	47.5(0.9)	49.5(1.0)	56.4(1.4)	53.9(1.5)
Chen et al. 2024 [18]	71.19 ± 9.9	73.8 ± 2.2	38/39	113/133	70/110	248/356	NR	NR	NR	NR	NR	NR	48.37 ± 4.57	48.37 ± 4.57	57.32 ± 2.18	57.41 ± 2.42
Kono et al. 2024 [19]	LBBAP = 78.33 ± 11.34 HBP = 78.33 ± 7.62	77.3 ± 8.9	39/36	160/136	10/95	50/191	23/73	NR	61/155	1/9	118.33 ± 37.04	110 ± 37.25	NR	NR	NR	NR
Matos et al. 2024 [20]	75 ± 10.2	80 ± 6.9	26/14	23/12	13/7	32/31	20/11	7/11	NR	NR	136.6 ± 25.5	138.2 ± 21.5	48.3 ± 6.3	47.8 ± 3.7	58.5 ± 7.6	60.7 ± 6.9
Yang et al. 2024 [21]	68.1 ± 14.7	RVA = 67.6 ± 15.4 RVS = 75.0 ± 9.01	5/11	RVA = 5/6 RVS = 7/6	NR	11/8	10/5	NR	NR	NR	NR	NR	49.46 ± 4.10	52.06 ± 8.61	67.31 ± 7.02	62.39 ± 8.89
Zhang et al. 2024 [22]	75.0 ± 10.6	72.4 ± 10.0	28/15	26/17	NR	36/33	13/9	11/7	NR	NR	116.3 ± 26.2	113.1 ± 27.2	48.3 ± 5.2	49.2 ± 4.3	62.2 (2.5%)	63.3 (5.2%)
Yeon et al. 2025	71.5 ± 13.4)	72.5 ± 11.6,	130/113	251/244	NR	NR	NR	NR	NR	NR	124.7 ± 32.6	112.3 ± 28.4	49.6 ± 6.0	49.8 ± 6.1	61.9 ± 7.3	60.9 ± 6.9
Tan et al. 2022	75 ± 11	74 ± 11	130/152	280/298	90/231	230/440	122/211	91/216	173/238	NR	NR	NR	NR	NR	59 ± 8	59 ± 7
Michalik et al. 2021 [26]	73.0 ± 14.4	77.2 ± 6.7;	13/11	15/11	17/10	16/17	8/11	6/8	11/18	13/8	121.7 ± 23.4	108.1 ± 15.6	NR	NR	NR	NR
Loong et al. 2025	77.0 ± 8.1	77.2 ± 9.3	65/36	70/31	32/35	86/80	39/42	26/22	94/87	7/14	NR	NR	NR	NR	NR	NR
Okubo et al. 2024 [27]	76.7 ± 12.0	77.1 ± 11.5	45/37	42/37	34/33	46/47	26/27	21/20	47/45	10/10	124.1 ± 28.5	125.1 ± 27.7	NR	NR	60.9 ± 7.8	60.9 ± 6.9
Wang et al. 2024 [28]	80.7 ± 4.1	80.8 ± 4.0	54/55	92/66	43/70	76/121	13/41	37/50	NR	NR	108.1 ± 23.7	96.2 ± 21.8	47.6 ± 5.5	46.4 ± 4.9	61.3 ± 6.3	62.5 ± 4.3
Shimeno et al. 2025 [29]	78 ± 10	78 ± 10	52/58	69/68	4/26	81/95	19/38	NR	98/101	2/17	110 ± 27	116 ± 27	NR	NR	NR	NR
Inoue et al. 2025 [30]	79 ± 7.35	72 ± 8.94	1/6	5/3	NRhe	6/3	2/1	1/1	NR	NR	111.33 ± 4.59	142.43 ± 9.11	NR	NR	58.97 ± 4.87	55.2 ± 5.54
Zhang et al. 2020	63.60 ± 8.80	67.40 ± 8.81	13/16	17/20	4/3	17/16	9/6	7/12	NR	NR	104.83 ± 15.41	98.86 ± 7.33	48.71 ± 3.27	46.92 ± 4.931	55.08 ± 4.32	56.29 ± 5.40

*HTN* hypertension, *AVB *atrioventricular block, *DM* diabetes mellitus, *CAD* coronary artery disease, *AF* atrial fibrillation, *SR* sinus rhythm, *perm* permanent,* CSP* conduction system pacing, *RVP* right ventricular pacing, *LVEDD* left ventricular enddiastolic diameter, *LVEF* left ventricular ejection fraction

### Bias assessment

The methodological quality of observational studies was assessed using the Newcastle–Ottawa Scale (NOS), with the majority demonstrating high quality. All included cohort studies achieved NOS scores of 8–9, indicating low overall risk of bias across selection, comparability, and outcome domains, with no study rated as poor quality. Randomized studies were evaluated using the Cochrane Risk of Bias 2.0 (ROB-2) tool, where two trials (Loong et al., 2025; Curila et al., 2025) were judged to have an overall low risk of bias across all domains. Two studies (Bertini et al., 2025; Matos et al., 2024) were rated as having some concerns, primarily related to the randomization process and deviations from intended interventions in Bertini et al. (2025), and missing outcome data in Matos et al. (2024). No study was assessed as having a high risk of bias. Overall, the risk of bias across the included evidence was low, with only minor and domain-specific concerns. A detailed breakdown of the Newcastle–Ottawa Scale (NOS) quality assessment for observational studies and the Cochrane Risk of Bias 2.0 (ROB-2) for randomized trials are provided in Supplementary Tables 2 and Supplementary Figure S1.

### Outcomes

#### Primary outcomes

##### Efficacy outcomes

**All-cause mortality**

**Fig. 2:**
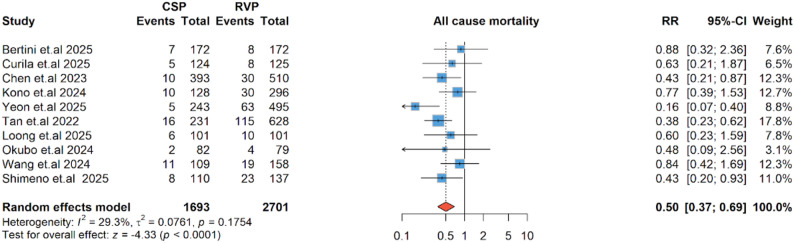
Forest plot of all-cause mortality

Ten studies were included in this analysis, with 1,693 patients receiving CSP and 2,701 patients receiving RVP, comprising 4,394 participants. The pooled RR was 0.50 (95% CI: 0.37–0.69, *p* < 0.0001), indicating a statistically significant 50% reduction in all-cause mortality associated with CSP (Fig. 2). Moderate heterogeneity was observed (I² = 29.3%), but sensitivity analyses confirmed the robustness of the effect, with consistent directionality and statistical significance across all analyses (Fig. S2). These findings suggest a potential association between CSP and improved survival compared with RVP. The LFK index reported was 1.26, indicating minor asymmetry. This suggests a small but detectable deviation from symmetry in the distribution of study effects around the pooled estimate. Similar findings of slight asymmetry were observed in the DOI plot for this outcome (Fig. S3).

**Cardiovascular (CV) death**

This outcome was assessed across 6 studies, involving 1,142 patients in the CSP group and 1,677 patients in the RVP group, totalling 2,819 participants. The pooled RR was 0.74 (95% CI: 0.36–1.49, *p* = 0.3963), indicating a non-significant trend toward reduced cardiovascular mortality with CSP (Fig. S4), heterogeneity was absent (I² = 0%). While CSP may offer a potential benefit in reducing cardiovascular death, the current evidence does not support a definitive conclusion. Significant skew in the plot reveals an extreme asymmetry of publication bias (Fig. S5).

**Cardiac Resynchronization Therapy (CRT)**

Seven studies contributed to the CRT upgrade analysis, with 1,188 patients in the CSP group and 1,720 patients in the RVP group, comprising 2,908 participants. Similar to the BiVP upgrade findings, no events were observed in the CSP group, while events occurred in the RVP group. The pooled RR was 0.00 (95% CI: 0.00–3.72, *p* = 0.1068), and heterogeneity was absent (I² = 0%) (Fig. S6). Although CSP appears to be associated with a lower risk of CRT upgrade, the absence of events and wide confidence intervals limit the strength of inference. No asymmetry is noted for this outcome (Fig. S7).

**HF hospitalization**

**Fig. 3:**
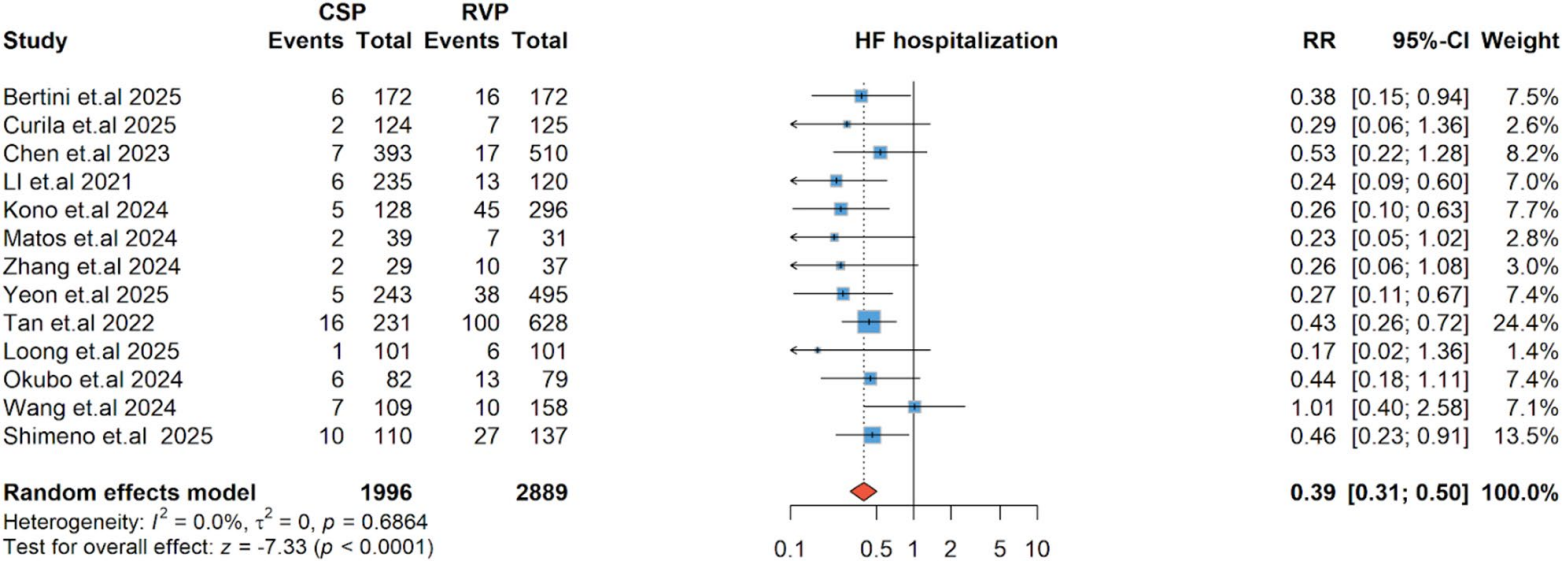
Forest plot of HF hospitalization

A total of 13 studies contributed to this analysis, with 1,996 patients receiving CSP and 2,889 patients receiving RVP, yielding a combined sample of 4,885 individuals. The pooled RR was 0.39 (95% CI: 0.31–0.50, *p* < 0.0001), reflecting a statistically significant 61% reduction in heart failure hospitalization risk with CSP, and heterogeneity was absent (I² = 0%) (Fig. 3). These findings demonstrate consistent directionality across studies, suggesting a potential benefit of CSP in reducing heart failure-related hospitalizations; however, the presence of asymmetry indicates possible small-study effects and warrants cautious interpretation (Fig. S8).

**Pacing induced cardiomyopathy (PICM)**

Four studies evaluated the incidence of PICM, with 554 patients in the CSP group and 971 in the RVP group. The pooled RR was 0.36 (95% CI: 0.14 to 0.95, *p* = 0.0392), favoring CSP. Heterogeneity was moderate to high (I² = 77.9%, τ² = 0.6487) (Fig. S9). Sensitivity analysis revealed that omitting *Loong et al. 2025* reduced heterogeneity to 0% and strengthened the effect (RR = 0.21, *p* < 0.0001) (Fig. S10). These results suggest that CSP may significantly reduce the risk of PICM, though the effect is sensitive to individual study influence.

**Primary composite outcomes**

**Fig. 4:**
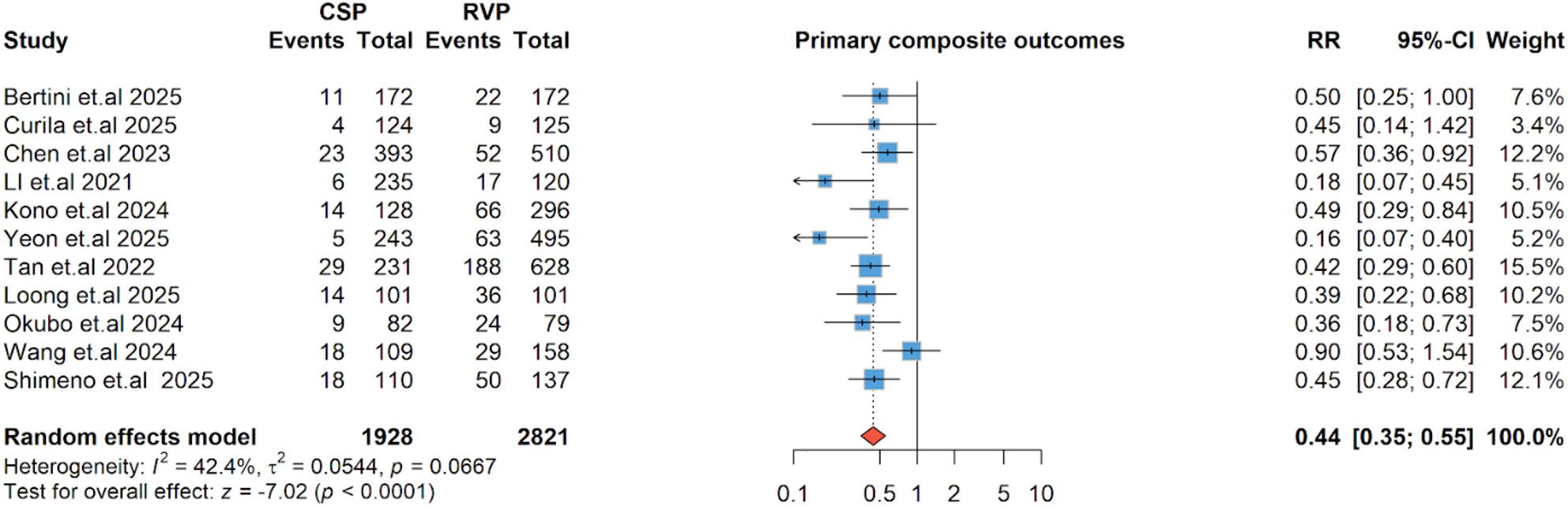
Forest plot of primary composite outcomes

This meta-analysis included 11 studies comparing primary composite outcomes between CSP and RVP, with 1,928 patients in the CSP group and 2,821 in the RVP group. The pooled RR was 0.44 (95% CI: 0.35 to 0.55, *p* < 0.0001), favoring CSP. Moderate heterogeneity was observed (I² = 42.4%, τ² = 0.0544) (Fig. 4). Sensitivity analysis confirmed the robustness of this effect, with RR estimates ranging from 0.42 to 0.47 across exclusions and consistently significant p-values; heterogeneity was reduced to 12.5% after omitting Wang et al. (2024) (Fig. S11). These findings suggest that CSP is associated with a significantly lower risk of adverse composite outcomes compared with RVP. Minor asymmetry is present, suggesting a slight deviation from symmetry in publication bias (Fig. S12 and Table S3).

**Biventricular Pacing (BiVP) upgrade**

This outcome was evaluated in 5 studies, comprising 890 patients in the CSP group and 1,341 patients in the RVP group, totaling 2,231 participants. No BiVP upgrades were reported in the CSP group across all studies, while events occurred in the RVP group. The pooled RR was 0.00 (95% CI: 0.00–16.03, *p* = 0.1802), reflecting the absence of events in CSP. However, the wide confidence interval and non-significant p-value indicate substantial uncertainty. The overall heterogeneity was negligible (I² = 0%). While the data suggest a lower likelihood of BiVP upgrade with CSP, the evidence remains inconclusive due to sparse event rates (Fig. S13).

**Subgroup analysis of primary outcomes**

Upon subgroup analysis for all-cause mortality, stratification by CSP modality showed significant reductions with LBBAP (RR = 0.48, 95% CI 0.31–0.75, I² = 37.9%) and mixed CSP (RR = 0.44, 95% CI 0.29–0.66, I² = 0%), while HBP, reported by a single study, did not differ significantly. By study design, RCTs suggested a non-significant trend, whereas cohort studies demonstrated a significant reduction (RR = 0.46, 95% CI 0.31–0.68, I² = 44.8%) (Fig. S53 and S54). For primary composite outcomes, stratification by CSP modality showed significant reductions with LBBAP (RR = 0.41, 95% CI 0.28–0.60, I² = 60.3%) and mixed CSP (RR = 0.41, 95% CI 0.31–0.55, I² = 0%), while HBP did not differ significantly. Stratification by study design showed that RCTs demonstrated a significant reduction with CSP compared with RVP (RR = 0.43, 95% CI 0.29–0.65, I² = 0%), and cohort studies showed a consistent reduction (RR = 0.43, 95% CI 0.31–0.59, I² = 58.8%) (Figs. S55 and S56). For cardiovascular death, subgroup analysis by CSP modality indicated that LBBAP showed a non-significant trend toward lower risk compared with RVP. Mixed CSP and HBP, each assessed in single studies, did not show statistically significant differences. Pooled analysis by study design also yielded non-significant results (Figs. S57 and S58). For CRT outcomes, subgroup analysis demonstrated significant reductions with both LBBAP and mixed CSP compared with RVP. Pooled analysis by study design confirmed a significant reduction, with RCTs showing a marked effect (RR = 0.11, 95% CI 0.02–0.61, I² = 0%) and cohort studies demonstrating similar findings (RR = 0.09, 95% CI 0.02–0.39, I² = 0%) (Figures S59 and S60). Furthermore, subgroup analysis of heart failure hospitalization showed significant reductions with LBBAP (RR = 0.41, 95% CI 0.30–0.56, I² = 11.4%) and mixed CSP (RR = 0.38, 95% CI 0.24–0.59, I² = 0%), while HBP did not differ significantly. Pooled analysis by study design confirmed consistent reductions, with RCTs showing a significant effect (RR = 0.30, 95% CI 0.15–0.58, I² = 0%) and cohort studies demonstrating similar findings (RR = 0.41, 95% CI 0.31–0.54, I² = 0%) (Figs. S61 and S62). For pacing-induced cardiomyopathy (PICM), subgroup analysis showed that LBBAP was associated with a significant reduction compared with RVP (RR = 0.21, 95% CI 0.10–0.45, I² = 0%). In contrast, HBP and mixed CSP cohorts showed no significant benefit. Pooled analysis by study design demonstrated consistent findings, with cohort studies showing a significant reduction (RR = 0.21, 95% CI 0.10–0.43, I² = 0%), while RCTs did not show a significant difference (Figs. S63 and S64). CSP was consistently associated with a lower risk of BiVP upgrade compared with RVP across subgroups (LBBAP, mixed CSP, and HBP) and study designs (RCTs and cohorts) (Figs. S65 and S66). Details of the subgroup analyses are provided in Supplementary Table S4.

#### Secondary outcomes

##### Safety outcomes

The meta-analysis comparing CSP with RVP demonstrated no significant differences across lead-related and procedural complications. Overall lead-related complications were assessed in eight studies, with a pooled RR of 0.78 (95% CI: 0.42–1.45, p = 0.4239), indicating no significant difference between groups; heterogeneity was absent (I² = 0%) (Fig. S14). However, extreme asymmetry was observed (Fig. S15). Pneumothorax risk, assessed in six studies (963 CSP and 1,886 RVP patients), yielded a pooled RR of 1.77 (95% CI: 0.58–5.38, p = 0.3160) with no heterogeneity (I² = 0%) (Fig. S16). Asymmetry bordered the threshold between minor and major, suggesting mild distortion (Fig. S17). Pericardial effusion, evaluated in four studies (770 CSP and 1,420 RVP patients), showed a pooled RR of 0.88 (95% CI: 0.18–4.25, p = 0.8710), with wide confidence intervals and no heterogeneity (I² = 0%) (Fig. S18). Similarly, pocket-related complications across four studies (609 CSP vs. 1,004 RVP patients) demonstrated comparable risks (RR 1.01, 95% CI: 0.29–3.52, p = 0.9852), with negligible heterogeneity (I² = 0%, τ² = 0) (Fig. S19). Overall, CSP did not demonstrate a significant difference in procedural or device-related complications compared with RVP. However, the presence of asymmetry and low event rates limits the certainty of these findings and warrants cautious interpretation.

##### Echo related outcomes

**Fig. 5:**
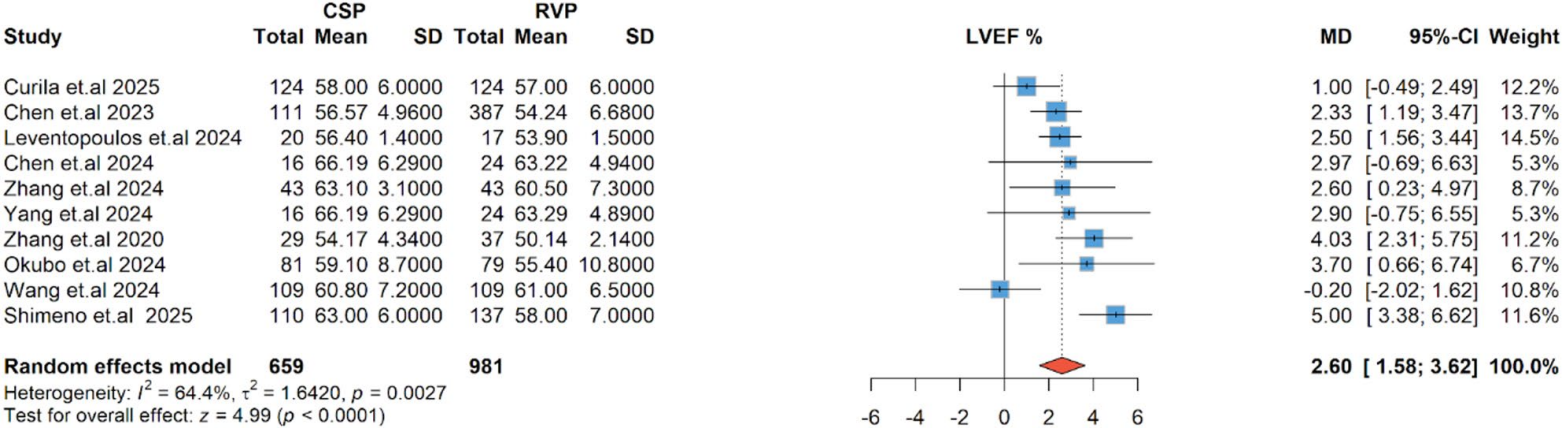
Forest Plot of Left ventricular ejection fraction (LVEF)

Ten studies including 659 CSP and 981 RVP patients assessed left ventricular ejection fraction (LVEF), with the pooled mean difference favoring CSP (MD 2.60%, 95% CI: 1.58–3.62, *p* < 0.0001). Moderate heterogeneity was observed (I² = 64.4%) (Fig. 5), though leave-one-out sensitivity analyses demonstrated consistent significance (MD range: 2.25%–2.91%); omission of Shimeno et al. 2025 reduced heterogeneity (I² = 47.6%), while omitting Wang et al. 2024 increased the effect size (MD 2.91%) and lowered I² to 50.0%, confirming the robustness of CSP’s positive impact on LVEF despite study-level variability (Figs. S20 and S21). Left atrial diameter (LAD), evaluated in two studies (220 CSP and 544 RVP patients), showed no significant difference between groups (MD − 0.14 mm, 95% CI: − 2.65 to 2.37, p = 0.9128), with substantial heterogeneity (I² = 85.9%), indicating inconclusive effects (Fig. S22). In contrast, seven studies assessing left ventricular end-diastolic diameter (LVEDD) (432 CSP and 751 RVP patients) demonstrated a significant reduction favoring CSP (MD − 1.54 mm, 95% CI: − 2.20 to − 0.87, p < 0.0001), with negligible heterogeneity (I² = 0.0%) and stable sensitivity analyses (MD range: − 1.32 to − 1.77 mm), suggesting a durable and consistent structural remodeling benefit of CSP over RVP (Fig. S23).

##### Electrical outcomes

**Impedance**

*At implantation*. In the initial impedance analysis across eight studies (CSP = 1042, RVP = 1627), the pooled mean difference was not statistically significant (MD = 24.58 Ω, 95% CI: − 25.12 to 74.29, p = 0.3324). Heterogeneity was extremely high (I² = 97.2%, τ² = 4699.5362), limiting interpretability (Fig. S24). The wide confidence interval and lack of significance suggest that impedance differences between CSP and RVP may be inconsistent or influenced by device-specific factors. Across all leave-one-out scenarios, the mean differences ranged from 7.99 Ω to 42.38 Ω, and none reached statistical significance (all p > 0.05). Confidence intervals consistently crossed zero, and heterogeneity remained elevated (I² > 86% in all cases), indicating that no single study substantially altered the direction or precision of the overall estimate (Fig. S25). Major asymmetry is noted with steep a negative LFK index (Fig. S26).

*At Follow-up*. Impedance at follow-up was assessed by 12 studies (CSP = 1277, RVP = 2135) demonstrating a significant reduction in impedance with CSP (MD = − 50.38 Ω, 95% CI: − 96.83 to − 3.92, p = 0.0335). However, heterogeneity remained very high (I² = 98.4%, τ² = 5884.1879) (Fig. S27). Leave-one-out sensitivity analysis showed that the effect remained statistically significant across most exclusions, with MDs ranging from − 30.60 to − 60.27 Ω. While the direction of effect consistently favored CSP, the magnitude varied, and heterogeneity persisted, suggesting that impedance outcomes may be influenced by lead type, implantation technique, or measurement protocols (Fig. S28). Substantial asymmetry was noted consistent with publication bias (Fig. S29).

**Ventricular pacing rate**

This meta-analysis evaluated ventricular pacing rates across seven studies at follow-up comparing conduction system pacing (CSP) and right ventricular pacing (RVP), encompassing 644 patients in the CSP group and 900 in the RVP group. The pooled mean difference was 9.01 (95% CI: − 5.68 to 23.70), with no statistically significant difference between groups (*p* = 0.2294). Heterogeneity was extremely high (I² = 99.5%, τ² = 389.1043), indicating substantial variability across studies. Notably, one study (Kono et al. 2024) reported a markedly higher pacing rate in the CSP group (MD = 53.60%, 95% CI: 50.59 to 56.61), which may have disproportionately influenced the overall estimate. The remaining studies showed minimal differences between groups, with confidence intervals crossing zero (Fig. S30). Major asymmetry is noted with high positive LFK index (Fig. S31).

**R-wave amplitude**

*At implantation*. Ten studies (CSP: *n* = 1233; RVP: *n* = 1870) assessed R-wave amplitude. The pooled mean difference was not statistically significant (MD: 0.79 mV; 95% CI: −1.37 to 2.95; *p* = 0.4739), with high heterogeneity (I² = 97.1%, τ² = 11.77). Although individual studies such as Chen et al. and Okubo et al. reported higher amplitudes with CSP, the overall effect did not reach significance, suggesting comparable sensing performance between pacing modalities. Exclusion of individual studies did not materially alter the pooled effect estimate. The overall mean difference remained non-significant (MD: 0.79 mV; 95% CI: −1.37 to 2.95; *p* = 0.4739), with consistently high heterogeneity (I² ≈ 97%). Notably, omission of Michalik et al. 2021 yielded a statistically significant result (MD: 1.61 mV; 95% CI: 0.02 to 3.21; *p* = 0.0478), suggesting this study may have attenuated the overall effect (Fig. S32, S33, and S34).

*At Follow-up (mV)*. Across 14 studies (CSP = 1,628; RVP = 2,515), the pooled mean difference in R-wave amplitude was 1.28 mV (95% CI: − 0.87 to 3.42, *p* = 0.2426), with no statistically significant difference. Heterogeneity was extremely high (I² = 99.5%, τ² = 16.45). Sensitivity analysis confirmed instability, with MDs ranging from 0.66 mV to 1.91 mV and p-values consistently > 0.22, except for one borderline significant result (*p* = 0.0479). These findings suggest inconsistent sensing advantages with CSP, and the pooled effect is not robust. Significant asymmetry is seen consistent with potential bias (Fig. S35, S36, and S37).

**Pacing threshold**

*At implantation*. Eleven studies (CSP: *n* = 1234; RVP: *n* = 1928) were included in the analysis of pacing thresholds. The pooled mean difference was not statistically significant (MD: 0.99 V; 95% CI: −0.90 to 2.88; *p* = 0.3040), however, interpretation is limited by high heterogeneity (I² = 99.9%, τ² = 3.92). While some studies (e.g., Michalak et al., Tan et al.) reported higher thresholds with CSP, others showed lower or comparable values, indicating inconsistent findings across cohorts (Fig. S38 and S39).

*At Follow-up.* Fourteen studies contributed to the analysis of pacing thresholds, with 1,691 CSP and 2,332 RVP patients. The pooled mean difference was 0.07 V (95% CI: − 0.06 to 0.20, *p* = 0.3005), indicating no significant difference between CSP and RVP. Heterogeneity was substantial (I² = 95.2%, τ² = 0.0619). Leave-one-out analysis showed MDs ranging from 0.01 V to 0.10 V, with all confidence intervals crossing zero and p-values > 0.24. These results suggest that pacing thresholds are comparable between the two modalities, with variability likely driven by lead type and implantation technique. Minor asymmetry is noted with LFK index of 1.27 (Fig. S40, S41, and S42).

**Paced QRS duration**

*At implantation*. A total of 11 studies (CSP: *n* = 1309; RVP: *n* = 2024) were included in the meta-analysis. The pooled mean difference favored CSP, with a significantly shorter paced QRS duration (MD: −41.47 ms; 95% CI: −59.40 to − 23.54; *p* < 0.0001). Heterogeneity was substantial (I² = 99.6%, τ² = 916.65), indicating variability across studies. These findings suggest that CSP is associated with more physiological ventricular activation compared with RVP. No asymmetry is present in publication bias (Fig. S43 and S44).

**Fig. 6:**
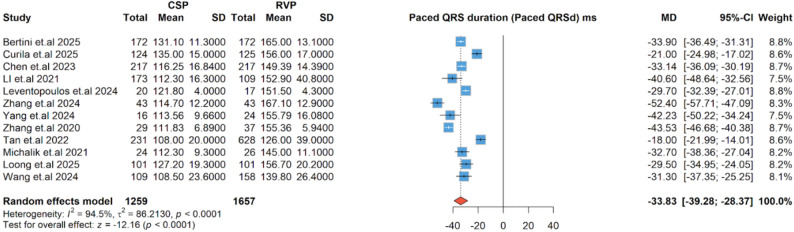
Forest Plot of QRS duration at follow-up

*At Follow-up (ms)*. Thirteen studies comparing CSP and RVP included 1,259 patients in the CSP group and 1,657 in the RVP group. The pooled mean difference in paced QRS duration was − 33.81 ms (95% CI: − 39.27 to − 28.36, *p* < 0.0001), favoring CSP. Heterogeneity was high (I² = 94.5%) (Fig. 6). Sensitivity analysis confirmed the robustness of this effect, with MDs ranging from − 32.10 ms to − 35.26 ms across exclusions, and all p-values remaining < 0.0001. No asymmetry is present in publication bias (Fig. S45 and S46).

**Procedural time (min)**

Five studies (CSP = 483; RVP = 528) showed longer procedural times with CSP than with RVP. The pooled MD was 12.29 min (95% CI: 8.90 to 15.67, *p* < 0.0001), with high heterogeneity (I² = 82.2%). Sensitivity analysis yielded consistent results, with MDs ranging from 11.30 to 13.40 min, and heterogeneity decreased to 31.9% after excluding Wang et al. (2024). These findings likely reflect the greater technical complexity of CSP (Figs. S47 and S48).

**Fluoroscopy time (min)**

Five studies compared fluoroscopy time between CSP and RVP at implantation, including 483 CSP and 527 RVP patients. The pooled mean difference was 4.10 min (95% CI: 2.76 to 5.43, *p* < 0.0001), indicating longer fluoroscopy time with CSP. Heterogeneity was substantial (I² = 91.5%). Leave-one-out analysis showed that omitting Wang et al. (2024) reduced heterogeneity to 36.4%, while the effect size remained significant across all exclusions (MD range: 3.70 to 4.48 min). These findings support greater procedural complexity with CSP (Figs. S49 and S50). A detailed summary of these outcomes is given in Tables 3 and 4.

**Table 3:** Summary of efficacy & safety outcomes with effect estimates and heterogeneity (I²) ²

Outcomes	CSP (I)		RVP (C)		Risk Ratios (RR) (95% CI); (I²); *P*-value	After Sensitivity Analysis	
	Events	Total	Events	Total		I²	RR & *p*-value
Efficacy Outcomes							
Primary Composite Outcomes	181	1928	556	2821	0.44 (0.35–0.55); 42.4%; <0.0001	12.5%	0.42, < 0.0001
All CauseMortality	80	1693	310	2701	0.50 (0.37–0.69); 29.3%; <0.0001	-	-
CV Death	13	1142	35	1667	0.74 (0.36–1.49); 0%; 0.3963	-	-
CRT	0	1188	45	1720	0.0 (0.0-3.72); 0%; 0.1068	-	-
HF Hospitalization	75	1996	309	2889	0.39 (0.31–0.50); 0%; <0.0001	-	-
PICM	39	554	99	971	0.36 (0.14–0.95); 0%; 0.0392	0%	0.21, < 0.0001
Safety Outcomes							
Lead Related Complications	19	1395	32	2118	0.78 (0.42–1.45); 0%; 0.4239	-	-
Pneumothorax	8	963	8	1886	1.77 (0.58–5.38); 0%; 0.3160	-	-
Pericardial Effusion	3	770	11	1420	0.88 (0.18–4.25); 0%; 0.8710	-	-
Pocket Related Complication	4	609	9	1004	1.01 (0.29–3.52); 0%; 0.9852	-	-

**Table 4:** Summary of echocardiographic and procedural outcomes with mean differences and heterogeneity (I²)

Outcomes	Total			Mean Difference (MD) (95% CI); I²; *P*-value	After Sensitivity Analysis	
	CSP		RVP		I²	MD & *p*-value
Echocardiography Outcomes (at final follow-up)						
LVEF %	659	981		2.60 (1.58–3.62); 64.4%; <0.0001	47.6%	2.25, < 0.0001
LAD mm	220	544		-0.14 (-2.65 to 2.37); 85.9%; 0.9128	no change	-
LVEDD mm	432	751		-1.54 (-2.20 to -0.87); 0%; <0.0001	-	-
Procedural /Electrical Outcomes (at Implantation)						
Procedural time (min)	483	528		12.29 (8.90-15.67); 82.2%; <0.0001	31.9%	13.40, < 0.0001
Fluoroscopy time (min)	483	527		4.10 (2.76–5.43); 91.5%; <0.0001	36.4%	4.48, < 0.0001
R-wave amplitude (sense) mV	1283	1870		0.79 (-1.37 to 2.95); 97.1%; 0.4739	95.1%	1.61, 0.0478
Impedance Ohms (Ω)	1042	1627		24.58 (-25.12 to 74.29); 97.2%;0.3324	no change	-
Paced QRS duration (Paced QRSd) ms	1309	2024		-41.47 (-59.40 to -23.54); 99.6%; <0.0001	no change	-
Pacing threshold (V)	1234	1928		0.99 (-0.90 to 2.28); 99.9%; 0.3040	no change	-
At follow-up						
Paced QRS duration (Paced QRSd) ms	1259	1657		-33.81 (-39.27 to -28.36); 94.5%; <0.0001	no change	-
R-wave amplitude (sense) mV	1628	2515		-1.28 (0.87 to 3.42); 99.5%; 0.2426	99.4%	1.91, 0.0479
Impedance Ohms (Ω)	1277	2135		-50.38 (-96.83 to -3.92); 98.4%; 0.0335	95.1%	-60.27, 0.0118

##### Subgroup analysis of secondary outcomes

Upon subgroup analysis of safety outcomes, no significant differences were observed for lead-related complications when stratified by CSP modality (LBBAP, mixed CSP, HBP) or study design compared with RVP. Similar findings were observed for pneumothorax, pericardial effusion, and pocket-related complications (Figs. S67-S74).

For LVEF (%), subgroup analysis by CSP modality showed significant improvement with LBBAP (MD = 2.43, 95% CI 1.40–3.47), while mixed CSP showed no benefit. By study design, cohort studies demonstrated improvement (MD = 2.46, 95% CI 1.50–3.42), while RCTs did not show a significant difference (Figs. S75, S76).

Fluoroscopy time at implantation was significantly longer with LBBAP (MD = 3.08) and mixed CSP (MD = 3.22), while HBP showed no significant difference; these findings were consistent across study designs. Procedural time was also longer with LBBAP (MD = 9.60), mixed CSP (MD = 18.70), and HBP (MD = 59.00), with consistent findings across study designs. In contrast, impedance and lead displacement/dislodgement at implantation and follow-up showed no significant differences between CSP and RVP across modalities or study designs. Paced QRS duration at implantation was significantly shorter with LBBAP (MD = − 45.27 ms), mixed CSP (MD = − 24.07 ms), and HBP (MD = − 35.50 ms) compared with RVP, with similar findings in RCTs. These reductions remained significant at follow-up (LBBAP − 38.09 ms; mixed − 22.60 ms; HBP − 32.70 ms), consistent across study designs. R-wave amplitude was higher with LBBAP (MD = 2.06 mV), lower with HBP (MD = − 5.39 mV), and showed no significant difference with mixed CSP. At follow-up, LBBAP remained higher (MD = 2.73 mV), HBP remained lower (MD = − 5.93 mV), and mixed CSP remained nonsignificant. RCTs showed no significant differences, while cohort studies suggested a nonsignificant trend favoring CSP. Pacing thresholds showed no significant differences when stratified by CSP modality. However, RCTs favored CSP (MD = − 0.15 V), while cohort studies were nonsignificant. At follow-up, LBBAP and HBP remained nonsignificant, and mixed CSP also showed no significant difference (MD = 0.26 V). No significant differences in ventricular pacing rate were observed across CSP modalities or study designs (Figs. S77- S102). Detailed summary of the subgroup analyses are provided in (Supplementary Table S4).

##### Meta-regression

Meta-regression for echocardiographic outcomes was performed to explore the potential influence of follow-up duration on treatment effect estimates for two cardiac outcomes: LVEF and LVEDD.

For LVEF, the regression model showed a negative correlation (*r* = − 0.989, *p* < 0.0001), indicating a decrease in the mean difference in LVEF with longer follow-up, with a downward trend across the 0–3.5-year range. This may suggest that the initial improvement in LVEF associated with CSP could attenuate over time, possibly reflecting disease progression or pacing-related adaptation. The widening confidence intervals at later time points indicate greater uncertainty in long-term estimates (Fig. S51).

For LVEDD, meta-regression demonstrated a mild negative slope, indicating a gradual reduction in LVEDD with increasing follow-up duration. This pattern may be consistent with a potential sustained structural remodeling effect of CSP. The scatter plot showed a generally downward trend in mean LVEDD differences over time, with relatively stable confidence intervals compared with those for LVEF.

Overall, these findings may indicate that while improvements in LVEF could lessen with longer follow-up, changes in LVEDD appear more stable over time, although these observations should be interpreted given the limited number of studies and inherent uncertainty in meta-regression analyses (Fig. S52).

Meta-regression analysis of procedural outcomes was performed based on age, sex, and prior comorbidities including atrial fibrillation, diabetes mellitus, hypertension, and left ventricular ejection fraction (%) to explore potential sources of heterogeneity. For R-wave amplitude at both time points, none of the evaluated covariates significantly explained the observed heterogeneity. Similar findings were demonstrated for procedural time as well, showing no significant associations. For lead displacement or dislodgement at both implantation and follow-up, none of the covariates showed a significant relationship with the evaluated outcome.

However, paced QRS duration (ms) at implantation showed that most covariates did not significantly explain the observed heterogeneity; however, left ventricular ejection fraction was significantly associated with paced QRS duration (β = −0.09, 95% CI − 0.14 to − 0.05, *p* < 0.001), indicating that lower LVEF was associated with longer paced QRS duration. At follow-up, analysis demonstrated that most covariates remained non-significant; but atrial fibrillation was significantly associated with paced QRS duration (β = 0.08, 95% CI 0.01 to 0.14, *p* = 0.02), indicating that AF was linked to longer paced QRS duration. Similarly, left ventricular ejection fraction also remained significant (β = −0.10, 95% CI − 0.16 to − 0.05, *p* < 0.001), consistent with the implantation findings.

For lead impedance (Ω) at both time points, none of the evaluated covariates significantly explained the observed heterogeneity. For fluoroscopy time at implantation, the moderator analysis showed mixed results: age and sex were not significant, while hypertension trended toward significance (*p* = 0.06) but did not reach conventional thresholds. In contrast, atrial fibrillation was significantly associated with shorter fluoroscopy time (β = −0.02, 95% CI − 0.04 to − 0.002, *p* = 0.03), diabetes mellitus was also significant (β = −0.04, 95% CI − 0.07 to − 0.006, *p* = 0.02), and left ventricular ejection fraction showed a strong negative association (β = −0.01, 95% CI − 0.02 to − 0.007, *p* < 0.001). Change in pacing threshold at both implantation and follow-up was non-significant. The detailed summary of meta-regression for procedural outcomes is given in (Supplementary Table S5).

## Discussion

This meta-analysis of 19 studies comparing CSP with conventional RVP in atrioventricular block demonstrates consistent associations favoring CSP across multiple clinical and echocardiographic outcomes. Key findings include a significant reduction in all-cause mortality, fewer heart failure hospitalizations, lower incidence of PICM, and improved composite clinical outcomes. CSP produced narrower paced QRS duration and small but consistent improvements in LVEF and LVEDD, at the cost of longer procedural and fluoroscopy times. Safety endpoints (lead complications, pneumothorax, pericardial effusion, and pocket complications) showed no clear increase in risk with CSP. Meta-regression suggests LVEF benefits attenuate with longer follow-up whereas structural (LVEDD) benefits appear more stable over time.

Multiple recent reviews and meta-analyses have reported physiologic and clinical advantages for CSP over RVP, particularly in terms of ventricular synchrony, LVEF preservation, and reductions in heart failure events; our findings are broadly concordant with this literature and extend it by including a larger pooled sample incorporating contemporary LBBAP and HBP series [31, 32]. A reconstructed individual patient-level meta-analysis presented by Kim et al. and summarized in recent literature similarly reports favorable clinical outcomes with CSP in AV block populations, supporting the plausibility of the associations observed in our study [33].

Our pooled analysis demonstrated an association between CSP and a reduction in all-cause mortality, consistent in direction with contemporary registry and individual-patient analyses [34, 35]. The greater magnitude observed in our study may reflect the inclusion of more recent, larger CSP cohorts and newer randomized data. Prior studies were often underpowered for mortality outcomes; thus, our findings may represent an incremental addition to the existing evidence base rather than a substantial departure from prior evidence [36, 37]. However, studies including broader populations, such as those without LBBB, have not consistently demonstrated similar benefits [38]. Importantly, given the substantial contribution of observational cohorts, residual confounding may influence these estimates; therefore, these findings should be interpreted as associative and hypothesis-generating, requiring confirmation in adequately powered randomized trials.

The observed reduction in HF hospitalizations is consistent with prior observational and pooled analyses reporting fewer HF events with CSP compared with RVP [25, 33]. While earlier meta-analyses showed variable or modest effects [41], more recent larger studies and reconstructed analyses have reported similar trends, particularly in populations with high pacing burden [39–41]. This consistency across studies may support the clinical relevance of the association; however, differences in effect size likely reflect heterogeneity in baseline characteristics, pacing burden, and follow-up duration. Mechanistic plausibility exists given the known benefits of CSP on electrical synchrony and ventricular remodeling, although causal inference remains limited by the observational nature of much of the evidence.

Our pooled reduction in PICM and the near-absence of CRT upgrades among CSP recipients matches mechanistic expectation and multiple cohort studies reporting lower PICM incidence after CSP. Prior syntheses have suggested similar directional benefits, but estimates varied because of small sample sizes and differing PICM definitions [41–43]. The sensitivity of the PICM estimate to individual studies in our analysis echoes earlier literature highlighting susceptibility of PICM results to single influential cohorts; sparse CRT upgrade events likewise limit precision [16, 44, 45]. Overall, our findings reinforce the hypothesis that physiological activation prevents dyssynchrony-mediated remodelling but echo prior calls for standardized PICM definitions and longer follow-up.

The primary composite outcome was defined variably across included studies, with different combinations of clinically meaningful endpoints such as all-cause or cardiovascular mortality, heart failure hospitalization, pacing-induced cardiomyopathy, lead failure, and device upgrades (e.g., CRT or biventricular pacing). A detailed breakdown of study-level composite definitions is provided in Supplementary Table S3. While this variability introduces clinical heterogeneity and limits direct comparability across studies, all included components represent important patient-centered outcomes related to disease progression and pacing-related complications. Importantly, despite these differences in definitions, our pooled analysis demonstrated a consistent and statistically significant reduction in composite events with CSP, with only moderate heterogeneity observed, supporting the overall robustness of the findings.

We observed a modest mean LVEF gain with CSP, consistent with prior meta-analyses and RCTs that reported small but statistically significant improvements versus RVP [16, 35, 46–48]. Earlier reviews often reported heterogeneous LVEF effects and suggested that benefits are greatest in subgroups with baseline LV dysfunction or high pacing burden ([49–50]). Our meta-regression showing attenuation of LVEF effect over time provides an explanatory nuance that has been suggested but not uniformly demonstrated in prior work. In sum, our LVEF findings concur with the literature: functional gains are modest at the group level, clinically relevant in select patients, and may diminish with longer follow-up.

The consistent LVEDD reduction we report ( ≈ − 1.5 mm) is concordant with prior data showing structural reverse remodeling after CSP; several earlier analyses emphasized LVEDD or LV volumes as more consistent remodeling markers than LVEF [46, 47]. Our finding of persistent LVEDD benefit despite attenuation of LVEF over time mirrors mechanistic and imaging studies in the literature proposing more durable anatomic remodeling than immediate contractile improvement. LAD effects remain inconsistent across reports, including ours, reflecting limited data and between-study variability. Meta-regression suggested a time-related trend in LVEDD reduction, although these observations are exploratory and may be influenced by study-level variability and limited adjustment for individual patient characteristics.

The substantial narrowing of paced QRS with CSP is a robust and reproducible finding across virtually all prior studies and meta-analyses; our pooled estimate of roughly a 30–40 ms shortening is fully aligned with the literature and underpins the mechanistic rationale for improved outcomes ([47, 49–50]). Prior work uniformly identifies paced QRS as a mediator of dyssynchrony and adverse remodeling; our results consolidate that electrical synchrony advantage as one of the most consistent differences between CSP and RVP.

Consistent with prior literature, we found no consistent or clinically meaningful differences in pacing thresholds, with only inconsistent findings for R-wave amplitude and impedance and very high between-study heterogeneity. Previous reviews similarly report mixed results, likely attributable to differences in lead types, definitions (His-bundle vs. LBBAP), device programming, and measurement timing. Our findings therefore support the prevailing view that modern CSP techniques can achieve acceptable electrical parameters, although device- and operator-specific factors likely account for much of the observed variability.

Our finding of longer procedural (~ 12 min) and fluoroscopy (~ 4 min) times with CSP is consistent with prior reports such as Felix et al. [50] where no statistically significant difference in procedure time was noted. However, in a study conducted by Peng et al. reduction in the procedure and fluoroscopy time was observed [41]. Several prior studies and expert commentaries note that procedural time and radiation exposure decline with operator experience and standardized workflows [51, 52]. 

Meta-regression was conducted for procedural outcomes to explore potential sources of heterogeneity. Covariates including baseline demographics and clinical characteristics, such as age, sex, atrial fibrillation, diabetes mellitus, hypertension, and left ventricular ejection fraction (LVEF) were evaluated. Overall, these factors did not explain between-study variability for most procedural outcomes, suggesting that heterogeneity may be driven by other factors such as operator experience and learning curve, variations in implantation techniques, device and lead types, procedural protocols, institutional practices, study design, patient selection, and outcome measurement methods. However, for paced QRS duration, LVEF was a significant moderator at both implantation and follow-up, while atrial fibrillation was additionally significant at follow-up, indicating a potential influence of cardiac function and rhythm status on electrical remodeling. Similarly, for fluoroscopy time, atrial fibrillation, diabetes mellitus, and LVEF showed significant associations, whereas other covariates remained non-significant. Overall, while patient-level factors may influence selected outcomes, most variability appears to be driven by procedural and system-level differences. Therefore, findings of our analysis for procedural outcomes should be considered suggestive rather than definitive, underscoring the need for high-quality studies with standardized protocols, uniform techniques, and consistent outcome reporting to better define true effect estimates and reduce heterogeneity.

Like many earlier syntheses, we found no clear increase in common complication rates with CSP but wide confidence intervals for rare events and sparse data for some endpoints. Earlier registry and cohort studies have reported similar safety profiles but warned that technique-specific complications (e.g., lead dislodgement, capture loss in His-bundle pacing) require continued surveillance [53, 54]. Our results therefore support prior conclusions: CSP appears safe in experienced hands, but larger, longer follow-up studies are necessary to rule out infrequent but important adverse events.

Upon subgroup analyses on the basis of CSP modality and study design, the findings reinforce the clinical relevance of CSP. When stratified by intervention type, particularly LBBAP showed marked improvement in hard outcomes. The consistent reductions observed in all-cause mortality, heart failure hospitalization, CRT upgrade, and PICM with LBBAP, and to a similar extent in mixed CSP (LBBAP + HBP), are more likely to reflect the heart’s natural ventricular activation pattern and help reduce dyssynchrony compared with RVP. However, the lack of significance with HBP may be attributed to limited sample size and technical challenges associated with lead placement and stability, rather than true inferiority. Notably, the consistency between cohort studies, and in several outcomes, RCTs, supports the robustness and generalizability of these findings, although some discrepancies (e.g., mortality and PICM) may reflect potential underpowering of randomized data. Improvements in LVEF and reduction in paced QRS duration further support the mechanistic basis of benefit with CSP. However, along with these advantages, our analysis suggests that CSP is associated with longer procedural and fluoroscopy times, indicating a balance between procedural complexity and long-term clinical benefit. The absence of differences in safety outcomes and lead-related complications suggests that CSP can be used without compromising procedural safety. Overall, these subgroup findings support a shift toward CSP, particularly LBBAP, as a preferred pacing strategy in appropriate patients, while also emphasizing the need for further high-quality randomized trials and additional research on HBP.

A recurrent theme in the literature is that His-bundle pacing and left bundle branch area pacing are not identical interventions; many prior analyses have highlighted important differences in success rates, thresholds, sensing, and durability [43, 46, 54, 55]. Our pooled approach combining LBBAP, LBBP and HBP studies replicates the heterogeneity reported previously and reinforces calls in the literature for trials and meta-analyses stratified by CSP subtype. Where prior head-to-head comparisons exist, LBBAP often shows higher success and more stable thresholds than classical His-bundle pacing; our dataset contains both modalities and the mixed evidence reflects those technique-specific trade-offs.

### Strength and limitations

This systematic review and meta-analysis leverages data from 19 studies encompassing randomized controlled trials, prospective and retrospective cohorts, and registry datasets, thereby combining the internal validity of randomized evidence with the external validity of real-world practice. Unlike earlier reviews, which primarily focused on clinical outcomes, our analysis integrates echocardiographic and electrical parameters, offering a broader perspective on physiologic pacing. The inclusion of recent randomized data and large LBBAP cohorts strengthens the generalizability of our findings, though the predominance of observational evidence underscores the need for cautious interpretation. The analysis shows consistent patterns across clinically meaningful endpoints, including all-cause mortality and heart failure hospitalizations, alongside objective electrophysiologic and echocardiographic measures (paced QRS duration, LVEDD, LVEF), which together support biological plausibility and reduce concern that the observed clinical associations are purely artefactual. Methodological strengths include preplanned sensitivity and leave-one-out analyses for most outcomes and meta-regression to explore time-dependent effects.

Nevertheless, several limitations temper the certainty of the findings. Heterogeneity in study design, lead technology, and operator experience introduces clinical and statistical heterogeneity that complicates pooled estimates for certain endpoints, particularly electrical parameters and impedance. For outcomes demonstrating extreme heterogeneity (I² >90%), particularly electrical parameters such as impedance, R-wave amplitude, and pacing thresholds, pooled estimates should be interpreted as exploratory rather than definitive, as such variability limits the reliability of a single summary effect size. Some asymmetry was observed in funnel plots for HF hospitalization and certain electrical outcomes, which may reflect small-study effects, clinical heterogeneity, or reporting variability; therefore, these findings should be interpreted with appropriate caution. A substantial proportion of data derive from observational sources and registries, which are susceptible to selection bias, unmeasured confounding, and variable outcome adjudication. Event rates for some safety and upgrade outcomes were sparse, yielding wide confidence intervals and limited statistical power. Future studies reporting HBP and LBBAP outcomes separately are needed to allow clearer stratification and better understanding of modality-specific effects. Finally, median follow-up was frequently short to intermediate, and meta--regression evidence of attenuation in LVEF benefit over time underscores uncertainty regarding long-term functional durability.

### Practical implications

From a clinical and systems perspective, the aggregated evidence supports prioritization of conduction system pacing in patients with anticipated high cumulative ventricular pacing burden and in those with marginal left ventricular function in whom avoidance of chronic dyssynchrony is a therapeutic objective [56]. Implementation at the centre level should be deliberate and programmatic: structured operator training, proctoring, and adoption of standardized implantation and capture verification protocols are essential to shorten the learning curve and reduce procedural and fluoroscopic exposure [57, 58]. Device follow-up pathways should incorporate routine echocardiographic surveillance, systematic device interrogation, and threshold management to detect pacing-induced remodeling early and to guide timely intervention [59]. Health-service planners should account for upstream resource implications, longer procedure and fluoroscopy times and initial training costs, against downstream reductions in heart-failure admissions and mortality; prospective collection of local outcome, utilization, and cost data will be necessary to inform rigorous cost-effectiveness and budget-impact assessments. In shared decision-making, clinicians should present CSP as a strategy with evolving evidence for improved clinical outcomes but also describe the current limitations in long-term data and the procedural trade-offs.

### Mechanistic considerations

The mechanistic rationale for the observed clinical and structural benefits of conduction system pacing rests on preservation of near-physiological electrical activation and consequent mitigation of mechanical dyssynchrony. Narrower paced QRS complexes with CSP are indicative of more synchronous left and right ventricular depolarization, which reduces regional wall stress and abnormal interventricular timing that otherwise promote adverse remodeling [59, 60]. These electrophysiologic advantages plausibly mediate the observed reductions in LV end-diastolic diameter and lower incidence of pacing-induced cardiomyopathy, and they provide a mechanistic link to reductions in heart-failure hospitalizations and mortality. The differing temporal patterns observed in echocardiographic outcomes, namely sustained improvement in LVEDD with attenuation of LVEF gains over time, suggest that structural reverse remodeling may be more durable than improvements in systolic function, which may be influenced by progressive myocardial disease, comorbidities, and long-term pacing-related adaptation [61]. Further mechanistic work incorporating multimodal imaging, myocardial strain analysis, and biomarker profiling will be necessary to delineate the pathways by which CSP confers sustained clinical benefit and to identify patient subgroups most likely to derive durable functional improvement [62].

## Conclusion

The synthesized evidence suggests that conduction system pacing is associated with a lower risk of all-cause mortality compared with right ventricular pacing; however, given the predominance of observational data, this finding should be considered as associative and hypothesis-generating rather than causal. Beyond mortality, CSP was associated with improved clinical and physiological outcomes, including reductions in heart failure hospitalization and pacing-induced cardiomyopathy, as well as improved ventricular synchrony and structural remodeling. While CSP may represent a physiologically favorable pacing strategy, its use should be individualized and supported by appropriate operator expertise and structured follow-up. This also highlights the need for large, long-term randomized trials with standardized outcome reporting to determine whether CSP confers definitive long-term benefits compared with RVP.

## Supplementary Information

Supplementary Material 1.

## Data Availability

All data generated or analyzed during this study are included in this published article and its supplementary information files.

## Abbreviations

AV: Atrioventricular
BiVP: Biventricular Pacing
CI: Confidence Interval
CRT: Cardiac Resynchronization Therapy
CRTD: Cardiac Resynchronization Therapy Defibrillator
CSP: Conduction System Pacing
CV: Cardiovascular
DM: Diabetes Mellitus
ECG: Electrocardiogram
EF: Ejection Fraction
HBP: His Bundle Pacing
HF: Heart Failure
HR: Hazard Ratio
HTN: Hypertension
I²: I-squared (measure of heterogeneity)
LAD: Left Atrial Dimension
LBBAP: Left Bundle Branch Area Pacing
LBBP: Left Bundle Branch Pacing
LBBB: Left Bundle Branch Block
LV: Left Ventricle
LVEDD: Left Ventricular End-Diastolic Diameter
LVEF: Left Ventricular Ejection Fraction
MD: Mean Difference
NOS: Newcastle–Ottawa Scale
PICM: Pacing-Induced Cardiomyopathy
PRISMA: Preferred Reporting Items for Systematic Reviews and Meta-Analyses
PROSPERO: International Prospective Register of Systematic Reviews
RCT: Randomized Controlled Trial
ROB: Risk of Bias
ROB2: Risk of Bias 2 Tool
RR: Risk Ratio
RVP: Right Ventricular Pacing
τ²: Tau-squared (between-study variance)
