# Supplementary material for: Clinical and electrical outcomes of conduction system pacing versus right ventricular pacing in atrioventricular block: a systematic review and meta-analysis
Source: BMC Cardiovasc Disord. 2026 May 6;26:542. doi: 10.1186/s12872-026-05930-6 (PMC13317157; doi:10.1186/s12872-026-05930-6)
Supplement: Supplementary file 1 — Supplementary Material 1. [file 12872_2026_5930_MOESM1_ESM.docx]

| **Databases** | **Keywords** | **Results** |
| --- | --- | --- |
| Pubmed | ( ( "Atrioventricular Block"[Mesh] OR "Heart Block"[Mesh] OR "atrioventricular block"[Title/Abstract] OR "AV block"[Title/Abstract] OR "heart block"[Title/Abstract] OR "complete heart block"[Title/Abstract] OR "complete AV block"[Title/Abstract] OR "high-grade AV block"[Title/Abstract] OR "advanced AV block"[Title/Abstract] OR "third-degree AV block"[Title/Abstract] OR "third degree AV block"[Title/Abstract] ) AND ( "Cardiac Pacing, Artificial"[Mesh] OR "conduction system pacing"[Title/Abstract] OR "conduction-system pacing"[Title/Abstract] OR "His bundle pacing"[Title/Abstract] OR "His-bundle pacing"[Title/Abstract] OR HBP[Title/Abstract] OR "selective His pacing"[Title/Abstract] OR "nonselective His pacing"[Title/Abstract] OR "His-Purkinje pacing"[Title/Abstract] OR "His-Purkinje conduction pacing"[Title/Abstract] OR "left bundle branch pacing"[Title/Abstract] OR "left bundle branch area pacing"[Title/Abstract] OR "left bundle branch area capture"[Title/Abstract] OR LBBP[Title/Abstract] OR LBBAP[Title/Abstract] OR "LBB area pacing"[Title/Abstract] OR "deep septal pacing"[Title/Abstract] OR "left ventricular septal pacing"[Title/Abstract] OR LVSP[Title/Abstract] OR "His-Purkinje capture"[Title/Abstract] OR "left bundle branch capture"[Title/Abstract] OR "physiologic pacing"[Title/Abstract] OR "physiological pacing"[Title/Abstract] ) AND ( "right ventricular pacing"[Title/Abstract] OR "RV pacing"[Title/Abstract] OR "right ventricle pacing"[Title/Abstract] OR "ventricular pacing"[Title/Abstract] OR "apical pacing"[Title/Abstract] OR "right ventricular apical pacing"[Title/Abstract] OR RVAP[Title/Abstract] OR "septal pacing"[Title/Abstract] OR "right ventricular septal pacing"[Title/Abstract] OR RVSP[Title/Abstract] OR "right ventricular outflow tract pacing"[Title/Abstract] OR "RVOT pacing"[Title/Abstract] ) ) | 1421 |
| Embase | ( 'atrioventricular block'/exp OR 'atrioventricular block' OR 'AV block' OR 'complete heart block' OR 'complete AV block' OR 'high-grade AV block' OR 'advanced AV block' OR 'third-degree AV block' ) AND ( 'conduction system pacing' OR 'His bundle pacing' OR 'His-bundle pacing' OR HBP OR 'left bundle branch pacing' OR 'left bundle branch area pacing' OR LBBP OR LBBAP OR 'physiological pacing' ) AND ( 'right ventricular pacing' OR RVP OR 'apical pacing' OR 'right ventricular apical pacing' OR RVAP OR 'septal pacing' OR 'right ventricular septal pacing' OR RVSP ) | 390 |
| Sciencedirect | ("atrioventricular block" OR "AV block")AND("conduction system pacing" OR "His bundle pacing" OR "left bundle branch pacing")AND("right ventricular pacing" OR RVP) | 509 |
| Cochrane | #1 MeSH descriptor: [Atrioventricular Block] explode all trees #2 (atrioventricular block):ti,ab,kw #3 (AV block):ti,ab,kw #4 MeSH descriptor: [Heart Block] explode all trees #5 (heart block):ti,ab,kw #6 (complete heart block):ti,ab,kw #7 (complete AV block):ti,ab,kw #8 (high-grade AV block):ti,ab,kw #9 (advanced AV block):ti,ab,kw #10 (third-degree AV block):ti,ab,kw #11 (third degree AV block):ti,ab,kw  #12 #1 OR #2 OR #3 OR #4 OR #5 OR #6 OR #7 OR #8 OR #9 OR #10 OR #11  #13 (conduction system pacing):ti,ab,kw #14 (conduction-system pacing):ti,ab,kw #15 (His bundle pacing):ti,ab,kw #16 (His-bundle pacing):ti,ab,kw #17 (HBP):ti,ab,kw #18 (selective His pacing):ti,ab,kw #19 (nonselective His pacing):ti,ab,kw #20 (His-Purkinje pacing):ti,ab,kw #21 (His-Purkinje conduction pacing):ti,ab,kw #22 (left bundle branch pacing):ti,ab,kw #23 (left bundle branch area pacing):ti,ab,kw #24 (LBB area pacing):ti,ab,kw #25 (deep septal pacing):ti,ab,kw #26 (left ventricular septal pacing):ti,ab,kw #27 (LVSP):ti,ab,kw #28 (physiologic pacing):ti,ab,kw  #29 #13 OR #14 OR #15 OR #16 OR #17 OR #18 OR #19 OR #20 OR #21 OR #22 OR #23 OR #24 OR #25 OR #26 OR #27 OR #28  #30 (right ventricular pacing):ti,ab,kw #31 (RV pacing):ti,ab,kw #32 (right ventricle pacing):ti,ab,kw #33 (ventricular pacing):ti,ab,kw  #34 (apical pacing):ti,ab,kw  #35 (right ventricular apical pacing):ti,ab,kw  #36 (RVAP):ti,ab,kw  #37 (right ventricular septal pacing):ti,ab,kw  #38 (septal pacing):ti,ab,kw  #39 (right ventricular outflow tract pacing):ti,ab,kw  #40 (RVOT pacing):ti,ab,kw  #41 #30 OR #31 OR #32 OR #33 OR #34 OR #35 OR #36 OR #37 OR #39 OR #40  #42 #12 AND #29 AND #41 | 368 |

**Supplementary Table 1:** Search Strategy Table

|  | Selection (4) | Comparability (2) | Outcome (3) | Total NOS Score (0-9) | Overall risk of bias |
| --- | --- | --- | --- | --- | --- |
| Chen et.al. 2023 | ★★★★ | ★★ | ★★★ | ★★★★★★★★★ | low |
| Inoue et.al. 2025 | ★★★ | ★ | ★★★ | ★★★★★★★ | moderate |
| Kono et.al. 2024 | ★★★ | ★★ | ★★★ | ★★★★★★★★ | low |
| Leventoupolous et.al. 2024 | ★★★ | ★★ | ★★★ | ★★★★★★★★ | low |
| Li et.al. 2021 | ★★★★ | ★★ | ★★★ | ★★★★★★★★★ | low |
| Michalik et. al. 2021 | ★★★★ | ★★ | ★★★ | ★★★★★★★★★ | low |
| Okubo et al. 2024 | ★★★★ | ★★ | ★★★ | ★★★★★★★★★ | low |
| Shimeno et. al. 2025 | ★★★★ | ★★ | ★★★ | ★★★★★★★★★ | low |
| Tan et. al. 2022 | ★★★ | ★★ | ★★★ | ★★★★★★★★ | low |
| Wang et.al. 2024 | ★★★★ | ★★ | ★★★ | ★★★★★★★★★ | low |
| Yang et.al.2024 | ★★★★ | ★★ | ★★★ | ★★★★★★★★★ | low |
| Yeon et. al. 2025 | ★★★ | ★★ | ★★★ | ★★★★★★★★ | low |
| Chen.et. al. 2024 | ★★★★ | ★★ | ★★★ | ★★★★★★★★★ | low |
| Zhang et. al. 2024 | ★★★★ | ★★ | ★★★ | ★★★★★★★★★ | low |
| Zhang et. al. 2020 | ★★★★ | ★★ | ★★★ | ★★★★★★★★★ | low |

**Supplementary Table S2:** Quality Assessment of Included Studies Using the Newcastle–Ottawa Scale (NOS)


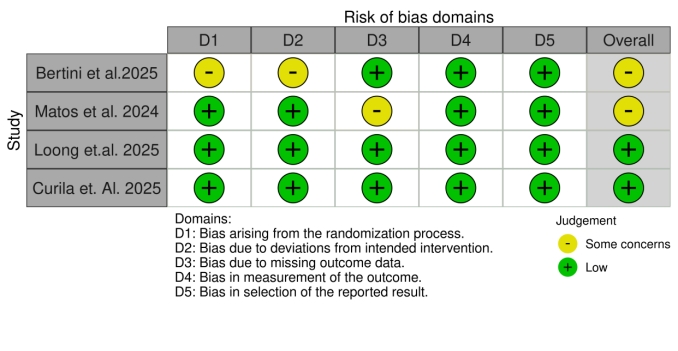


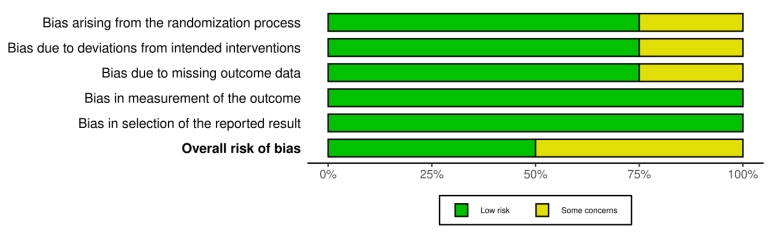


**Supplementary Figure S1:** Quality Assessment of Included Studies Using the Cochrane Risk of Bias 2 tool.

| **Study author & year** | **Primary Composite Outcomes** |
| --- | --- |
| **Bertini**  **et al. 2025** | Cardiovascular death, heart failure hospitalization |
| **Curila et.al 2025** | Left ventricular ejection fraction decline |
| **Chen**  **et al. 2023** | All-cause mortality, lead failure, heart failure |
| **Li et al. 2021** | Heart failure hospitalization, upgrade to biventricular pacing |
| **Kono et al. 2024** | Heart failure hospitalization |
| **Yeon et al. 2025** | Heart failure hospitalization, pacing-induced cardiomyopathy, upgrade to biventricular pacing, all-cause mortality |
| **Tan et.al 2022** | Heart failure hospitalization, upgrade to biventricular pacing, all-cause mortality |
| **Loong et.al 2025** | Pacing-induced cardiomyopathy, upgrade to biventricular pacing, heart failure hospitalization, all-cause mortality |
| **Okubo et al. 2024** | All-cause mortality, heart failure hospitalization, upgrade to biventricular pacing |
| **Wang et al. 2024** | All-cause mortality, heart failure hospitalization, upgrade to biventricular pacing |
| **Shimeno et al. 2025** | Pacing-induced cardiomyopathy |

**Supplementary Table S3:** Summary of primary composite outcomes

| Outcomes | Intervention based | | | | | | | Study Design | | | | |
| --- | --- | --- | --- | --- | --- | --- | --- | --- | --- | --- | --- | --- |
|  | **LBBAP** | | **Mixed CSP** | | **HBP** | | **Subgroup difference** | **RCT** | | **Cohort** | | **Subgroup difference** |
|  | RR/MD [95%CI] | Heterogeneity (I², p) | RR/MD [95%CI] | Heterogeneity (I², p) | RR/MD [95%CI] | Heterogeneity (I², p) | P-value | RR/MD [95%CI] | Heterogeneity (I², p) | RR/MD (95%CI) | Heterogeneity (I², p) | P-value |
| Efficacy Outcomes | | | | | | | | | | | |  |
| Primary Composite Outcomes | RR: 0.41 [0.28–0.60] | I² = 60.3%, p = 0.0138 | RR: 0.41 [0.31–0.55] | I² = 0%, p = 0.9649 | RR” 0.76 [0.39–1.48] | - | 0.2292 | RR: 0.43 [0.29–0.65] | I² = 0%, p = 0.8546 | RR: 0.43 [0.31–0.59] | I² = 58.8%, p = 0.0175 | 0.9745 |
| All Cause Mortality | RR: 0.48 [0.31–0.75] | I² = 37.9%, p = 0.1393 | RR: 0.44 [0.29–0.66] | I² = 0%, p = 0.7400 | RR: 1.12 [0.46–2.73] | - | 0.1699 | RR: 0.67 [0.38–1.20] | I² = 0%, p = 0.8809 | RR: 0.46 [0.31–0.68] | I² = 44.8%, p = 0.0928 | 0.2808 |
| CV Death | RR: 0.55 [0.25–1.18] | I² = 0%, p = 0.7533 | RR: 1.01 [0.26–3.94] | - | RR: 0.28 [0.02–5.00] | - | 0.6434 | RR: 0.90 [0.37–2.19] | I² = 0%, p = 0.8354 | RR: 0.37 [0.14–1.03] | I² = 0%, p = 0.6904 | 0.1991 |
| CRT | RR: 0.10 [0.02–0.42] | I² = 0%, p = 0.9714 | RR: 0.10 [0.02–0.54] | I² = 0%, p = 0.9384 | - | - | 0.9782 | RR: 0.11 [0.02–0.61] | I² = 0%, p = 0.9764 | RR: 0.09 [0.02–0.39] | I² = 0%, p = 0.9627 | 0.8587 |
| HF Hospitalization | RR: 0.41 [0.30–0.56] | I² = 11.4%, p = 0.3397 | RR: 0.38 [0.24–0.59] | I² = 0%, p = 0.6957 | RR: 0.50 [0.18–1.37] | - | 0.8913 | RR: 0.30 [0.15–0.58] | I² = 0%, p = 0.8814 | RR: 0.41 [0.31–0.54] | I² = 0%, p = 0.4568 | 0.3827 |
| PICM | RR: 0.21 [0.10–0.45] | I² = 0%, p = 0.8670 | RR: 0.37 [0.05–2.93] | - | RR: 1.00 [0.66–1.51] | - | 0.0016 | RR: 1.00 [0.66–1.51] | I² = 77.9%, p = 0.0036 | RR: 0.21 [0.10–0.43] | I² = 0%, p = 0.8871 | 0.0003 |
| Safety Outcomes | | | | | | | | | | | |  |
| Lead Related Complications | RR: 0.93 [0.45–1.91] | I² = 0%, p = 0.7466 | RR: 0.47 [0.17–1.31] | I² = 0%, p = 0.6726 | RR:(no events) | - | 0.2833 | RR: 0.41 [0.13–1.33] | I² = 0%, p = 0.4856 | RR: 0.90 [0.46–1.79] | I² = 0%, p = 0.8317 | 0.2590 |
| Pneumothorax | RR: 1.66 [0.37–7.42] | I² = 0%, p = 0.4441 | RR: 1.99 [0.57–6.88] | I² = 10.2%, p = 0.3283 | RR:(no events) | - | 0.8588 | RR: 0.43 [0.06–2.94] | I² = 0%, p = 0.8451 | RR: 2.99 [0.99–9.02] | I² = 0%, p = 0.8240 | 0.0865 |
| Pericardial Effusion | RR: 0.27 [0.03–2.33] | I² = 0%, p = 0.8604 | RR: 1.15 [0.28–4.79] | I² = 0%, p = 0.5078 | RR:(no events) | - | 0.2705 | RR: 1.00 [0.11–9.60] | I² = 0%, p = 0.3384 | RR: 0.66 [0.16–2.67] | I² = 0%, p = 0.4131 | 0.7553 |
| Pocket Related Complication | RR: 0.73 [0.14–3.87] | I² = 0%, p = 0.5512 | RR: 0.85 [0.20–3.65] | I² = 0%, p = 0.5208 | RR:(no events) | - | 0.8946 | RR: 0.74 [0.14–3.92] | I² = 0%, p = 0.5675 | RR: 0.84 [0.20–3.61] | I² = 0%, p = 0.5043 | 0.9107 |
| Echocardiography Outcomes (at final follow-up) | | | | | | | | | | | |  |
| LVEF % | MD: 1.28 [−0.10–2.66] | I² = 0%, p = 0.3288 | MD: 1.28 [−0.10–2.66] | I² = 0%, p = 0.3288 | - | - | 0.1899 | MD: 1.00 [-0.49, 2.49] | - | MD: 2.46 [1.50, 3.42] | I² = 42.7%, p = 0.0939 | 0.1070 |
| Procedural /Electrical Outcomes (at Implantation) | | | | | | | | | | | |  |
| Procedural time (min) | MD: 9.60 [5.16–14.04] | I² = 94.6%, p < 0.0001 | MD: 18.70 [11.25–26.15] | - | MD: 59.00 [53.69–64.31] | - | 0.001 | MD: 14.98 [10.54–19.41] | I² = 35.1%, p = 0.2145 | MD: 26.97 [−3.96–57.90] | I² = 99.8%, p < 0.0001 | 0.4517 |
| Fluoroscopy time (min) | MD: 3.08 [1.48–4.67] | I² = 96.4%, p < 0.0001 | MD: 6.10 [4.22–7.98] | - | MD: 1.20 [−0.62–3.02] | - | 0.0011 | MD: 4.97 [3.27–6.68] | I² = 68.4%, p = 0.0754 | MD: 6.02 [1.10–10.94] | I² = 98.9%, p < 0.0001 | 0.6932 |
| R-wave amplitude (sense) mV | MD: 2.06 [0.53–3.58] | I² = 92%, p < 0.0001 | MD: −1.80 [−2.60–−1.00] | - | MD: −5.39 [−7.80–−2.97] | I² = 92.2%, p = 0.0004 | 0.001 | MD: −0.40 [−2.10–1.30] | - | MD: 1.34 [−1.30–3.97] | I² = 98.6%, p < 0.0001 | 0.2778 |
| Impedance Ohms (Ω) | MD: 25.93 [−31.45–83.32] | I² = 97.1%, p < 0.0001 | MD: 12.00 [−19.39–43.39] | - | MD: −117.95 [−143.65–−92.25] | - | 0.001 | MD: 20.00 [−4.33–44.33] | - | MD: 101.08 [−96.88–299.04] | I² = 99.8%, p = 0 | 0.4256 |
| Paced QRS duration (Paced QRSd) ms | MD: −45.27 [−66.51–−24.03] | I² = 99.7%, p = 0 | MD: −24.07 [−30.58–−17.55] | I² = 73.4%, p = 0.0527 | MD: −35.50 [−40.73–−30.27] | - | 0.0119 | MD: −27.40 [−39.74–−15.05] | I² = 96.4%, p < 0.0001 | MD: −31.06 [−66.18–4.07] | I² = 99.9%, p = 0 | 0.8473 |
| Pacing threshold (V) | MD: −0.03 [−0.09–0.02] | I² = 86.7%, p < 0.0001 | MD: 3.26 [−3.03–9.55] | I² = 100%, p = 0 | MD: 0.40 [0.31–0.49] | - | 0.001 | MD = −0.15 [−0.25; −0.05] | I² = 82%, p = 0.0185 | MD = 1.13 [−0.96; 3.23] | I² = 99.9%, p = 0 | 0.229 |
| lead displacement/dislodgement | RR: 0.67 [0.11–3.94] | - | RR: 1.16 [0.05–28.24] | - | RR:(no events) | - | 0.7670 | RR: 0.67 [0.11–3.94] | - | RR: 1.16 [0.05–28.24] | - | 0.7670 |
| Procedural /Electrical Outcomes (at Follow-up) | | | | | | | | | | | |  |
| Paced QRS duration (Paced QRSd) ms | MD: −38.09 [−43.54–−32.65] | I² = 92.3%, p < 0.0001 | MD: −22.60 [−29.14–−16.07] | I² = 82.3%, p = 0.0035 | MD: −32.70 [−38.36–−27.04] | - | 0.0016 | MD: −28.21 [−35.83–−20.59] | I² = 93%, p < 0.0001 | MD: −35.77 [−42.37–−29.17] | I² = 95%, p < 0.0001 | 0.1417 |
| Pacing threshold (V) | MD: **-0.02** [ -0.08; 0.05] | I² = 90.9%, p < 0.0001 | MD: 0.26 [ -0.13; 0.65] | I² = 97.1%, p < 0.0001 | MDl 0.65 [ -0.51; 0.79] | - | 0.001 | MD = 0.12 [ -0.32; 0.57] | I² = 98%, p < 0.0001 | MD = 0.07 [ -0.06; 0.20] | I² = 93.9%, p < 0.0001 | 0.8280 |
| R-wave amplitude (sense) mV | MD: 2.73 [0.98–4.49] | I² = 99.4%, p = 0 | MD: −2.62 [−7.03–1.79] | I² = 96.7%, p < 0.0001 | MD: −5.93 [−7.83–−4.03] | I² = 87.4%, p = 0.0048 | 0.001 | MD: −2.61 [−7.02–1.80] | I² = 97.4%, p < 0.0001 | MD: 2.34 [−0.44–5.11] | I² = 99.4%, p = 0 | 0.0628 |
| Impedance Ohms (Ω) | MD: −45.93 [−103.22–11.36] | I² = 98.1%, p < 0.0001 | MD: −71.69 [−84.38–−59.01] | I² = 0%, p = 0.7344 | MD: −75.00 [−91.04–−58.96] | - | 0.6278 | MD: −21.54 [−112.68–69.60] | I² = 96.2%, p < 0.0001 | MD: −10.65 [−137.07–115.77] | I² = 99.9%, p = 0 | 0.8911 |
| Lead displacement/dislodgement | RR: 0.60 [0.20–1.79] | I² = 21.2%, p = 0.2744 | RR: 1.36 [0.27–6.90] | I² = 34.4%, p = 0.2177 | RR:(no events) | - | 0.4130 | RR: 3.21 [0.31–33.33] | I² = 27.4%, p = 0.2405 | RR: 0.65 [0.28–1.50] | I² = 6.2%, p = 0.3802 | 0.2084 |
| Ventricular pacing rate | MD = 15.48 [ −9.55; 40.52] | I² = 99.6%, p < 0.0001 | MD = 0.21 [ −1.94; 2.36] | I² = 0%, p = 0.6943 | MD = 22.80 [ 3.99; 41.61] | I² = 99.3%, p < 0.0001 | 0.0328 | MD = 0.21 [ −1.94; 2.36] | I² = 0%, p = 0.6943 | MD = 29.19 [ −22.84; 81.22] | I² = 99%, p < 0.0001 | 0.2754 |

**Supplementary Table S4:** Summary of Subgroup Analyses

| Procedural Outcomes | Age | | | Male | | | Atrial Fibrillation (AF) | | | Hypertension  (HTN) | | | Diabetes Mellitus (DM) | | | Left Ventricular Ejection Fraction (LVEF)% | | |
| --- | --- | --- | --- | --- | --- | --- | --- | --- | --- | --- | --- | --- | --- | --- | --- | --- | --- | --- |
|  | \|  \| \| --- \|   **β coefficient** | **95% CI (ci.lb to ci.ub)** | **p-value** | \|  \| \| --- \|   **β coefficient** | **95% CI (ci.lb to ci.ub)** | **p-value** | \|  \| \| --- \|   **β coefficient** | **95% CI (ci.lb to ci.ub)** | **p-value** | \|  \| \| --- \|   **β coefficient** | **95% CI (ci.lb to ci.ub)** | **p-value** | \|  \| \| --- \|   **β coefficient** | **95% CI (ci.lb to ci.ub)** | **p-value** | \|  \| \| --- \|   **β coefficient** | **95% CI (ci.lb to ci.ub)** | **p-value** |
| R-wave amplitude (sense) mV at Implantation | −0.02 | (−0.18, 0.13) | 0.76 | −0.0006 | (−0.02, 0.02) | 0.95 | 0.00006 | (−0.07, 0.07) | 0.99 | 0.00002 | \|  \| \| --- \|  \| (−0.01, 0.01) \| \| --- \| | 0.99 | \|  \| \| --- \|  \| −0.0004 \| \| --- \| | (−0.03, 0.03) | \|  \| \| --- \|  \| 0.97 \| \| --- \| | −0.02 | \|  \| \| --- \|  \| (−0.07, 0.02) \| \| --- \| | \|  \| \| --- \|  \| 0.30 \| \| --- \| |
| R-wave amplitude (sense) mV at follow-up | 0.019 | (−0.183, 0.221) | 0.855 | 0.008 | (−0.020, 0.035) | 0.586 | 0.044 | (−0.041, 0.128) | 0.311 | 0.004 | (−0.013, 0.022) | 0.640 | 0.005 | (−0.032, 0.042) | \|  \| 0.788 \| \| --- \| --- \| | 0.027 | (−0.037, 0.091) | 0.410 |
| Procedural Time at Implantation | 0.052 | (−0.163, 0.267) | 0.634 | 0.039 | (−0.028, 0.106) | 0.250 | 0.061 | (−0.064, 0.187) | 0.339 | 0.031 | (−0.017, 0.078) | 0.206 | 0.096 | (−0.118, 0.310) | 0.378 | 0.039 | (−0.021, 0.100) | 0.203 |
| Pacing Threshold at Implantation | 0.026 | (−0.051, 0.102) | 0.507 | 0.001 | (−0.009, 0.011) | 0.851 | −0.005 | (−0.046, 0.036) | 0.811 | 0.001 | (−0.005, 0.007) | 0.688 | 0.002 | (−0.011, 0.015) | 0.723 | −0.0002 | (−0.025, 0.025) | 0.985 |
| Pacing Threshold at Follow-up | 0.025 | \|  \| (−0.040, 0.090) \| \| --- \| --- \| | 0.458 | −0.0002 | (−0.010, 0.009) | 0.973 | −0.004 | (−0.039, 0.031) | 0.816 | \|  \| 0.0001 \| \| --- \| --- \| | (−0.006, 0.006) | 0.960 | 0.0003 | (−0.011, 0.012) | \|  \| 0.962 \| \| --- \| --- \| | −0.004 | (−0.034, 0.025) | 0.767 |
| Paced QRS Duration at Implantation | −0.104 | (−0.368, 0.160) | 0.441 | −0.038 | (−0.124, 0.048) | \|  \| 0.386 \| \| --- \| --- \| | −0.016 | (−0.128, 0.097) | 0.783 | \|  \| −0.035 \| \| --- \| --- \| | (−0.093, 0.023) | 0.238 | −0.045 | (−0.232, 0.142) | \|  \| 0.638 \| \| --- \| --- \| | −0.095 | (−0.143, −0.046) | 0.00013 |
| Paced QRS Duration at Follow-up | 0.015 | (−0.168, 0.198) | 0.869 | 0.017 | (−0.005, 0.038) | 0.130 | 0.078 | (0.011, 0.144) | 0.022 | 0.010 | (−0.003, 0.024) | 0.129 | 0.021 | (−0.008, 0.050) | 0.149 | −0.104 | (−0.159, −0.049) | 0.00022 |
| Lead Displacement at Implantation | 0.027 | (−0.100, 0.153) | 0.679 | −0.007 | (−0.085, 0.071) | 0.861 | 0.015 | (−0.088, 0.118) | 0.774 | 0.006 | (−0.044, 0.056) | 0.804 | 0.009 | (−0.141, 0.160) | 0.903 | 0.032 | (−0.222, 0.287) | 0.803 |
| Lead Displacement at Follow-up | 0.100 | (−0.065, 0.265) | 0.233 | −0.003 | (−0.013, 0.007) | 0.543 | 0.006 | (−0.060, 0.072) | 0.853 | −0.0004 | (−0.008, 0.007) | 0.928 | −0.002 | (−0.016, 0.013) | 0.833 | −0.332 | (−0.758, 0.093) | 0.126 |
| Impedance at Implantation | 0.033 | (−0.119, 0.184) | 0.673 | 0.001 | (−0.021, 0.023) | 0.949 | 0.010 | (−0.056, 0.075) | 0.773 | 0.001 | (−0.012, 0.014) | 0.908 | 0.001 | (−0.028, 0.029) | 0.968 | 0.010 | (−0.042, 0.062) | 0.709 |
| Impedance at Follow-up | −0.035 | (−0.142, 0.072) | 0.521 | −0.002 | (−0.017, 0.014) | 0.838 | −0.002 | (−0.037, 0.033) | 0.919 | −0.001 | (−0.011, 0.009) | 0.842 | 0.001 | (−0.020, 0.021) | 0.949 | 0.002 | (−0.024, 0.029) | 0.861 |
| Fluoroscopy time (min) at implantation | −0.0195 | (−0.0718, 0.0329) | 0.467 | −0.0123 | (−0.0269, –0.0023) | 0.098 | −0.0195 | (−0.0368, −0.0022) | 0.027 | −0.0093 | (−0.0189, 0.0004) | 0.059 | −0.0363 | (−0.0667, −0.0058) | 0.019 | −0.0128 | (−0.0183, −0.0072) | 7.58E-06 |
| Change in pacing threshold at implantation | 0.0007 | (−0.2203, 0.2218) | 0.995 | −0.0015 | (−0.0832, 0.0803) | 0.972 | −0.0054 | (−0.2469, 0.2361) | 0.965 | −0.0003 | (−0.0602, 0.0596) | 0.992 | −0.0033 | (−0.1680, 0.1614) | 0.969 | NA | NA | NA |
| Change in pacing threshold at follow‑up | −0.0136 | (−0.1700, 0.1427) | 0.864 | 0.0035 | (−0.0204, 0.0274) | 0.773 | 0.0030 | (−0.0459, 0.0518) | 0.905 | 0.0054 | (−0.0187, 0.0295) | 0.659 | 0.0099 | (−0.0525, 0.0722) | 0.756 | 0.0011 | (−0.0220, 0.0242) | 0.923 |

**Supplementary Table S5:** Meta-Regression Analysis of Procedural Outcomes


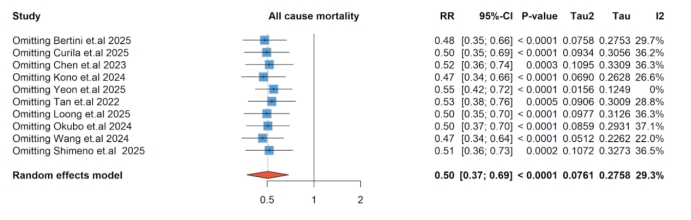


**Figure S2:** Leave-One_Out Plot of All-cause mortality


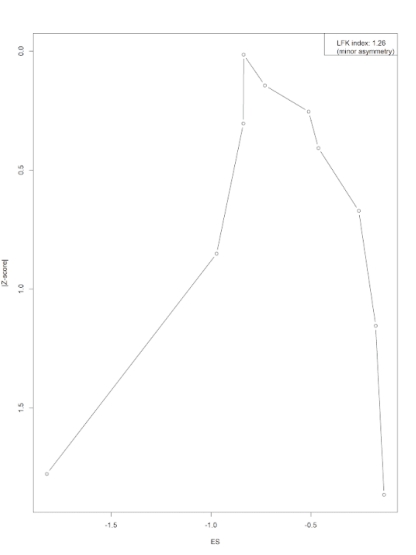


**Figure S3:** LFK Plot of All-cause mortality


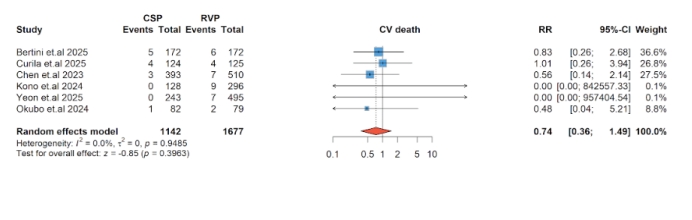


**Figure S4:** Forest Plot of Cardiovascular (CV) death


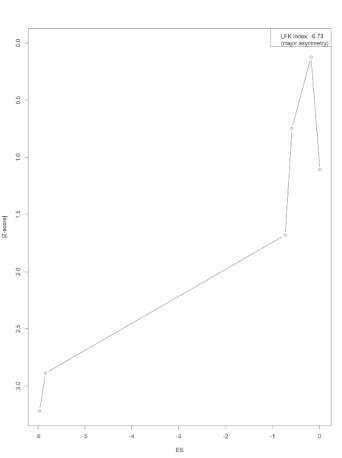


**Figure S5:** LFK Plot of Cardiovascular (CV) death


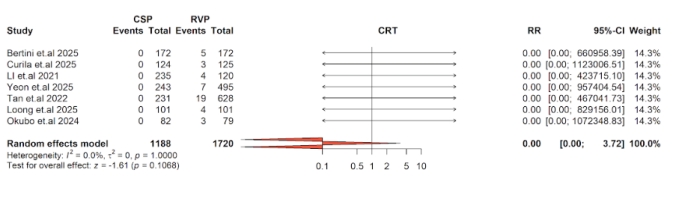


**Figure S6:** Forest Plot of Cardiac Resynchronization Therapy (CRT)


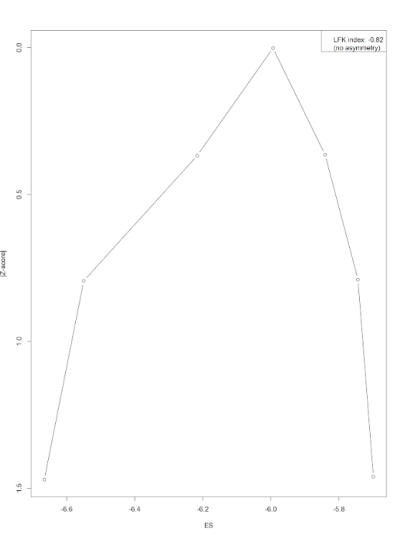


**Figure S7:** LFK Plot of Cardiac Resynchronization Therapy (CRT)


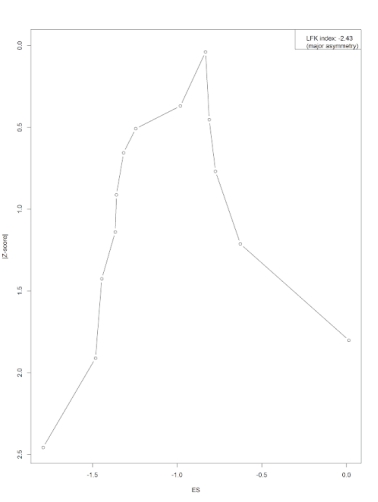


**Figure S8:** LFK Plot of HF hospitalization


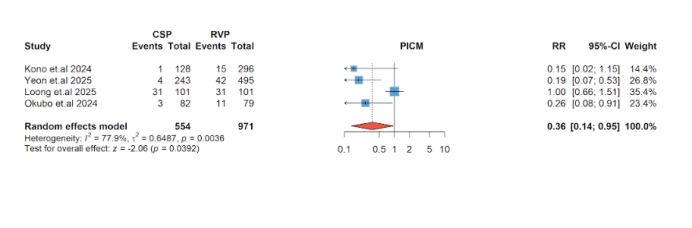


**Figure S9:** Forest Plot of Pacing induced cardiomyopathy (PICM)


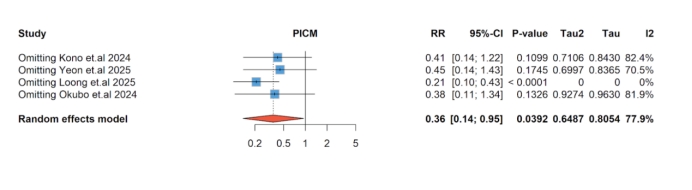


**Figure S10:** Leave-One_Out Plot of Pacing induced cardiomyopathy (PICM)


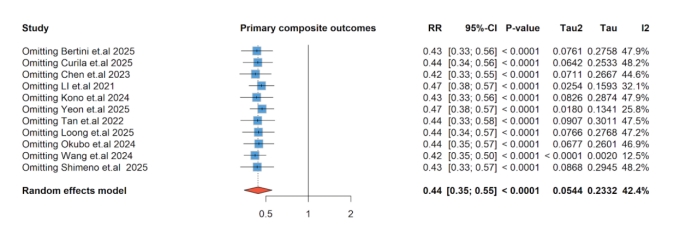


**Figure S11:** Leave-One_Out Plot of *Primary composite outcomes*


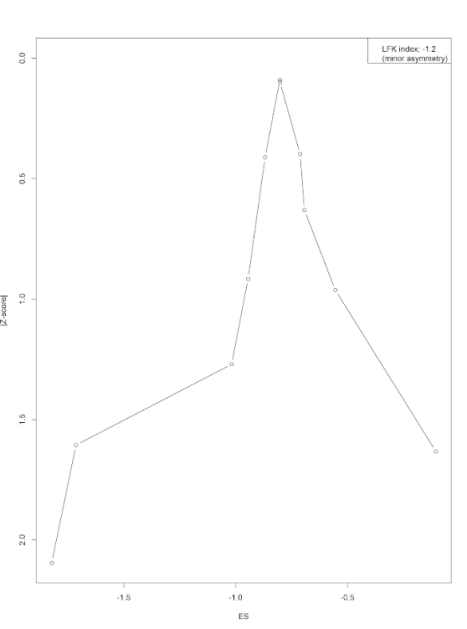


**Figure S12:** LFK Plot of *Primary composite outcomes*


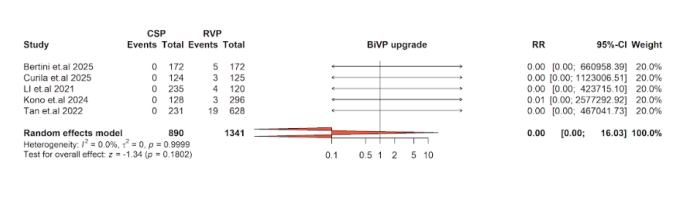


**Figure S13:** Forest Plot of Biventricular Pacing (BiVP) upgrade


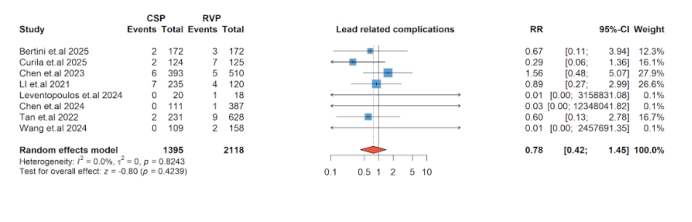


**Figure S14:** Forest Plot of Lead-related complications


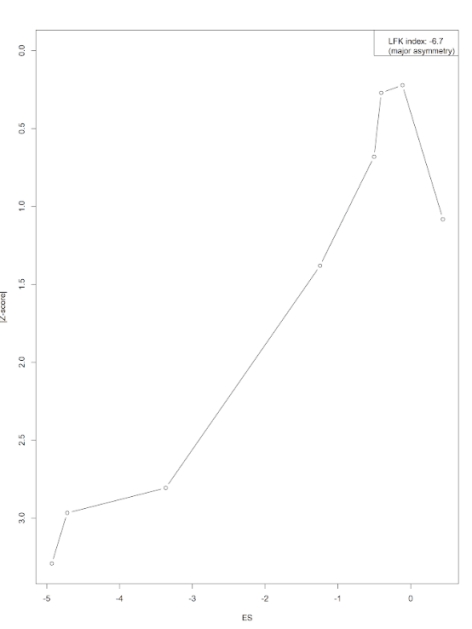


**Figure S15:** LFK Plot of lead-related complications


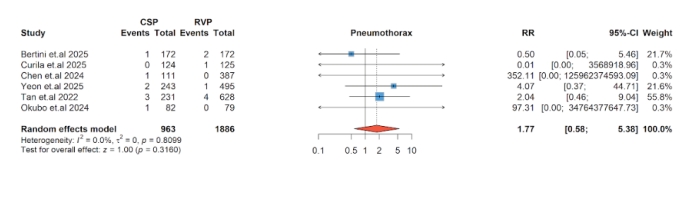


**Figure S16:** Forest Plot of Pneumothorax


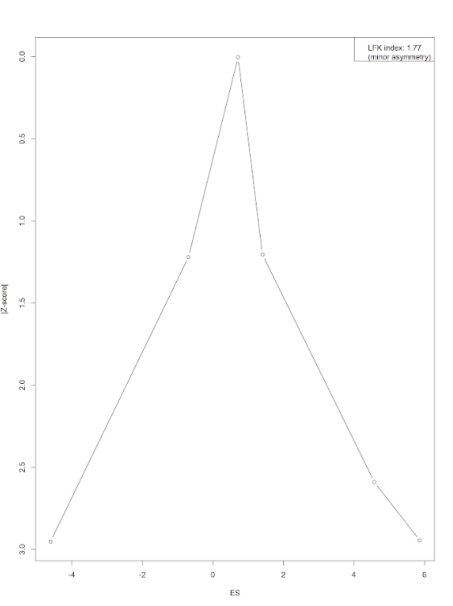


**Figure S17:** LFK Plot of Pneumothorax


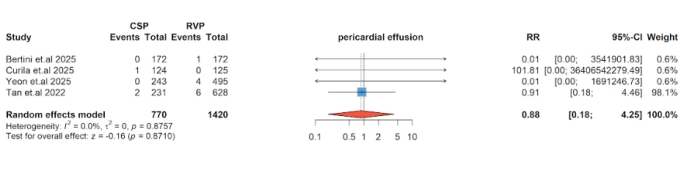


**Figure S18:** Forest Plot of Pericardial effusion


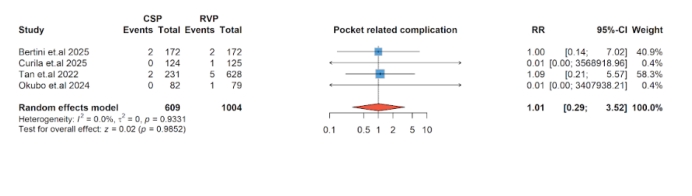


**Figure S19:** Forest Plot of Pocket-related complications


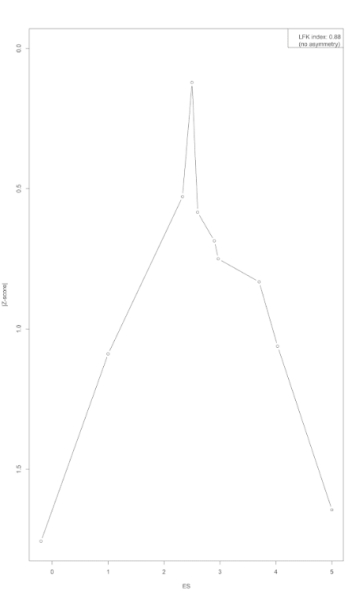


**Figure S20:** LFK Plot of LVEF


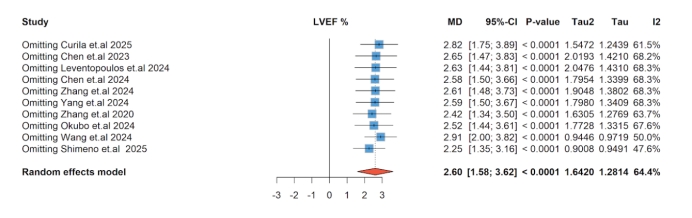


**Figure S21:** Leave-One_Out Plot of LVEF


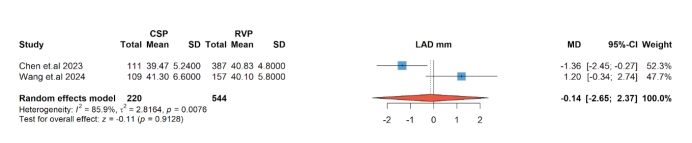


**Figure S22: Forest Plot of** Left atrial diameter LAD


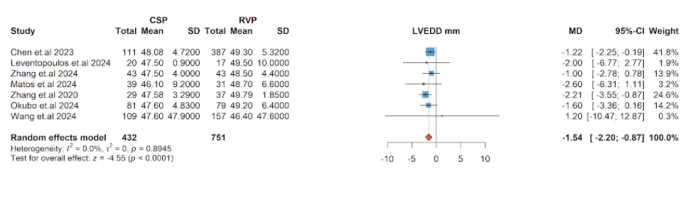


**Figure S23:** Forest Plot of Left ventricular end-diastolic diameter (LVEDD)


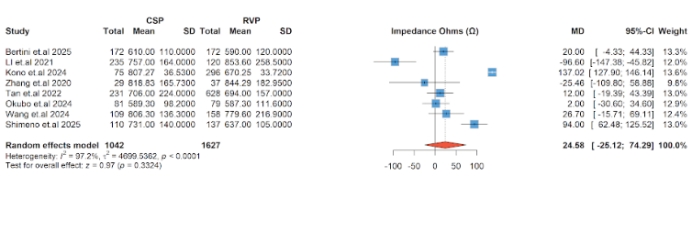


**Figure S24:** Forest Plot of Impedance at implantation


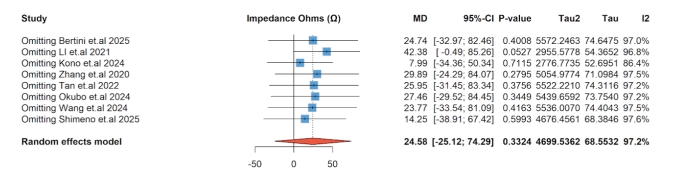


**Figure S25:** Leave-One_Out Plot of Impedance at implantation


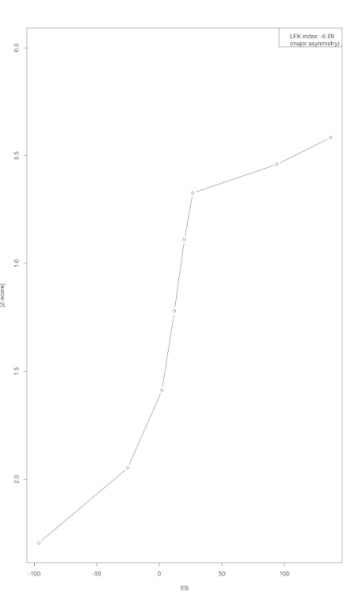


**Figure S26:** LFK Plot of of Impedance at implantation


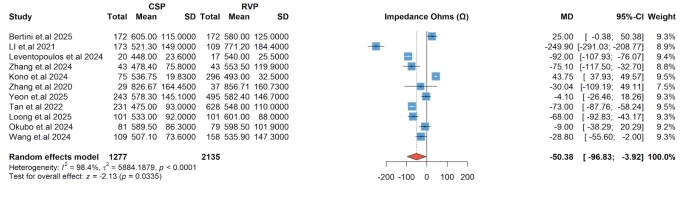


**Figure S27:** Forest Plot of Impedance at followup


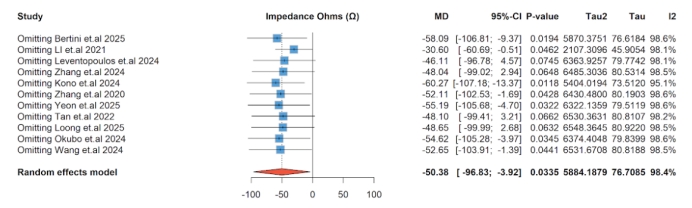


**Figure S28:** Leave-One_Out Plot of Impedance at follow up


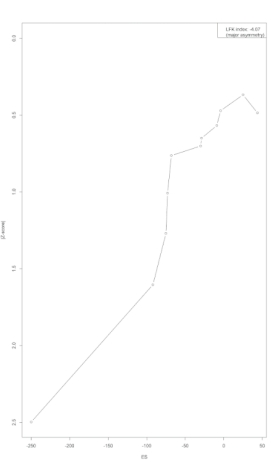


**Figure S29:** LFK Plot of Impedance at follow up


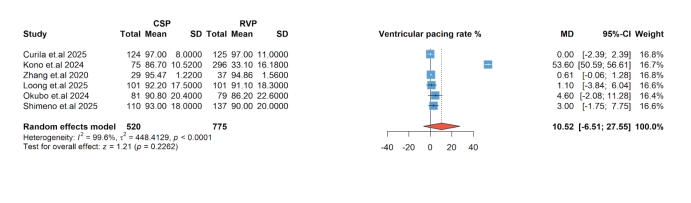


**Figure S30:** Forest Plot of Ventricular pacing rate


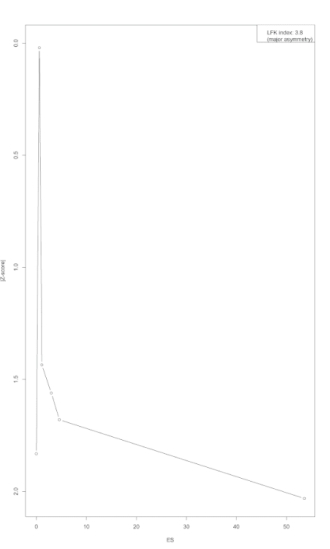


**Figure S31:** LFK Plot of Ventricular pacing rate


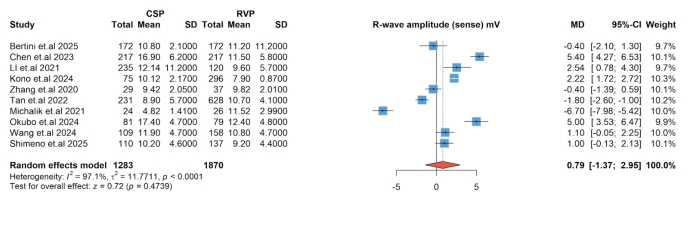


**Figure S32:** Forest Plot of R-wave amplitude at implantation


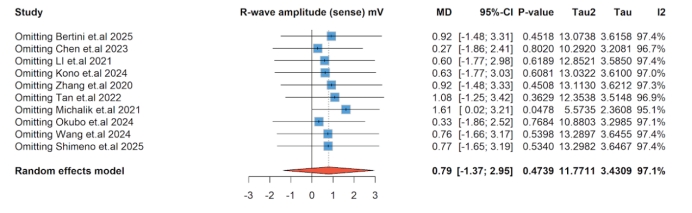
**Figure S33:** Leave-one-out Plot of R-wave amplitude at implantation


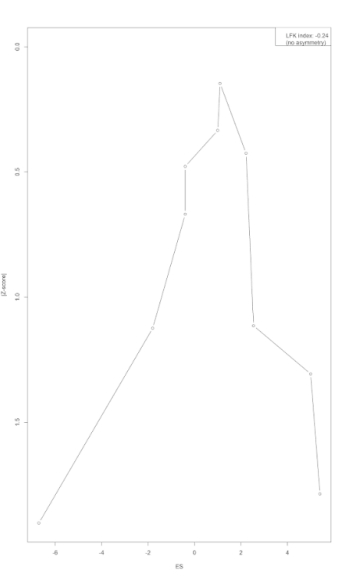


**Figure S34:** LFK Plot of R-wave amplitude at implantation


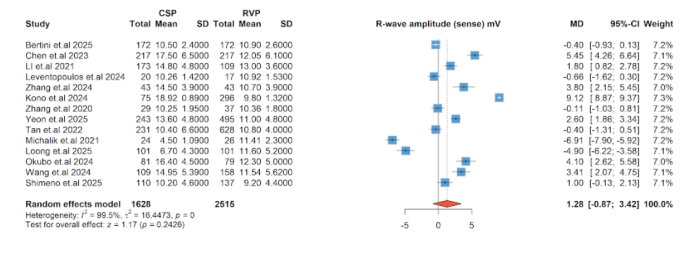
**Figure S35:** Forest Plot of R-wave amplitude at follow up


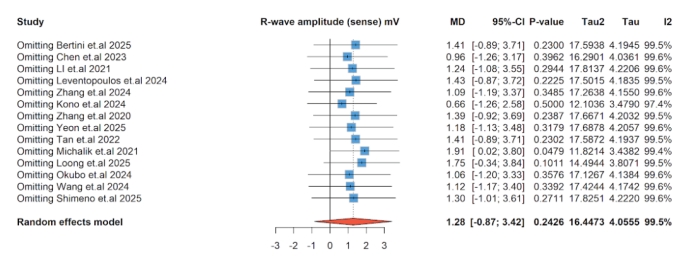


**Figure S36:** Leave one out Plot of R-wave amplitude at follow up


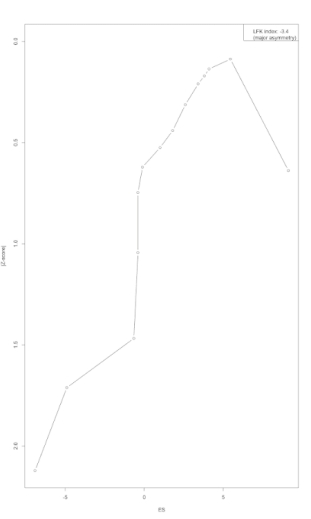


**Figure S37:** Leave one out Plot of R-wave amplitude at follow up


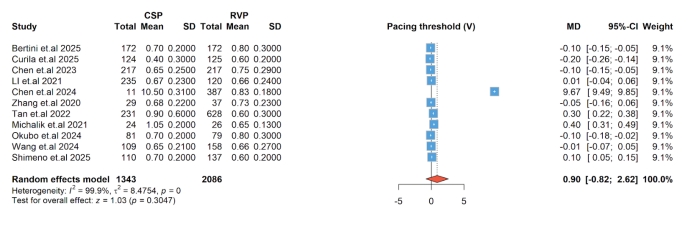


**Figure S38:** Forest Plot of Pacing threshold At implantation


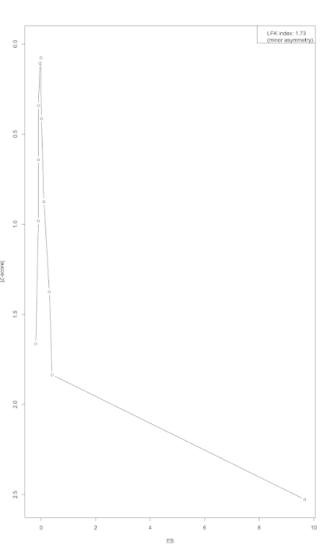


**Figure S39:** LFK Plot of Pacing threshold At implantation


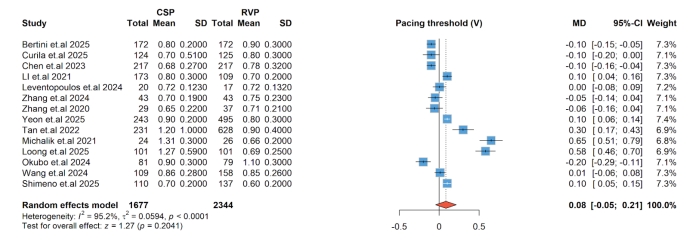


**Figure S40:** Forest Plot of Pacing threshold at follow up


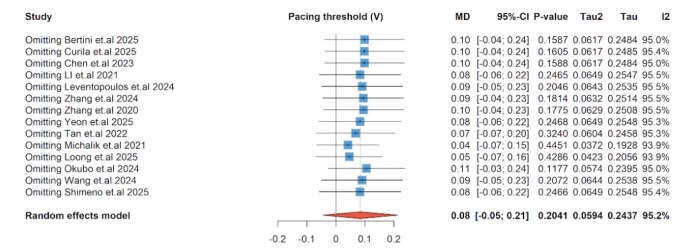


**Figure S41:** Leave one out Plot of Pacing threshold at follow up


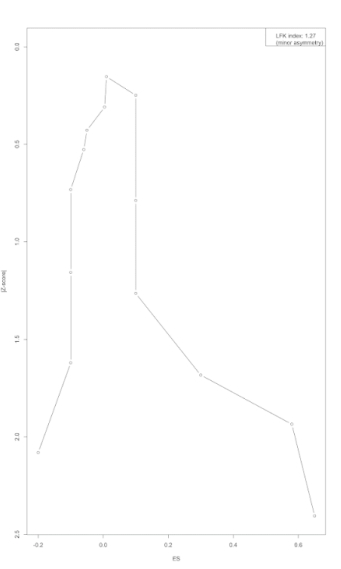


**Figure S42:** LFK Plot of Pacing threshold at follow up


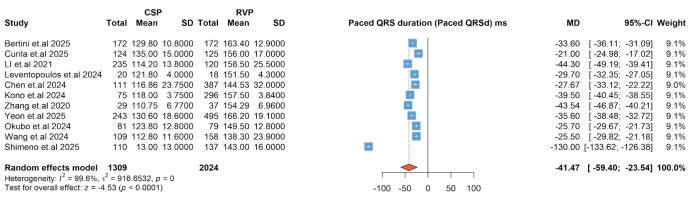


**Figure S43:** Forest Plot of Paced QRS duration at implantation


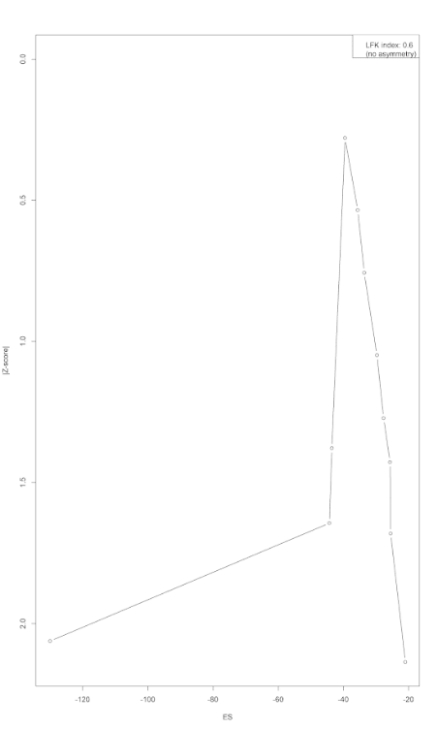


**Figure S44:** LFK Plot of Paced QRS duration at implantation


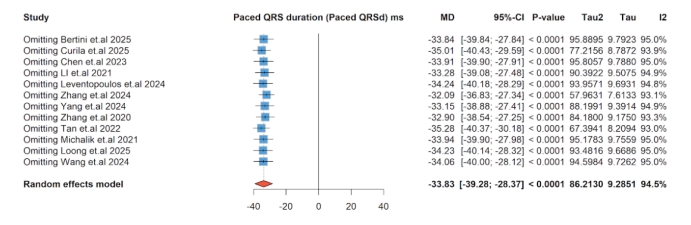


**Figure S45:** LFK Plot of Paced QRS duration at follow up


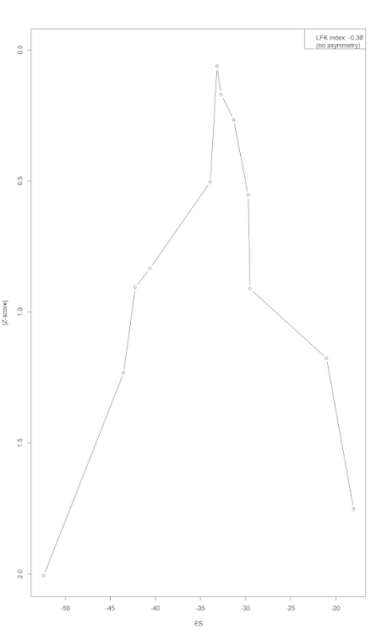


**Figure S46:** LFK Plot of Paced QRS duration at follow up


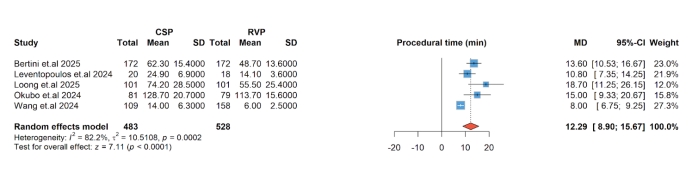


**Figure S47:** Forest Plot of Procedural time (min)


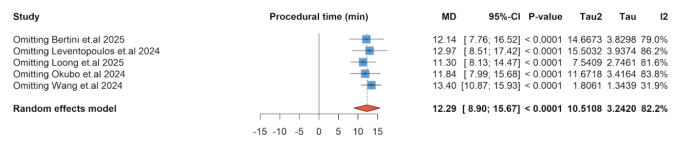


**Figure S48:** Leave one out Plot of Procedural time (min)


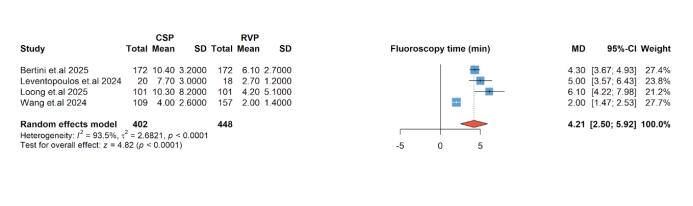


**Figure S49:** Forest Plot of Fluoroscopy time (min)


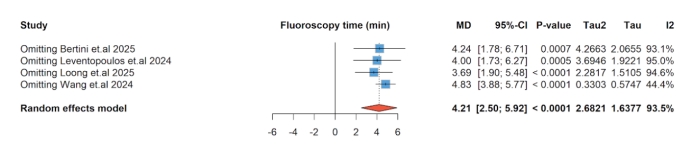


**Figure S50:** Leave one out Plot of Fluoroscopy time (min)


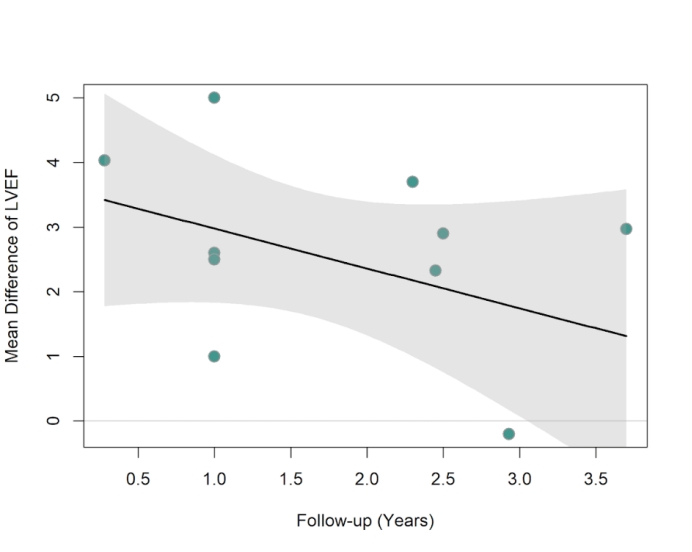


**Figure S51**: Meta-Regression of LVEF Change Over Follow-Up Duration


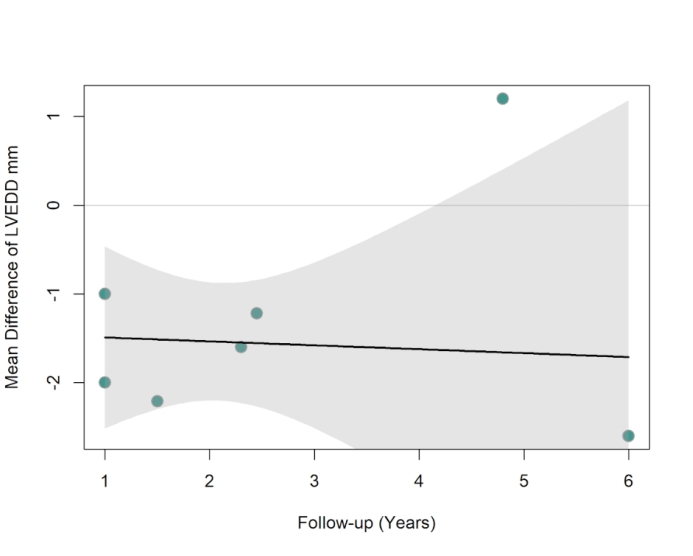


**Figure S52**: Meta-Regression of LVEDD Change Over Follow-Up Duration


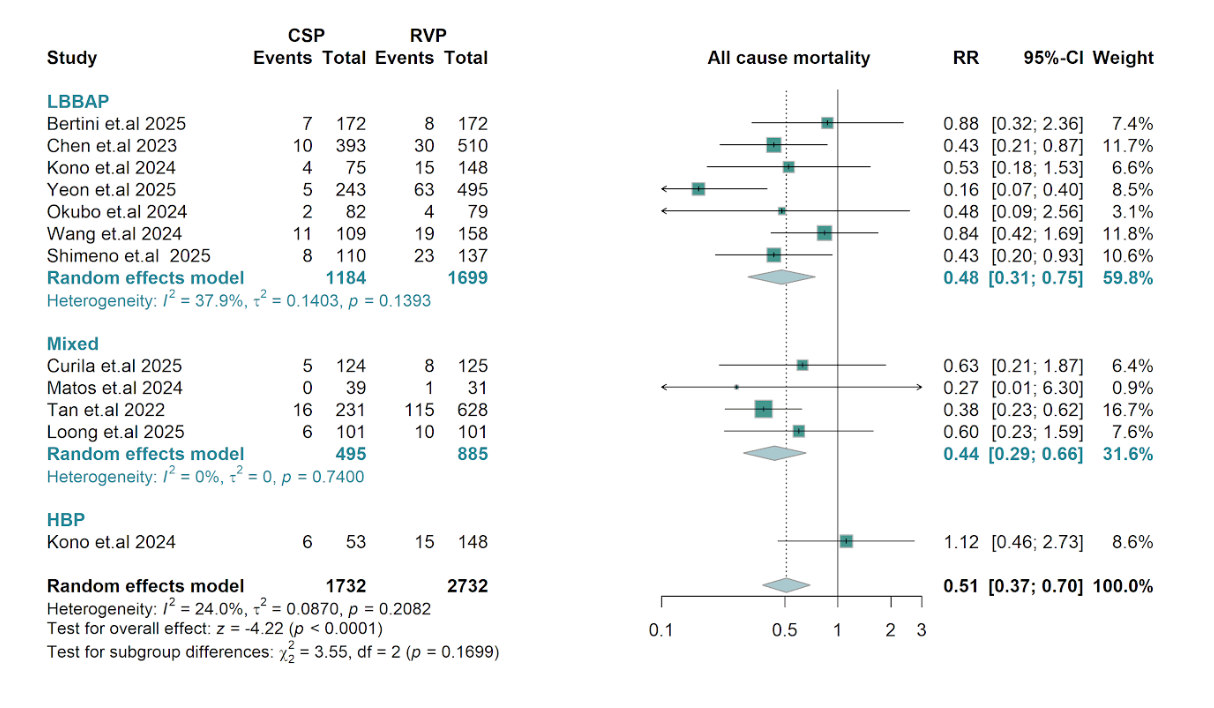


**Figure S53:** Forest Plot of Subgroup Analysis for All-Cause Mortality by CSP Modality


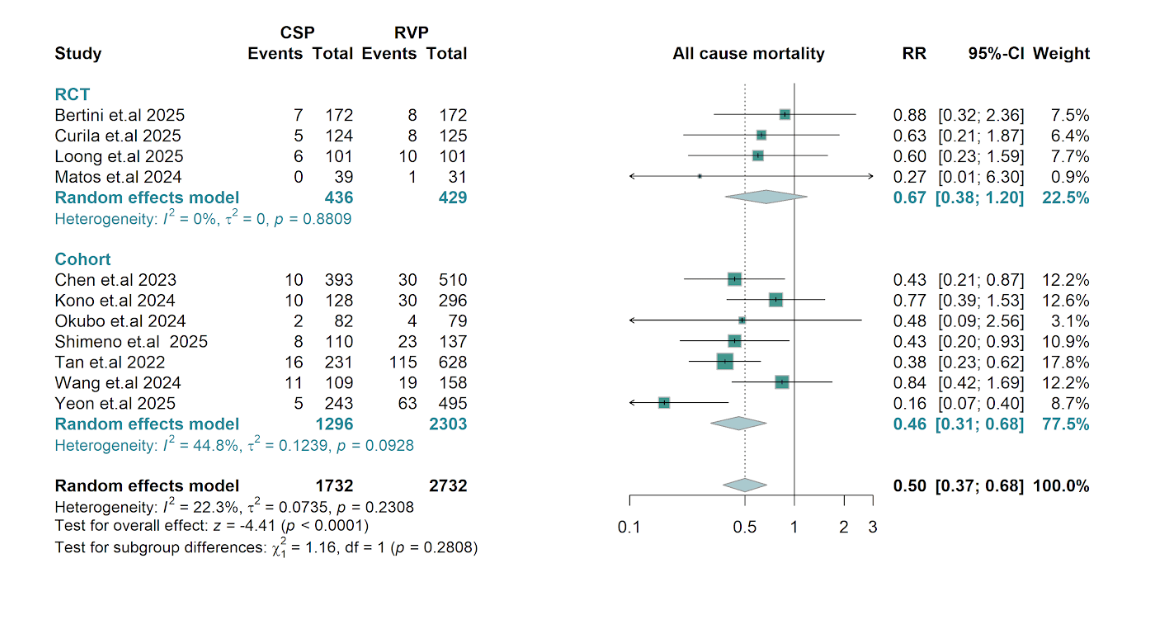


**Figure S54:** Forest Plot of Subgroup Analysis for All-Cause Mortality by Study Design


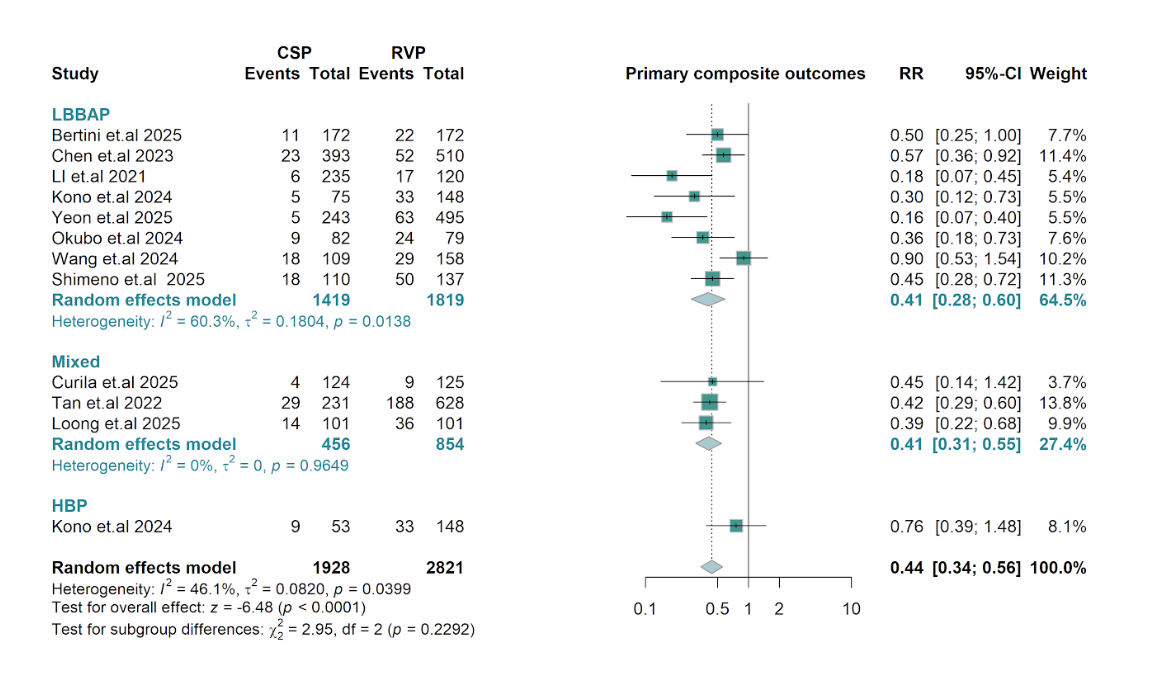


**Figure S55:** Forest Plot of Subgroup Analysis for Primary Composite Outcomes by CSP Modality


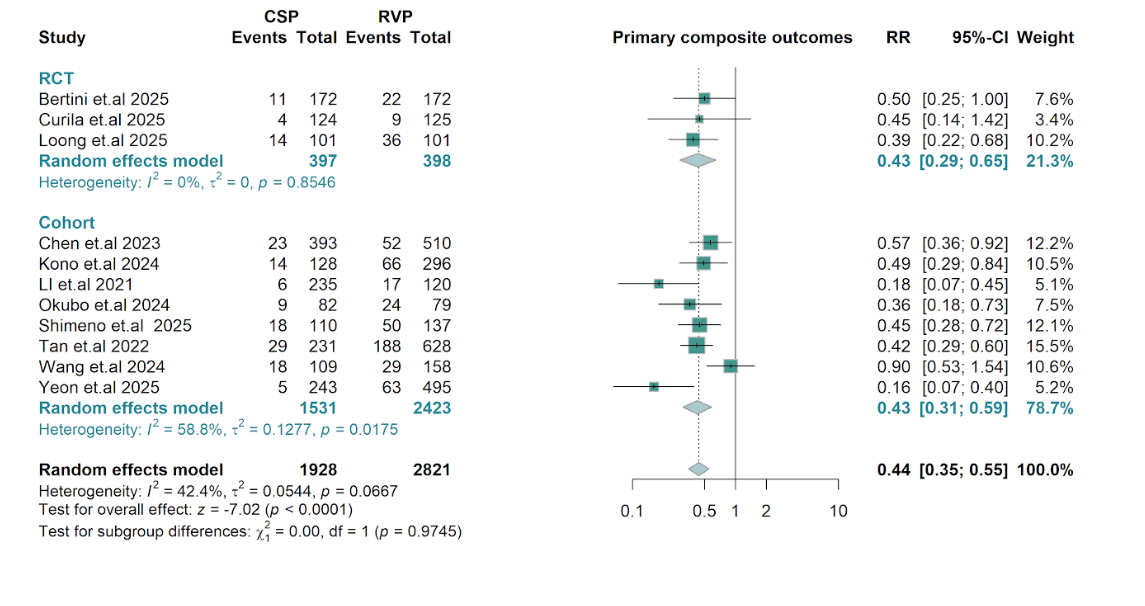


**Figure S56:** Forest Plot of Subgroup Analysis for Primary Composite Outcomes by Study Design


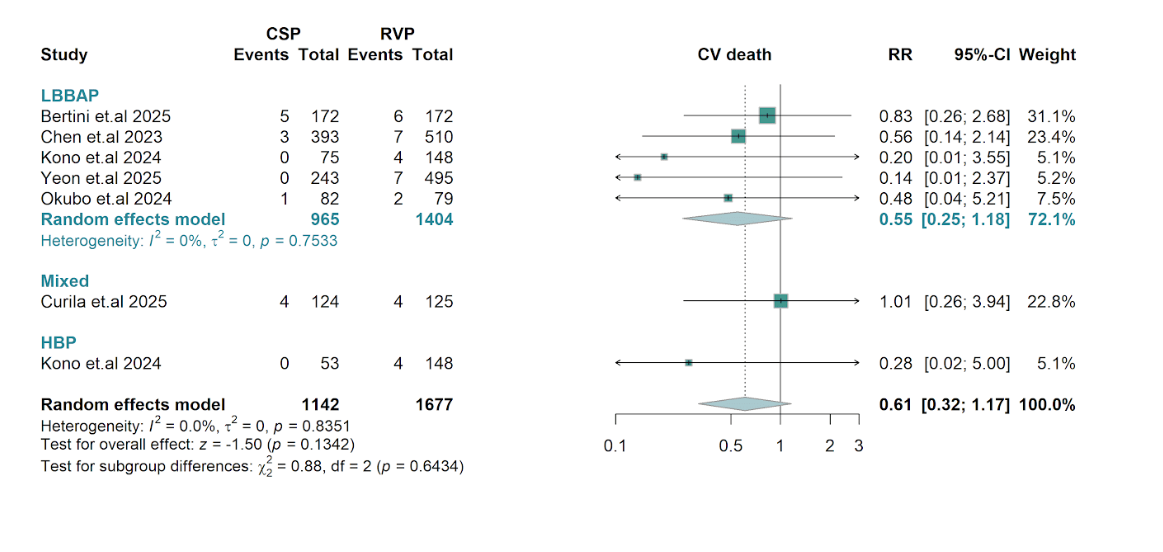


**Figure S57:** Forest Plot of Subgroup Analysis for Cardiovascular Death by CSP Modality


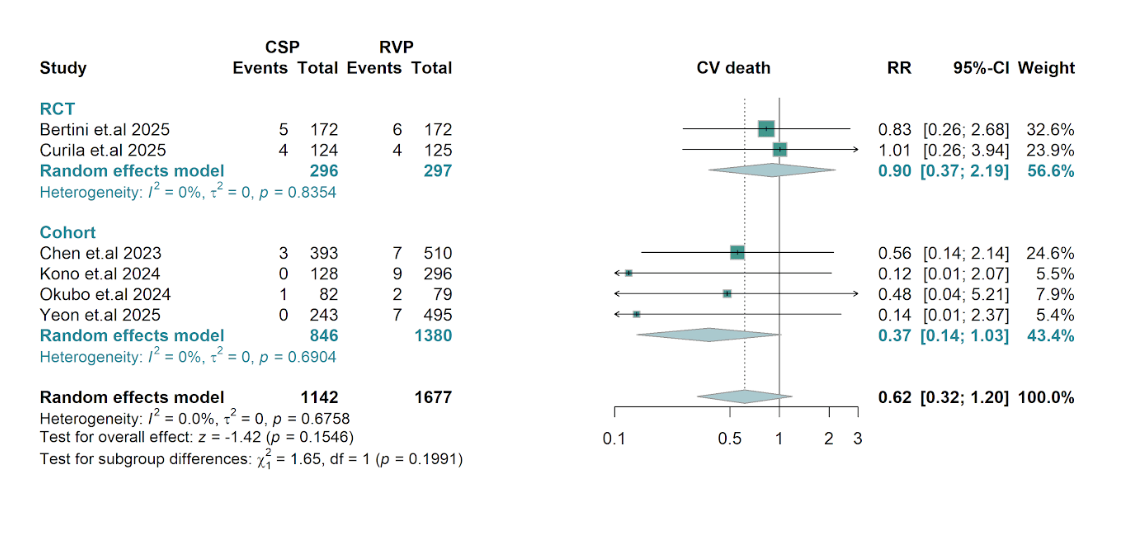


**Figure S58:** Forest Plot of Subgroup Analysis for Cardiovascular Death by Study Design


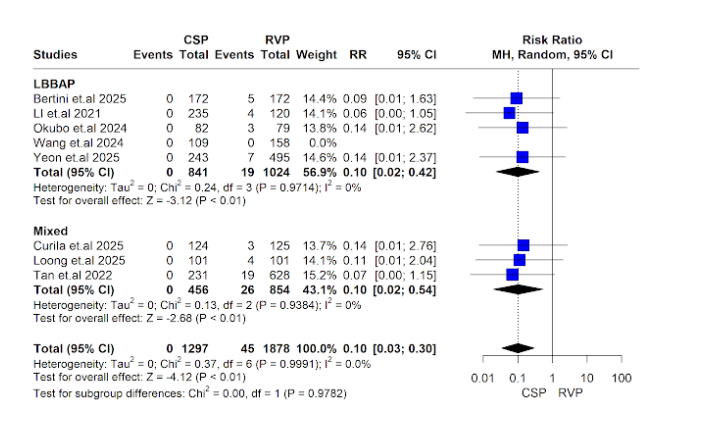


**Figure S59:** Forest Plot of Subgroup Analysis for CRT outcome by CSP Modality


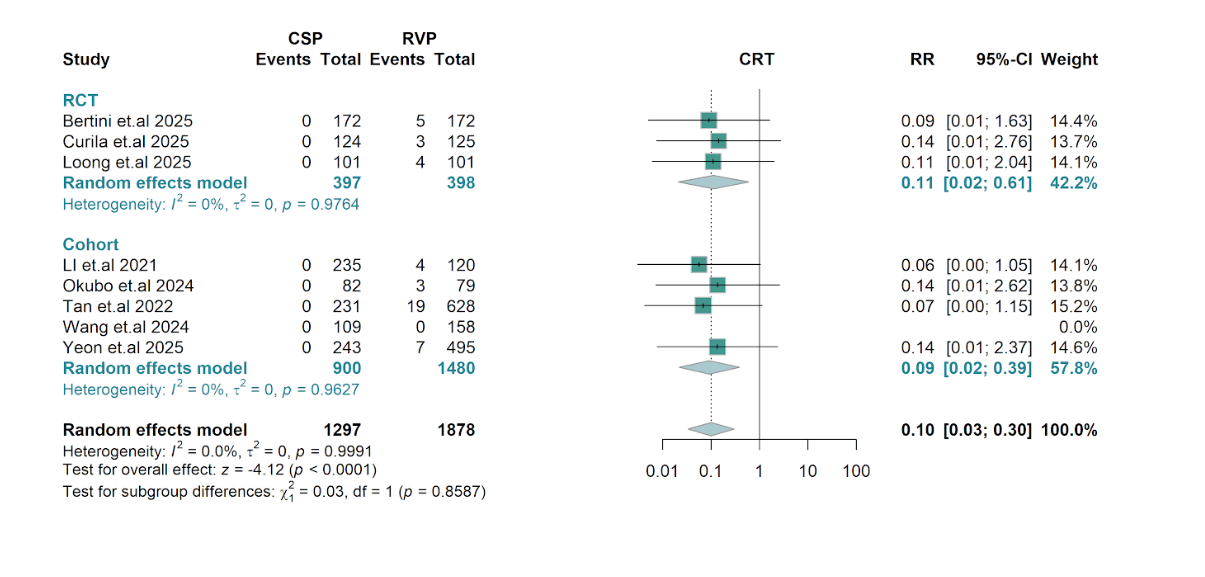


**Figure S60:** Forest Plot of Subgroup Analysis for CRT outcome by Study Design


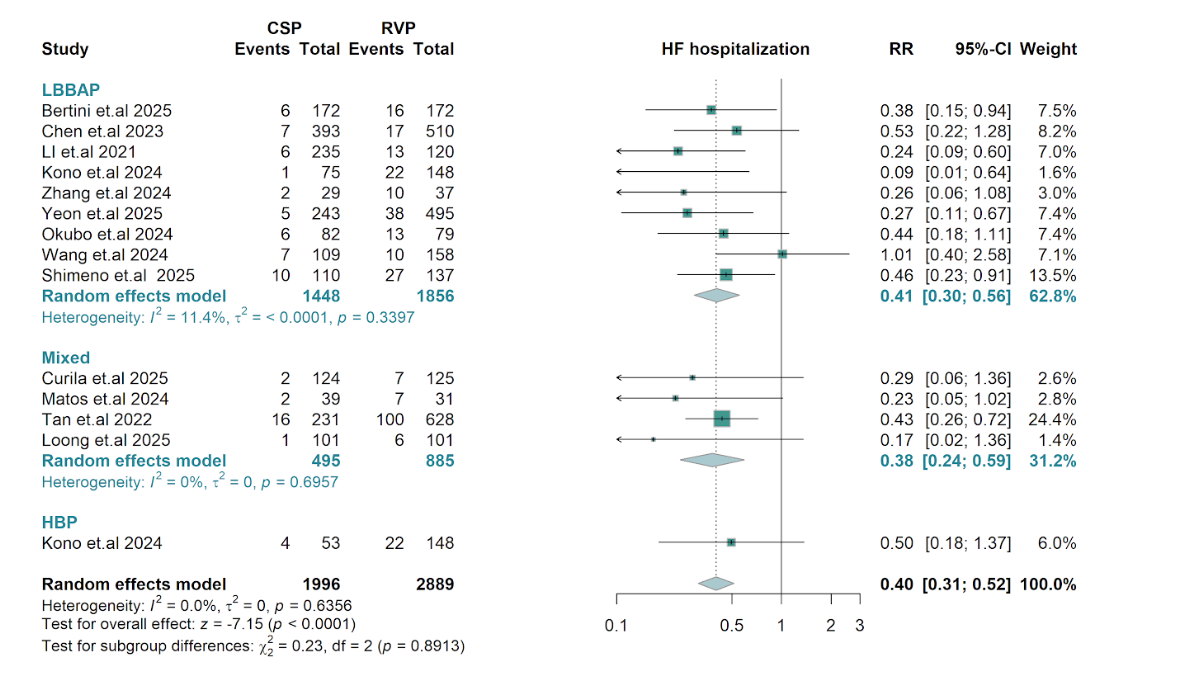


**Figure S61:** Forest Plot of Subgroup Analysis for Heart Failure Hospitalization by CSP Modality


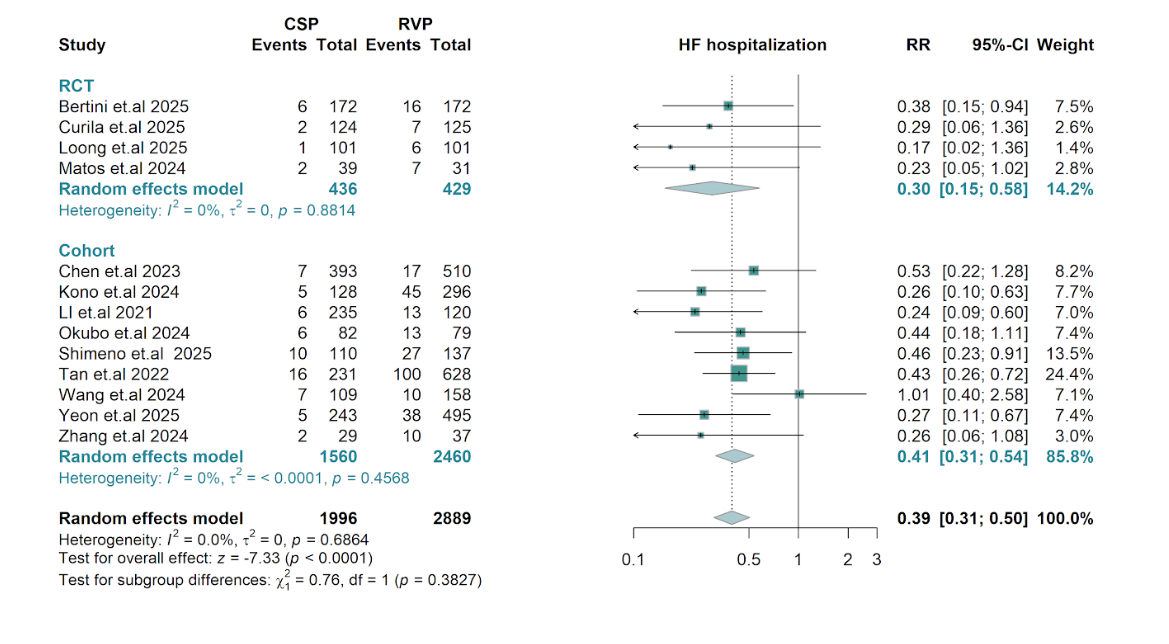


**Figure S62:** Forest Plot of Subgroup Analysis for Heart Failure Hospitalization by Study Design


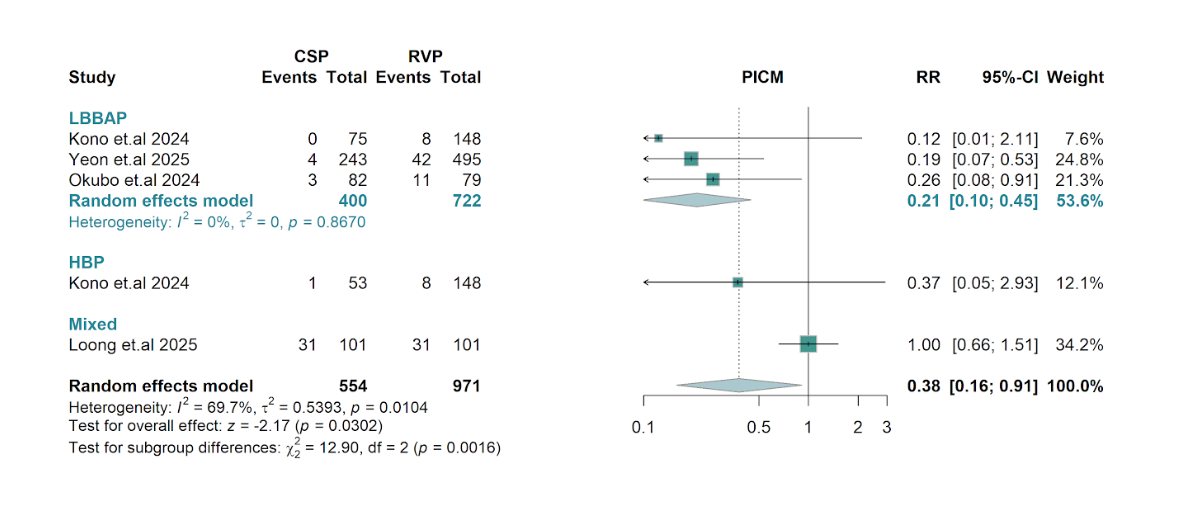


**Figure S63:** Forest Plot of Subgroup Analysis for PICM by CSP Modality


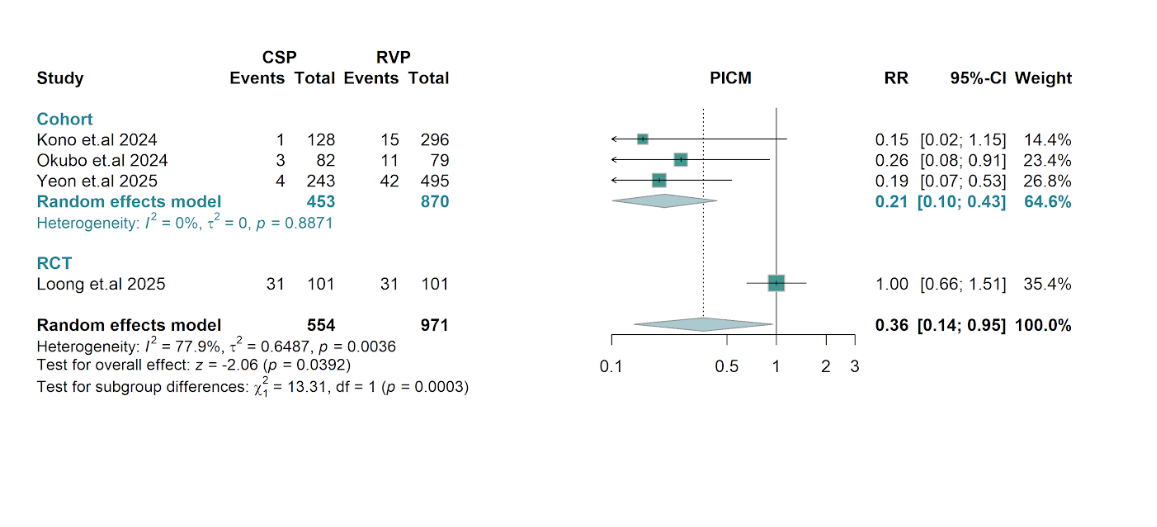


**Figure S64:** Forest Plot of Subgroup Analysis for PICM by Study Design


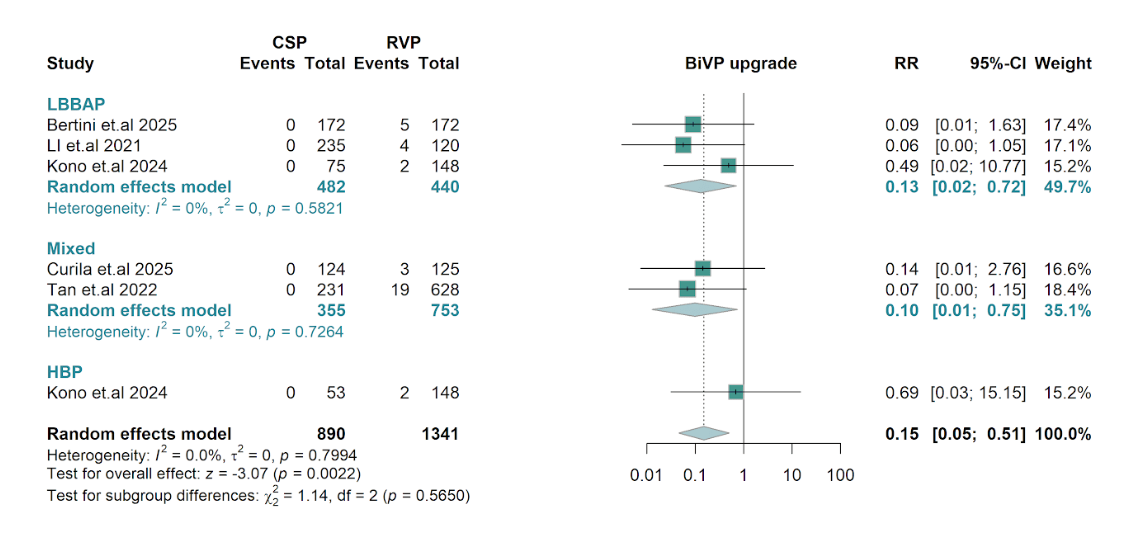


**Figure S65:** Forest Plot of Subgroup Analysis for BiVP Upgrade by CSP Modality


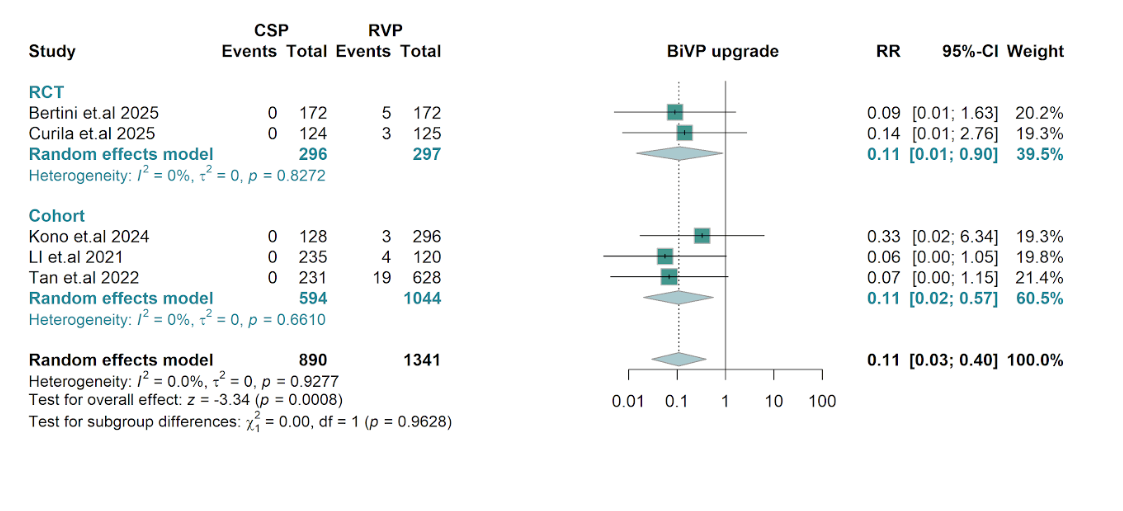


**Figure S66:** Forest Plot of Subgroup Analysis for BiVP Upgrade by Study Design


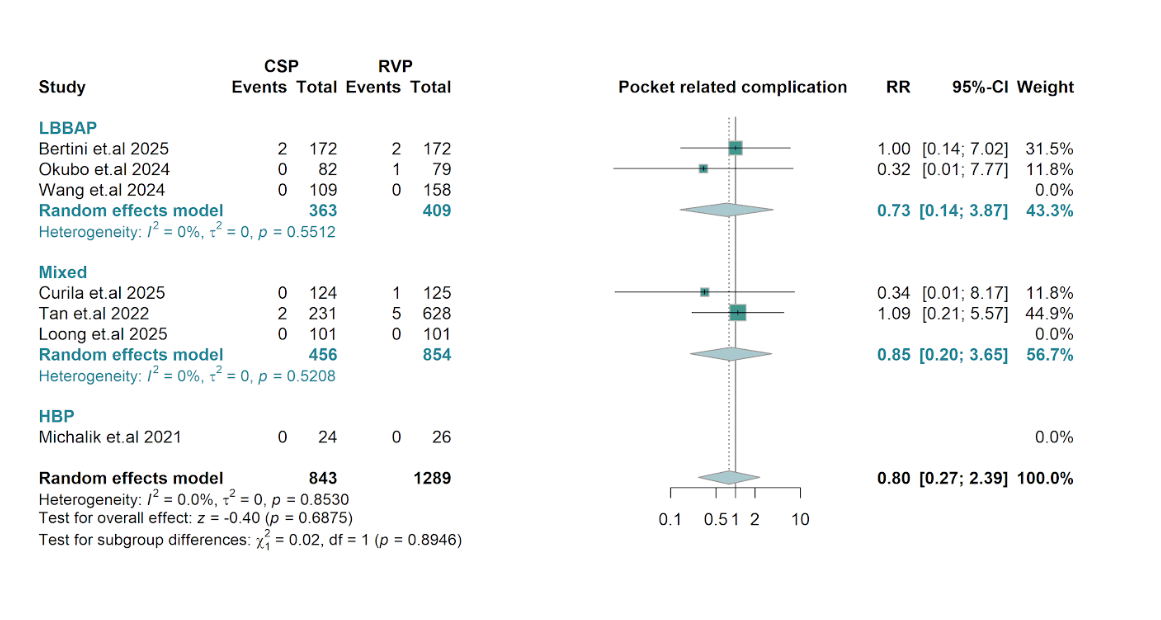


**Figure S67:** Forest Plot of Subgroup Analysis for Pocket related complication by CSP Modality


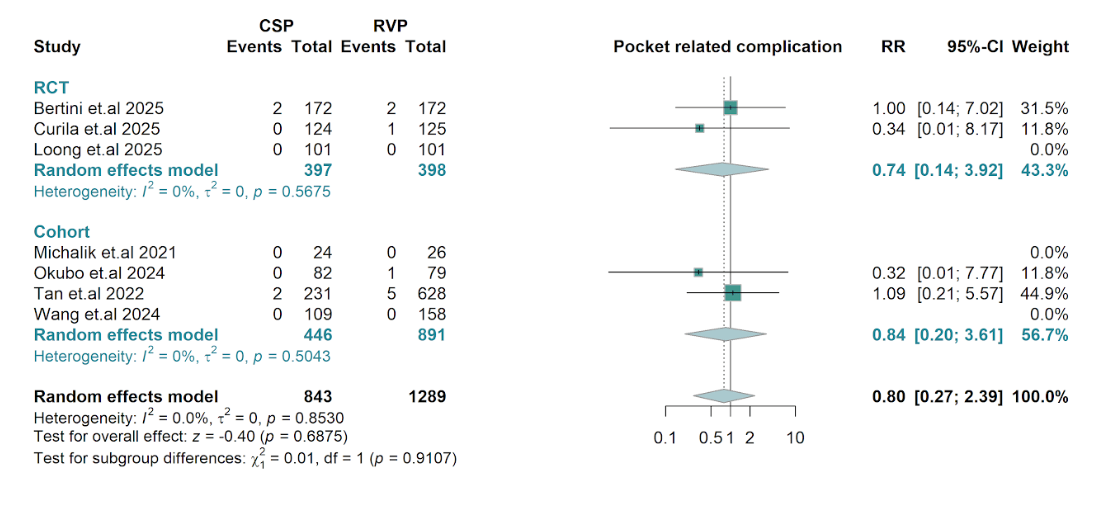


**Figure S68:** Forest Plot of Subgroup Analysis for Pocket related complications by Study Design


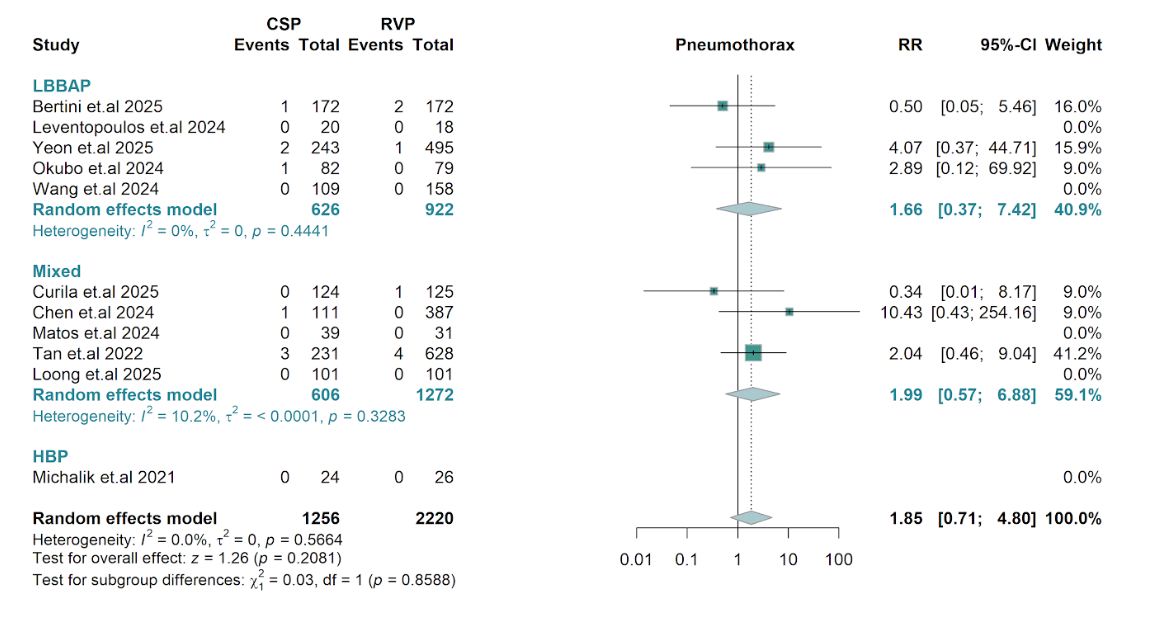


**Figure S69:** Forest Plot of Subgroup Analysis for Pneumothorax by CSP Modality


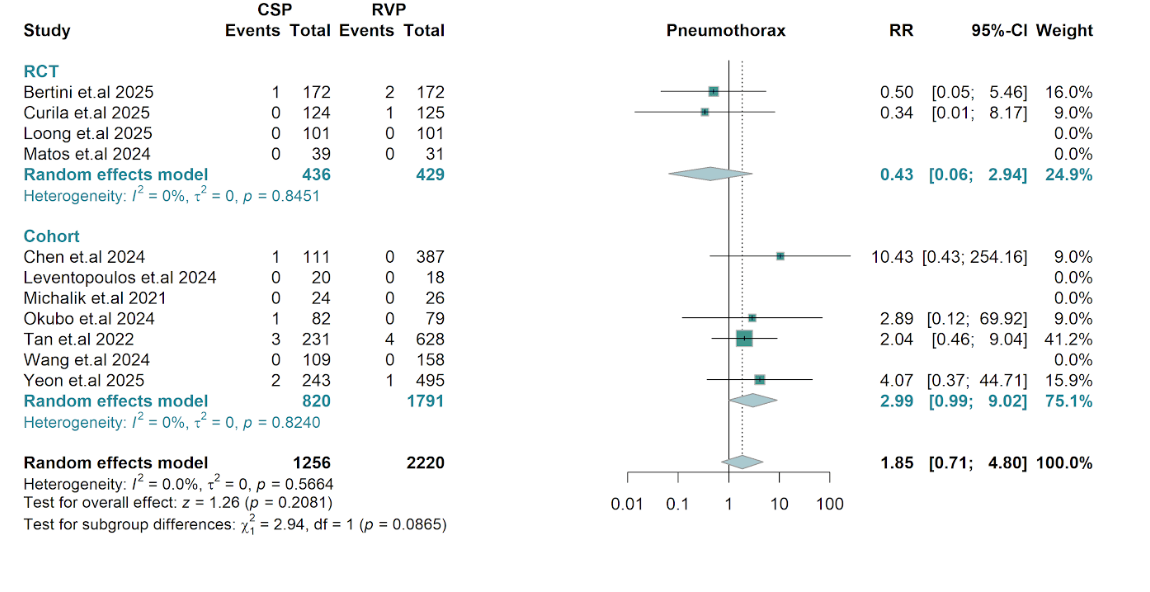


**Figure S70:** Forest Plot of Subgroup Analysis for Pneumothorax by Study Design


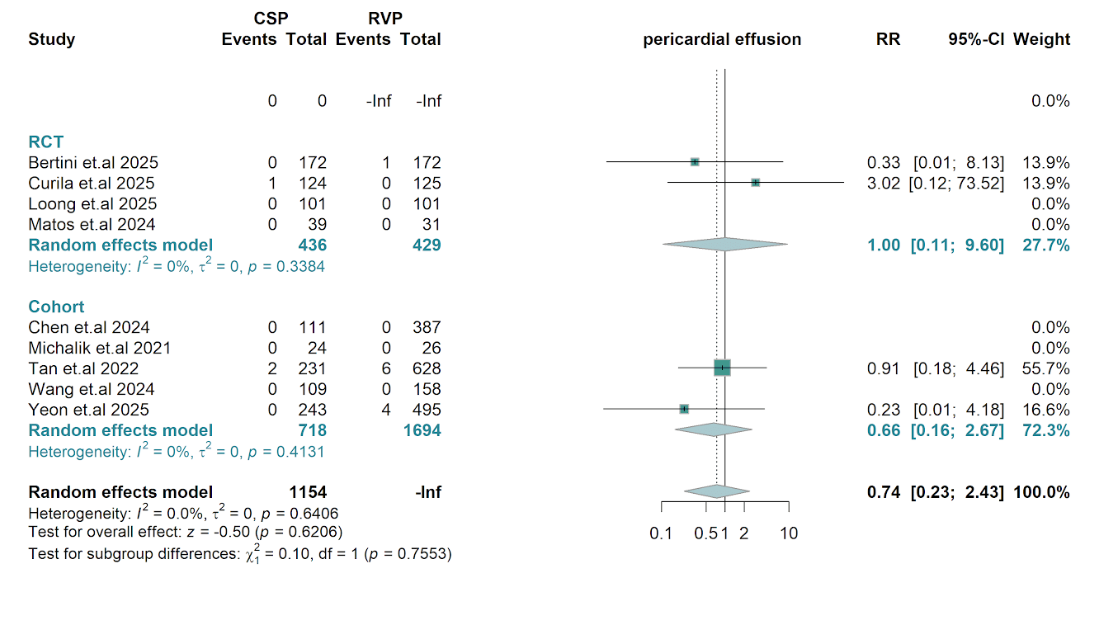


**Figure S71:** Forest Plot of Subgroup Analysis for Pericardial Effusion by CSP Modality


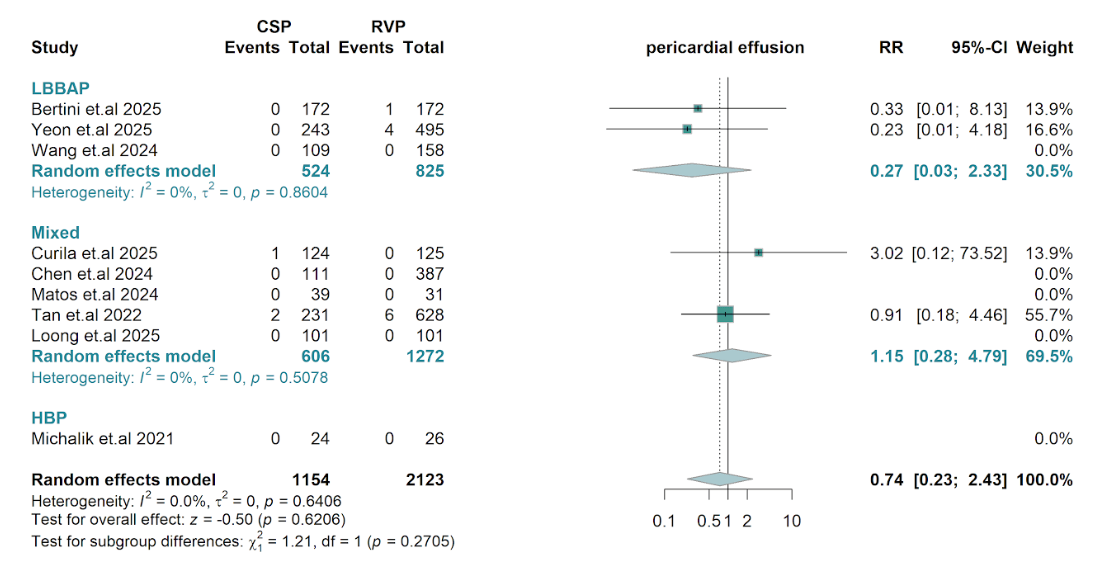


**Figure S72:** Forest Plot of Subgroup Analysis for Pericardial Effusion Study Design


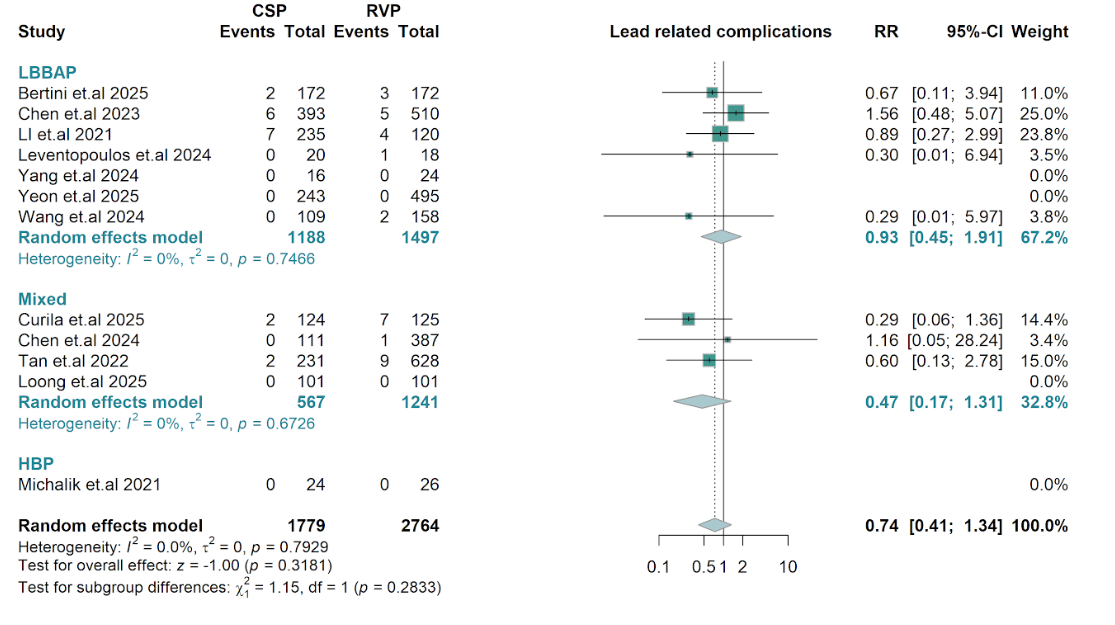


**Figure S73:** Forest Plot of Subgroup Analysis for Lead related complications by CSP Modality


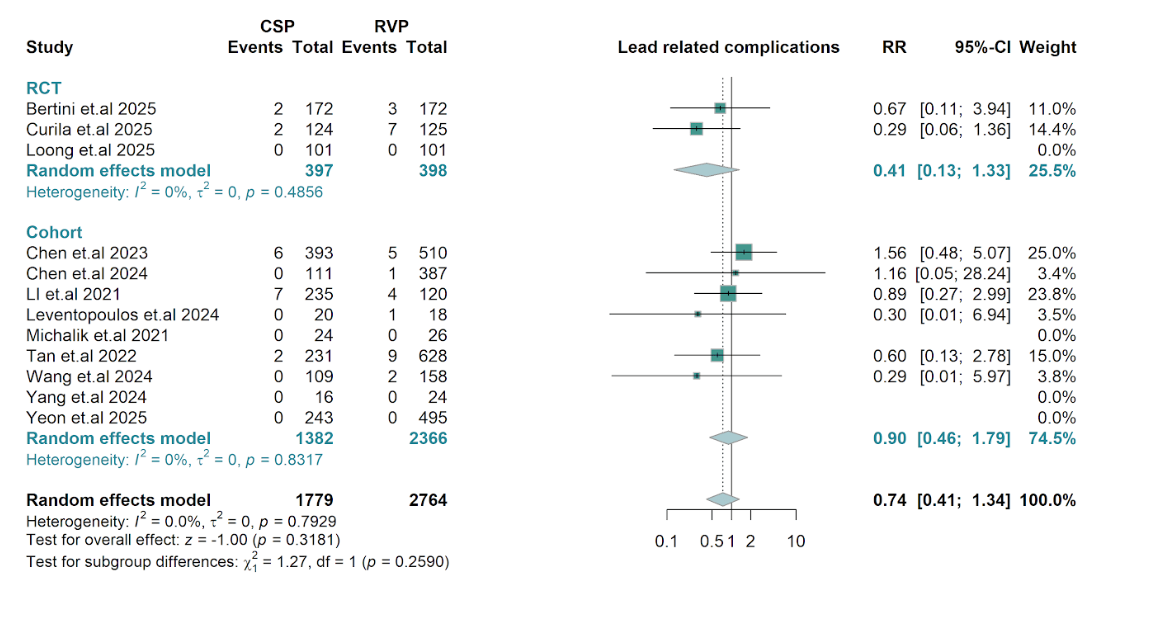


**Figure S74:** Forest Plot of Subgroup Analysis for Lead related complications by Study Design


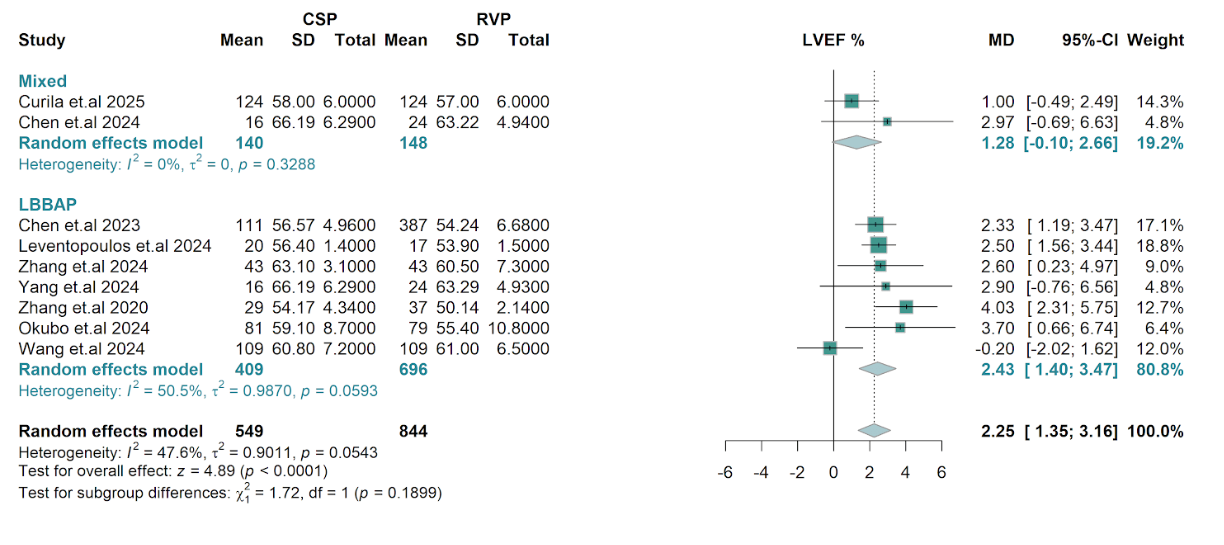


**Figure S75:** Forest Plot of Subgroup Analysis for LVEF % by CSP Modality


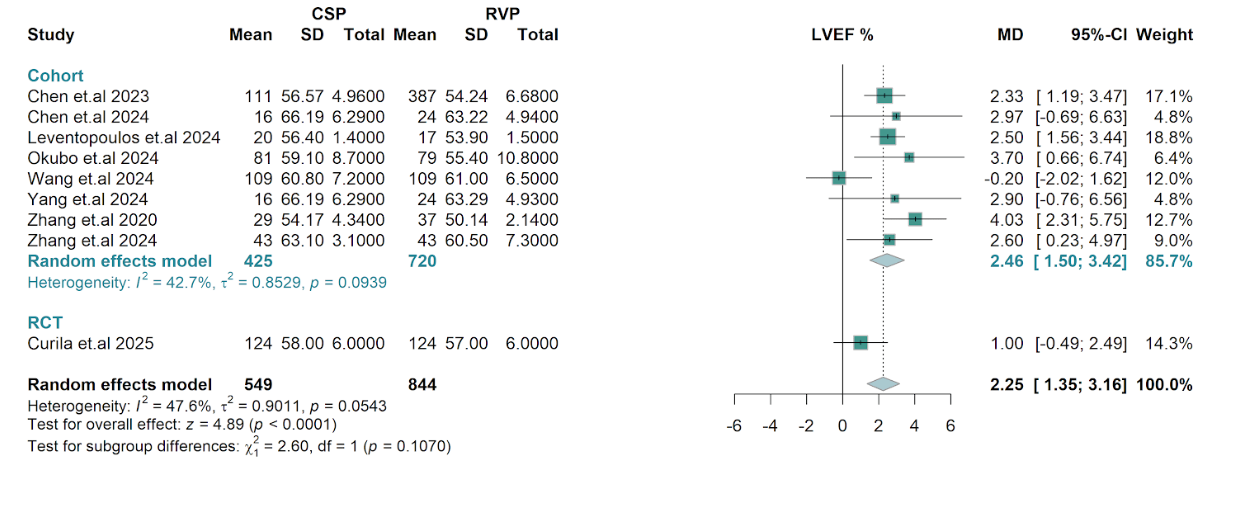


**Figure S76:** Forest Plot of Subgroup Analysis for LVEF % by Study Design


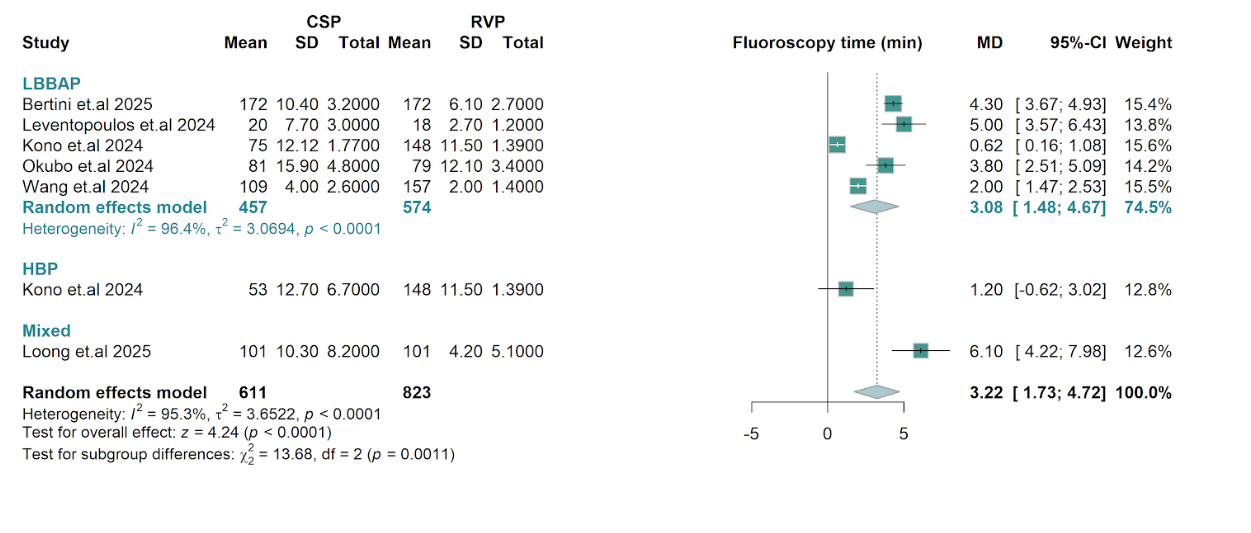


**Figure S77:** Forest Plot of Subgroup Analysis for Fluoroscopy time at implantation by CSP Modality


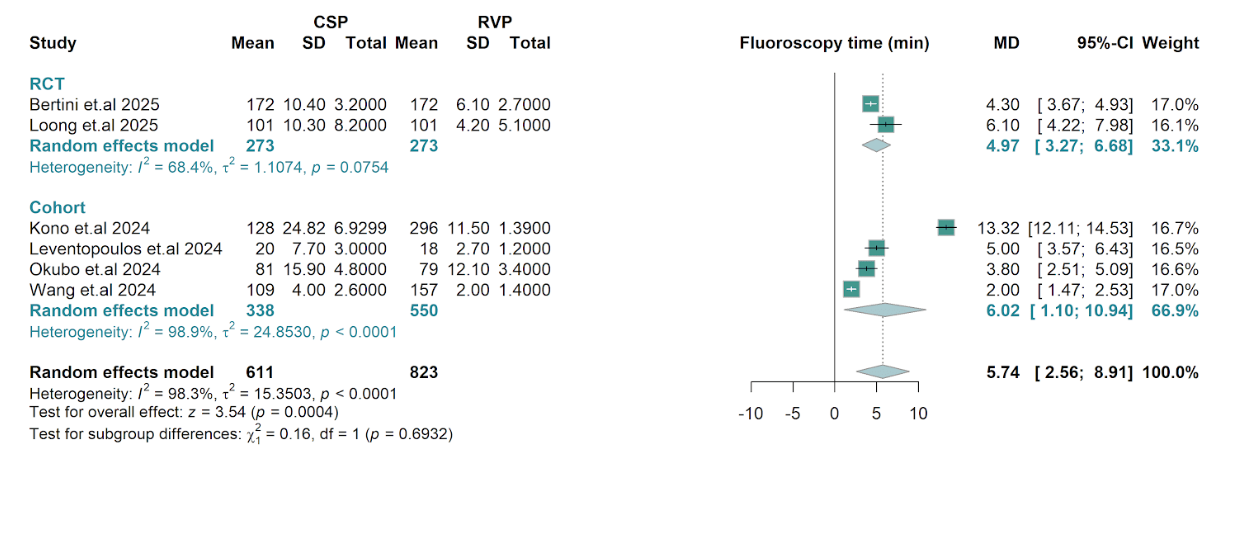


**Figure S78:** Forest Plot of Subgroup Analysis for Fluoroscopy time at implantation by Study Design


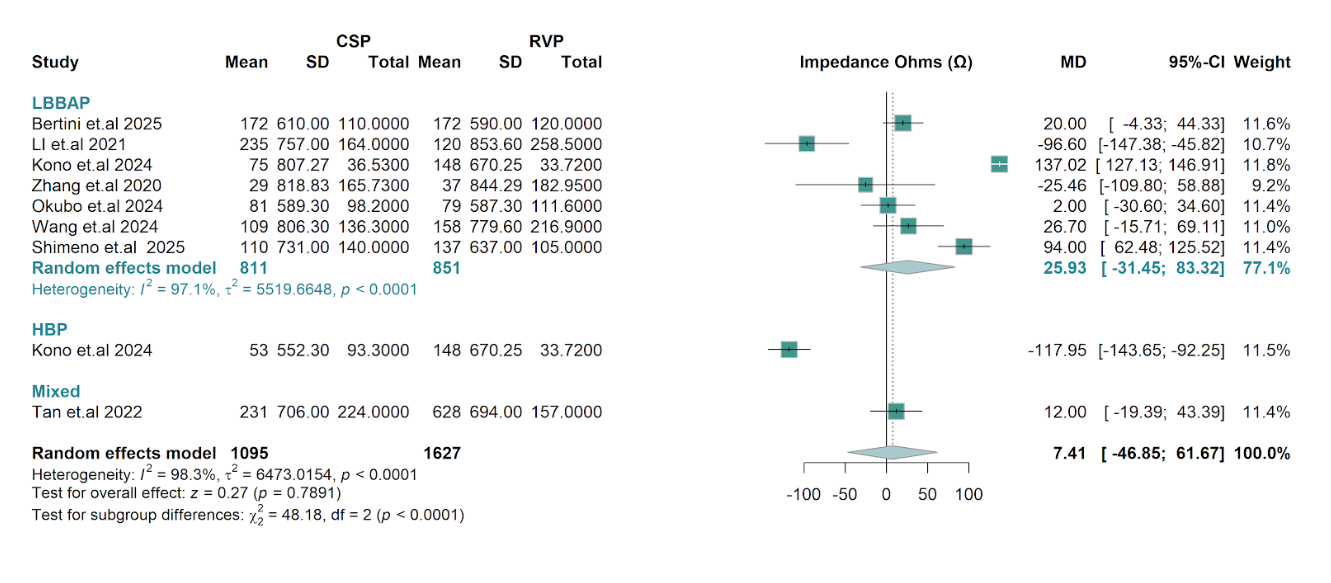


**Figure S79:** Forest Plot of Subgroup Analysis for Impedance Ohms at implantation by CSP Modality


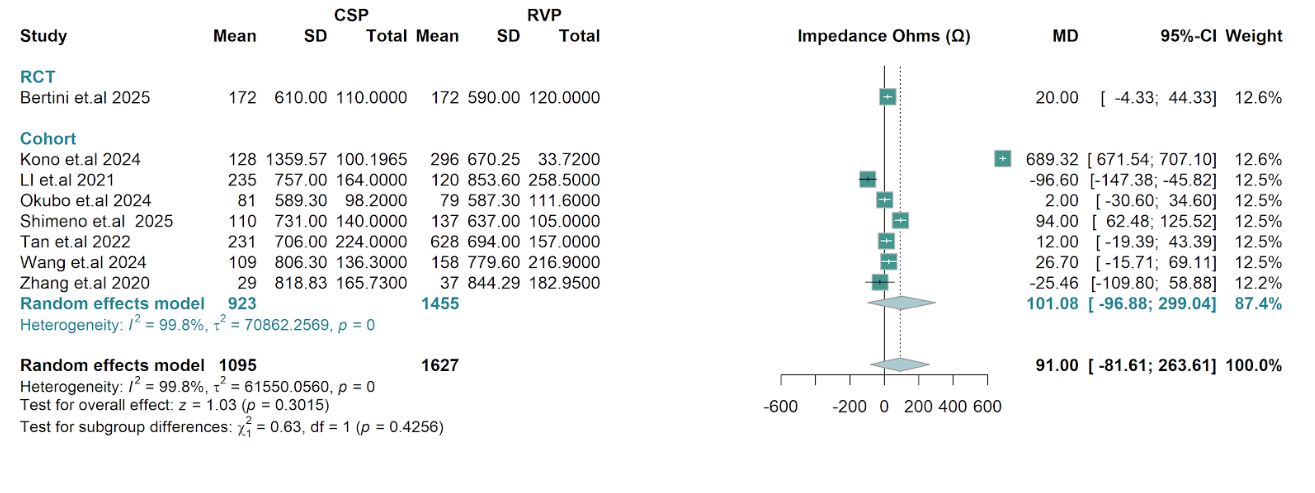


**Figure S80:** Forest Plot of Subgroup Analysis for Impedance Ohms at implantation by Study Design


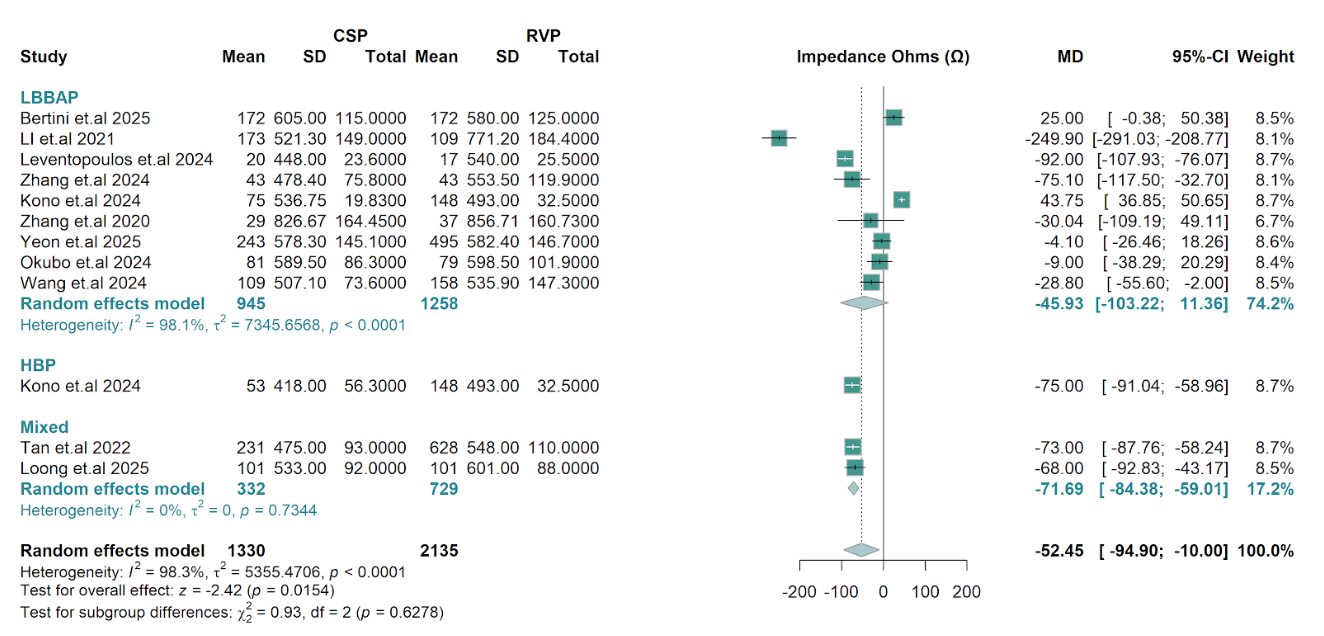


**Figure S81:** Forest Plot of Subgroup Analysis for Impedance Ohms at Follow-up by CSP Modality


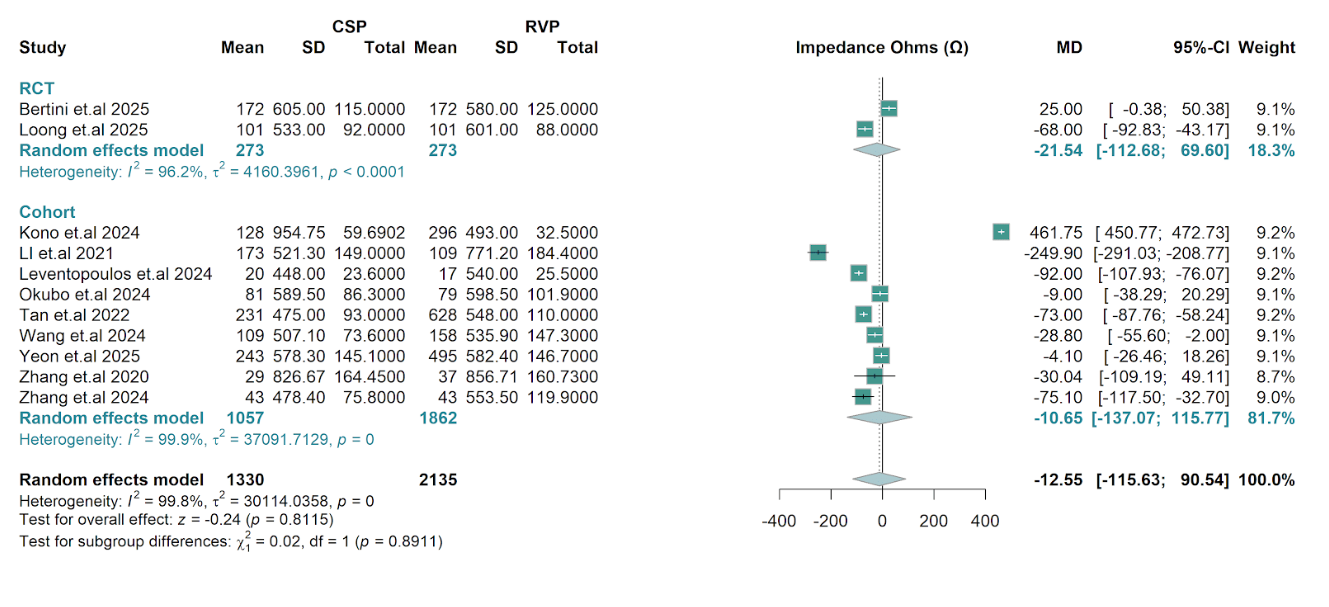


**Figure S82:** Forest Plot of Subgroup Analysis for Impedance Ohms at Follow-up by Study Design


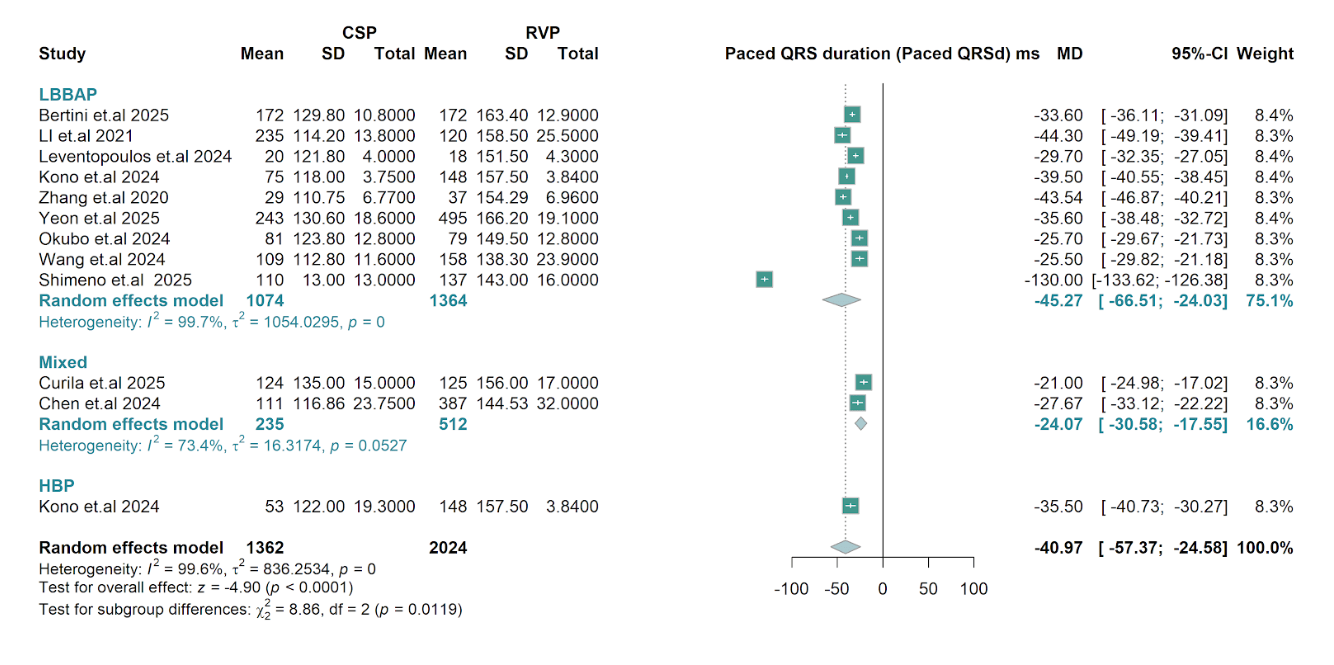


**Figure S83:** Forest Plot of Subgroup Analysis for Paced QRS duration at Implantation by CSP Modality


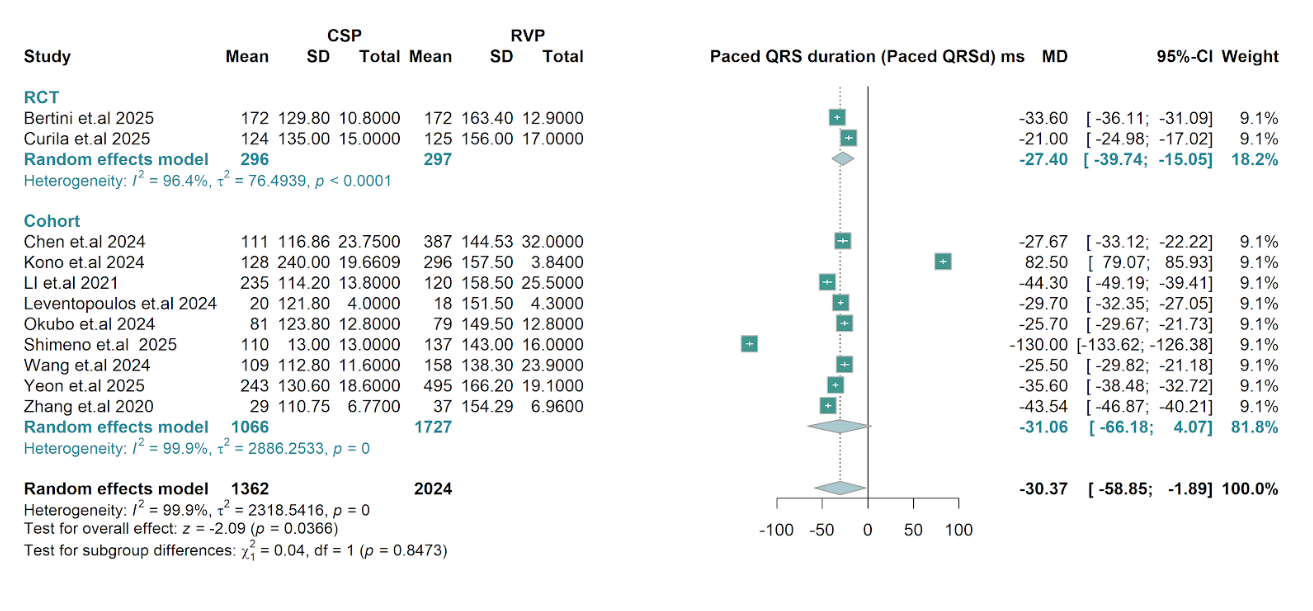


**Figure S84:** Forest Plot of Subgroup Analysis for Paced QRS duration at Implantation by Study Design


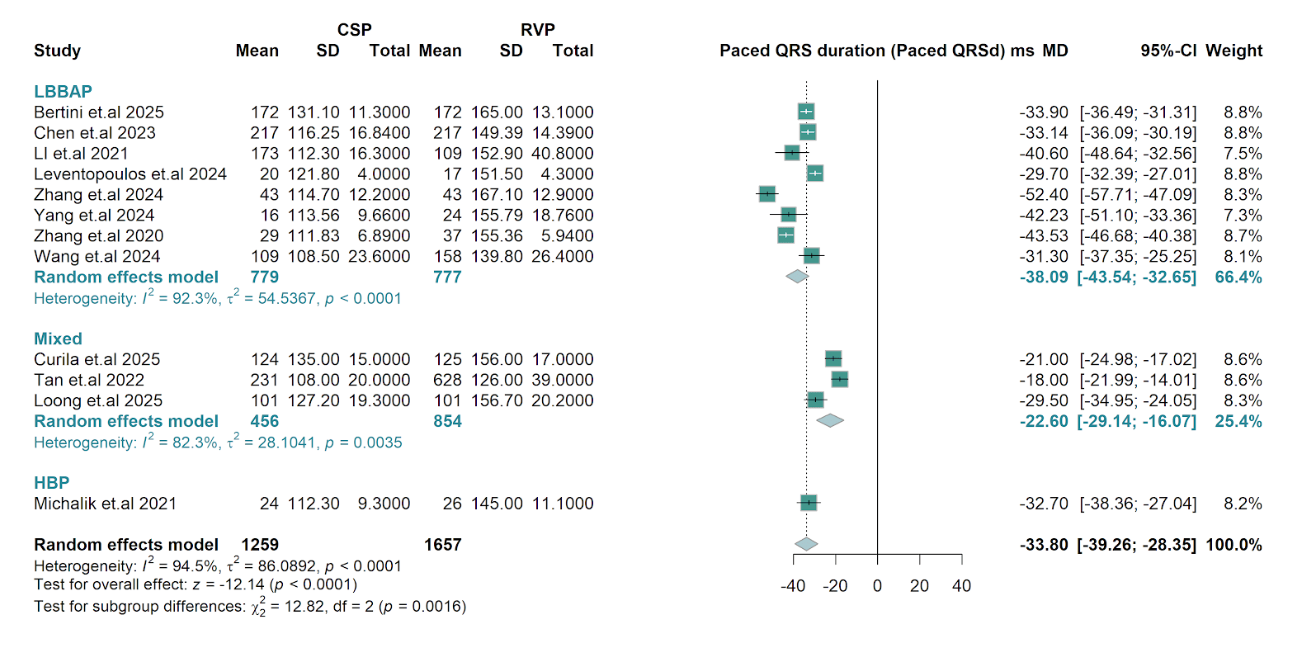


**Figure S85:** Forest Plot of Subgroup Analysis for Paced QRS duration at Follow-up by CSP Modality


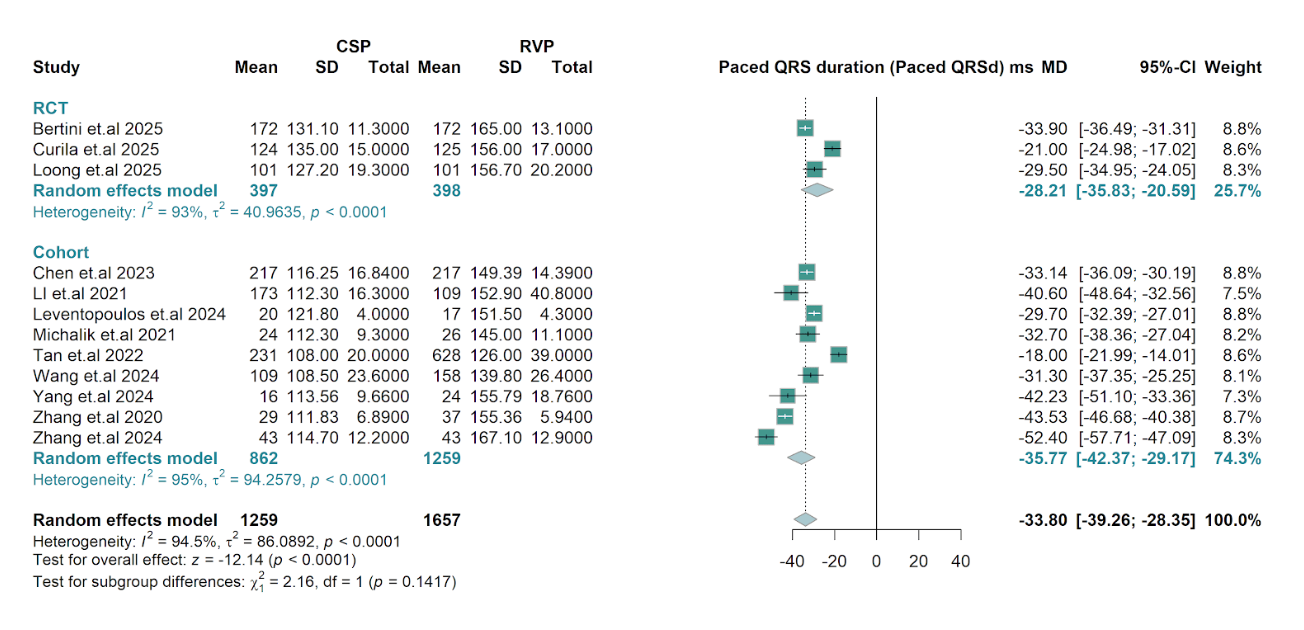


**Figure S86:** Forest Plot of Subgroup Analysis for Paced QRS duration at Follow-up by Study Design


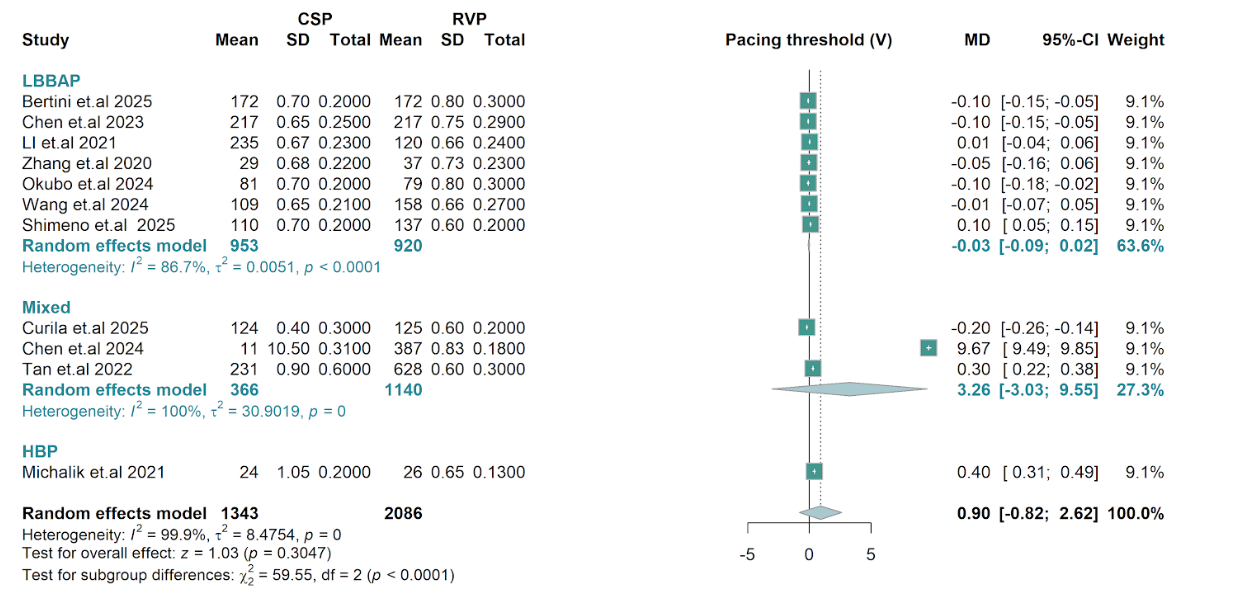


**Figure S87:** Forest Plot of Subgroup Analysis for Pacing threshold (V) at Implantation by CSP Modality


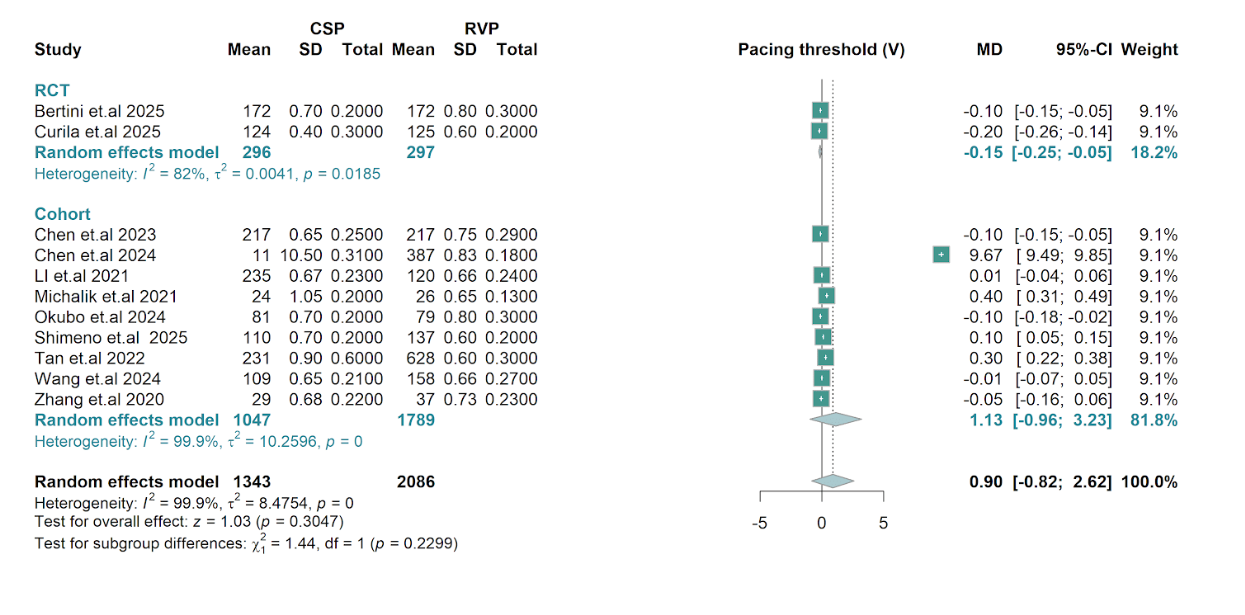


**Figure S88:** Forest Plot of Subgroup Analysis for Pacing threshold (V) at Implantation by Study Design


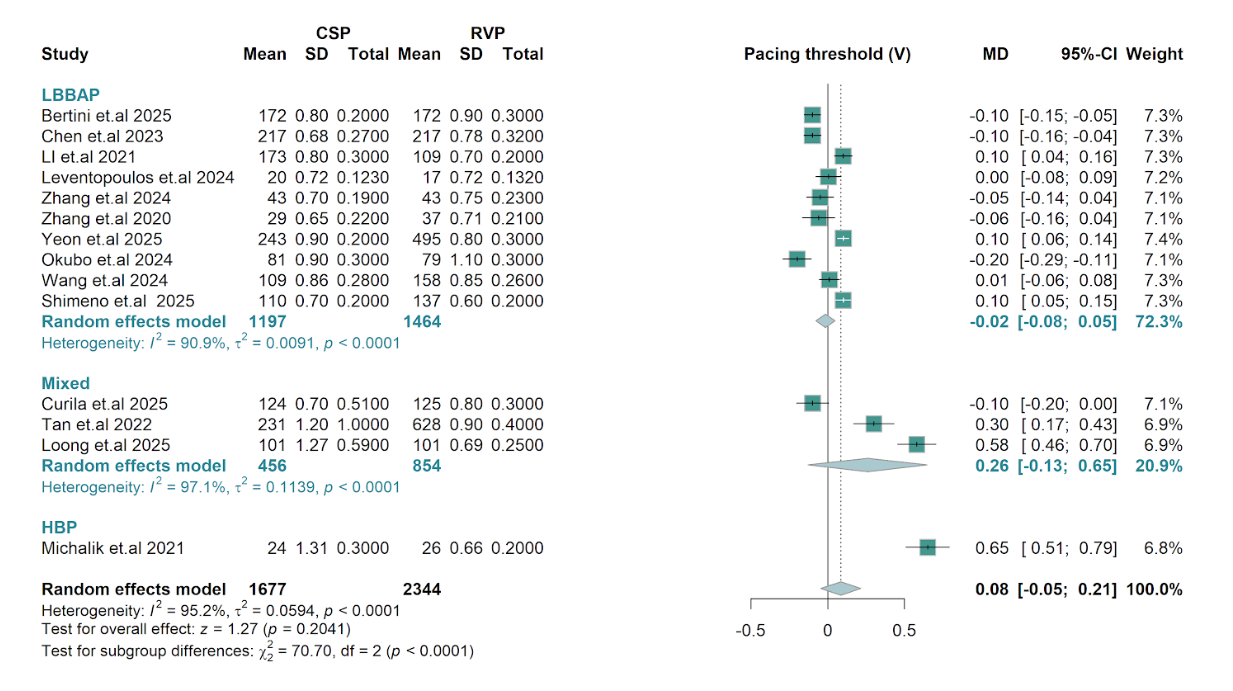


**Figure S89:** Forest Plot of Subgroup Analysis for Pacing threshold (V) at Follow-up by CSP Modality


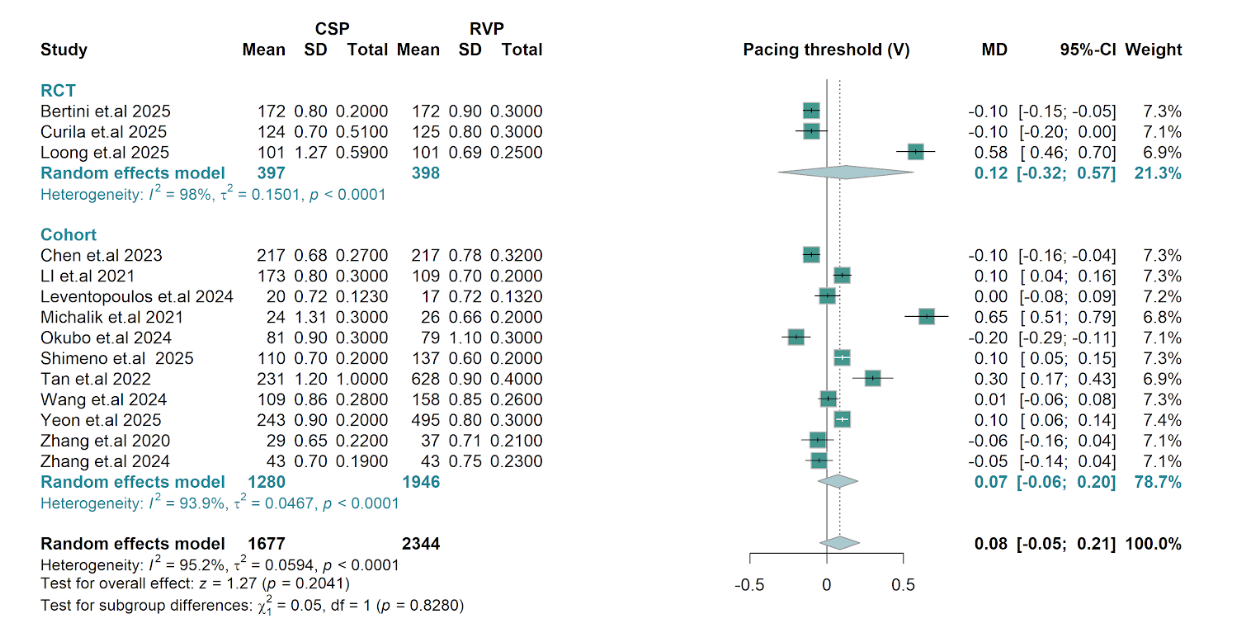


**Figure S90:** Forest Plot of Subgroup Analysis for Pacing threshold (V) at Follow-up by Study Design


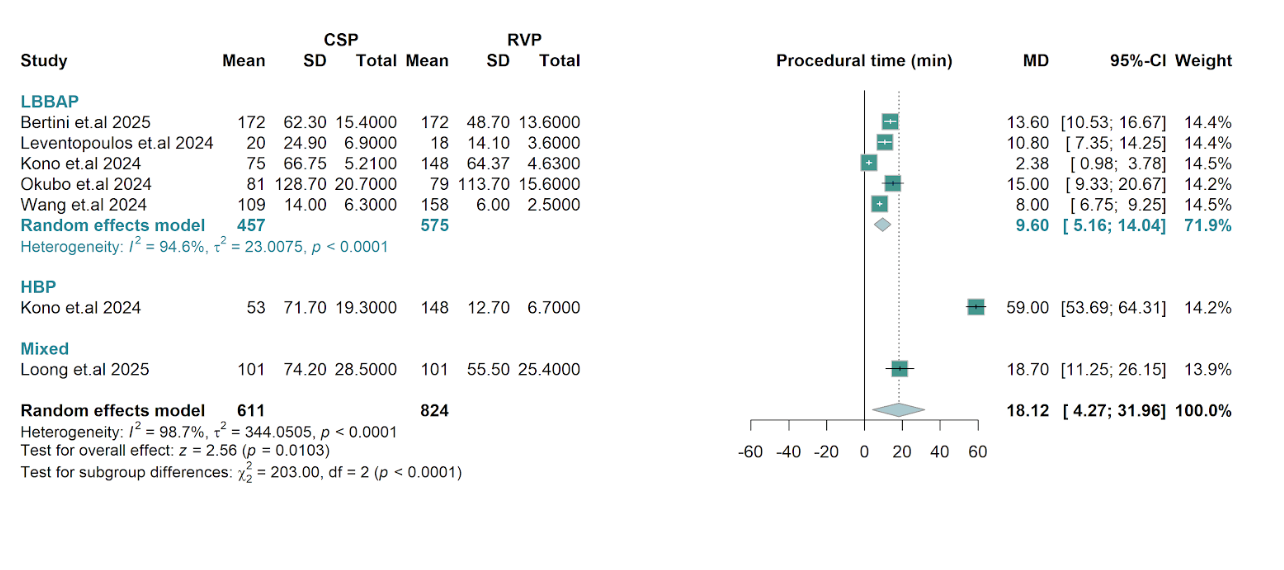


**Figure S91:** Forest Plot of Subgroup Analysis for Procedural time (min) at Implantation by CSP Modality


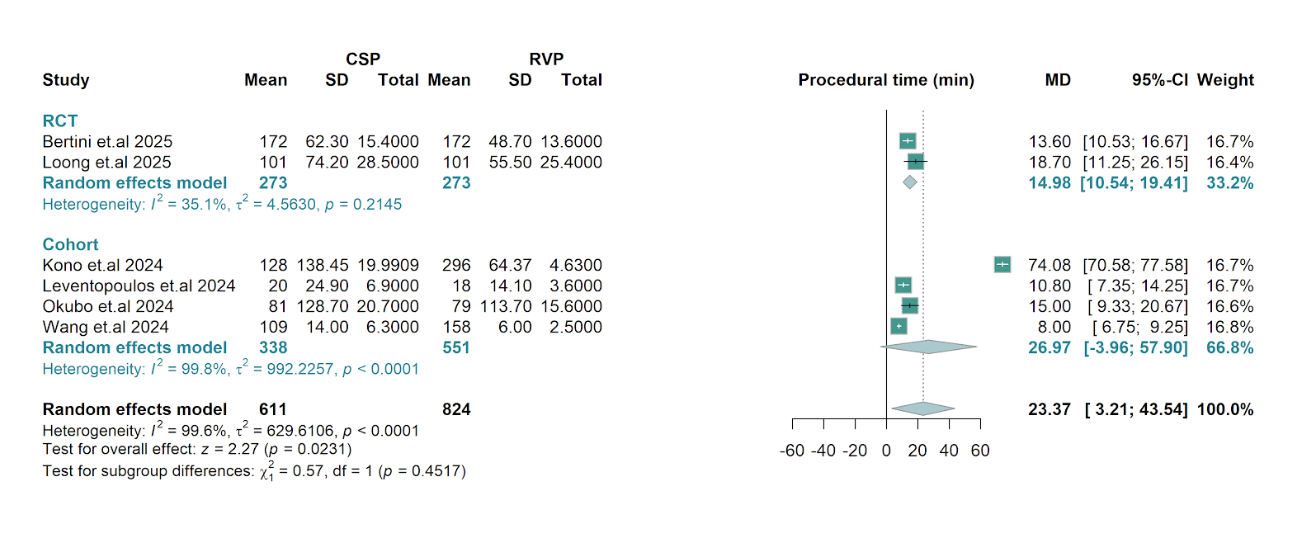


**Figure S92:** Forest Plot of Subgroup Analysis for Procedural time (min) at Implantation by Study Design


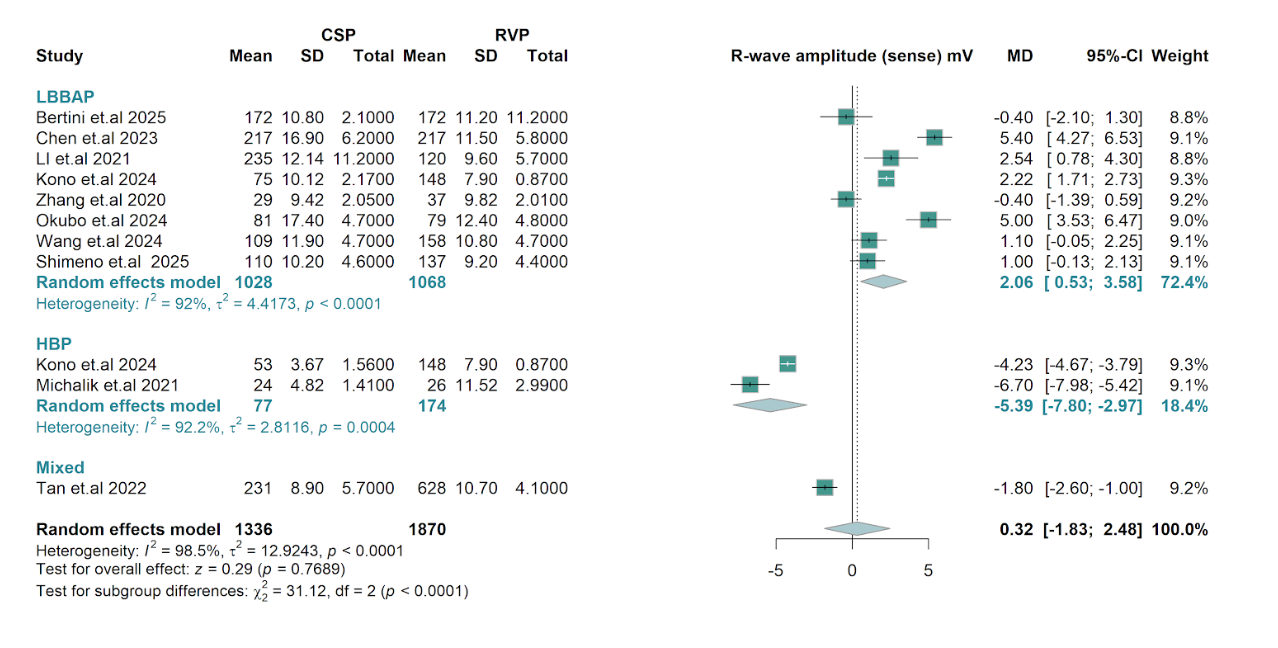


**Figure S93:** Forest Plot of Subgroup Analysis for R-wave amplitude at Implantation by CSP Modality


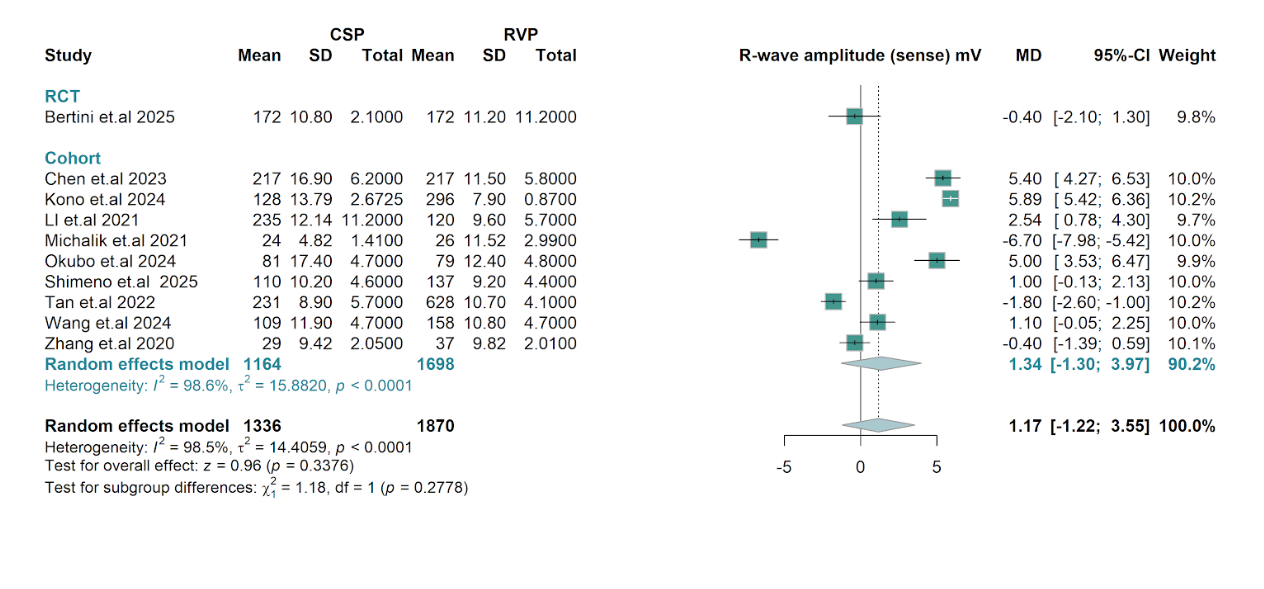


**Figure S94:** Forest Plot of Subgroup Analysis for R-wave amplitude at Implantation by Study Design


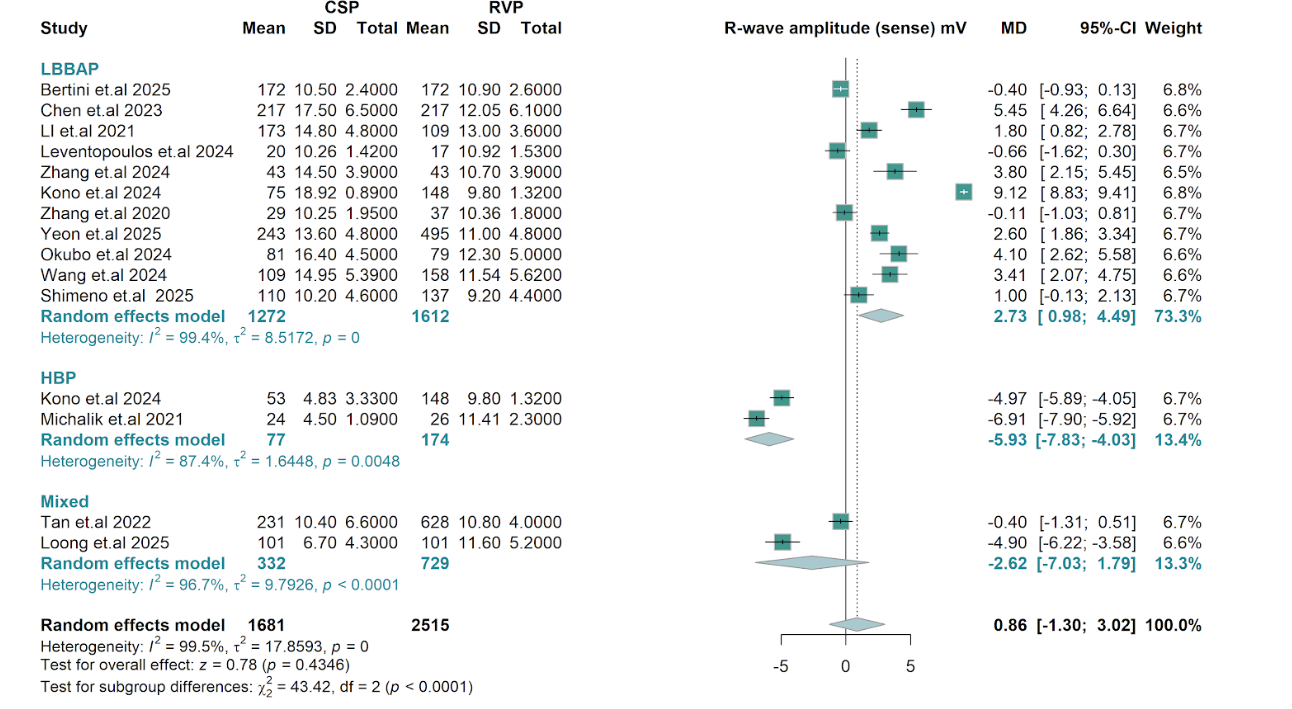


**Figure S95:** Forest Plot of Subgroup Analysis for R-wave amplitude at Follow-up by CSP Modality


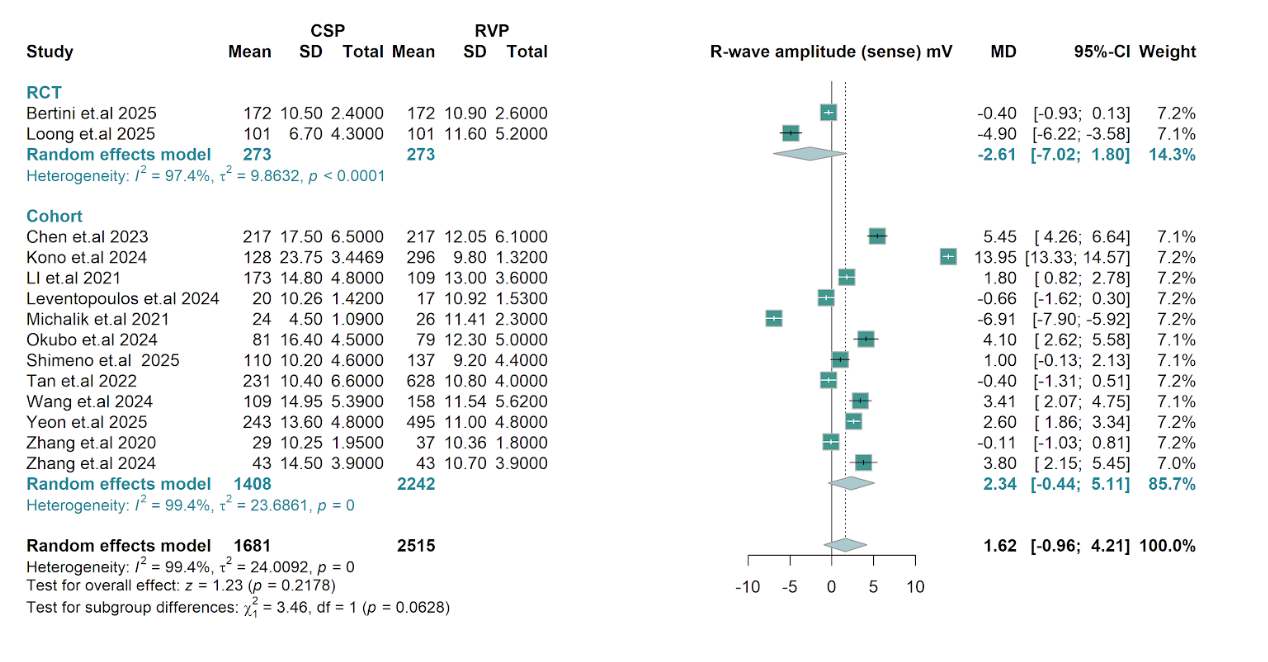


**Figure S96:** Forest Plot of Subgroup Analysis for R-wave amplitude at Follow-up by Study Design


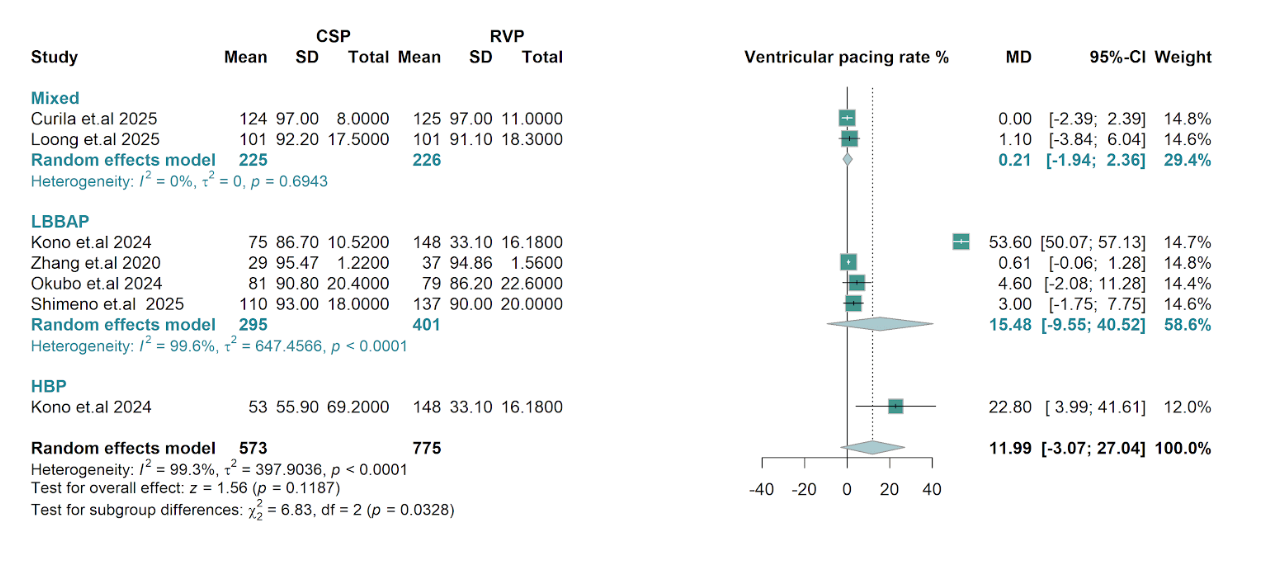


**Figure S97:** Forest Plot of Subgroup Analysis for Ventricular pacing rate at Follow-up by CSP Modality


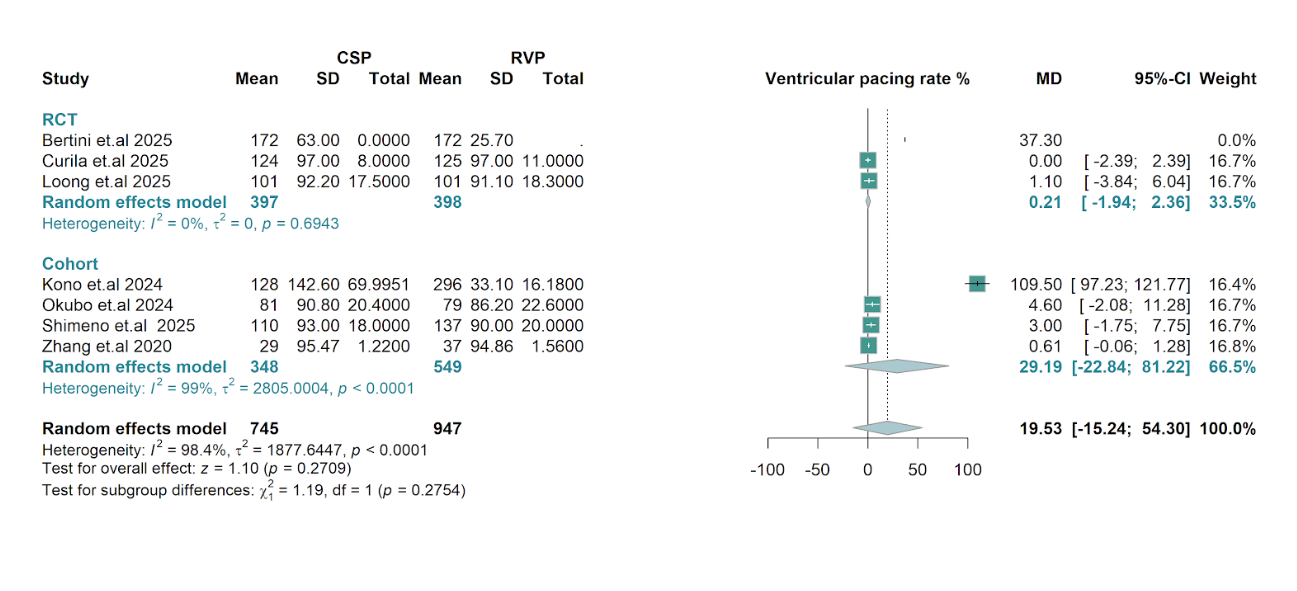


**Figure S98:** Forest Plot of Subgroup Analysis for Ventricular pacing rate at follow-up by Study Design


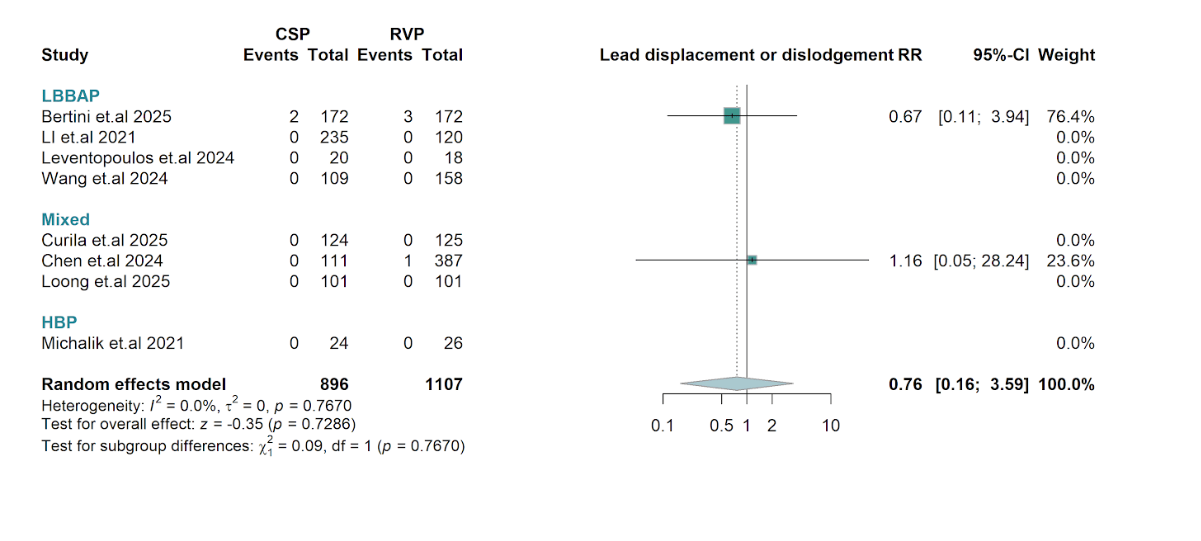


**Figure S99:** Forest Plot of Subgroup Analysis for Lead displacement at Implantation by CSP Modality

**Figure S100:** Forest Plot of Subgroup Analysis for Lead displacement at Implantation by Study Design

**Figure S101:** Forest Plot of Subgroup Analysis for Lead displacement at Follow-up by CSP Modality

**Figure S102:** Forest Plot of Subgroup Analysis for Lead displacement at Follow-up by Study Design
